# Supplementary figures and images for: A neuroligin-2-YAP axis regulates progression of pancreatic intraepithelial neoplasia (part 1 of 2)
Source: EMBO Rep. 2024 Feb 27;25(4):17. doi: 10.1038/s44319-024-00104-x (PMC11014856; doi:10.1038/s44319-024-00104-x)

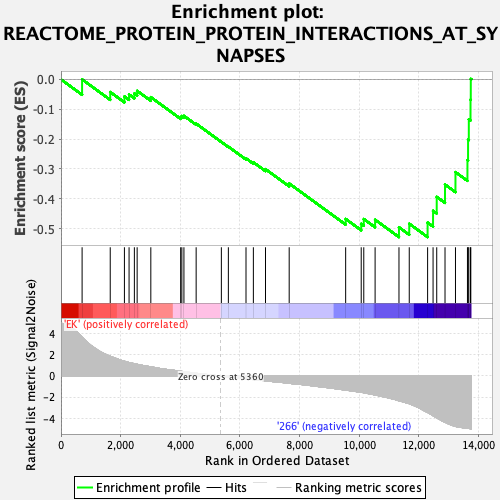

Supplement: Supplementary file 1 — Source Data Fig. 1 [file 44319_2024_104_MOESM1_ESM.zip › Figure 1/1A/enplot_REACTOME_PROTEIN_PROTEIN_INTERACTIONS_AT_SYNAPSES_225.png]

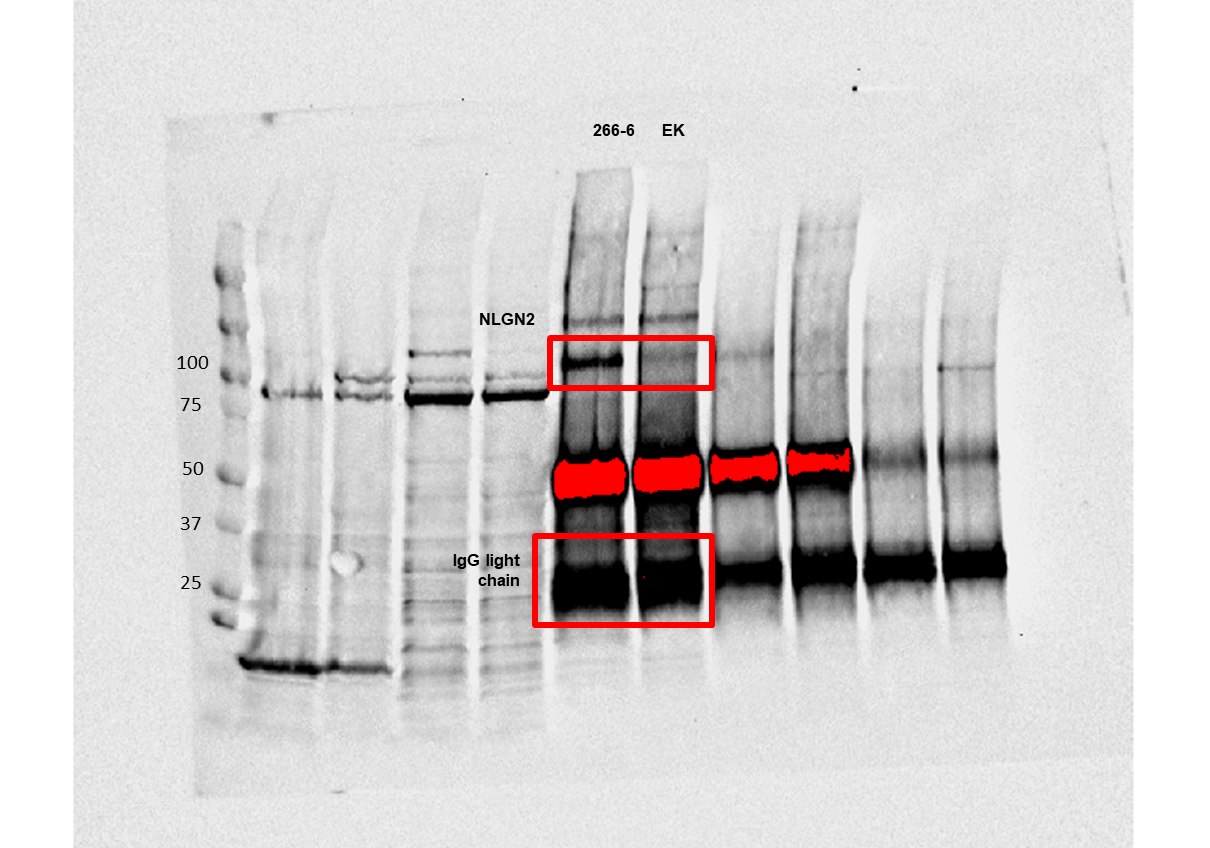

Supplement: Supplementary file 1 — Source Data Fig. 1 [file 44319_2024_104_MOESM1_ESM.zip › Figure 1/1A/WB NLGN2 IgG 266-6 EK.tif]

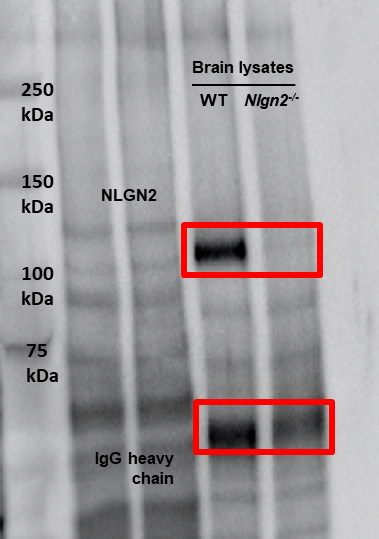

Supplement: Supplementary file 1 — Source Data Fig. 1 [file 44319_2024_104_MOESM1_ESM.zip › Figure 1/1A/WB NLGN2 IgG Brain.tif]

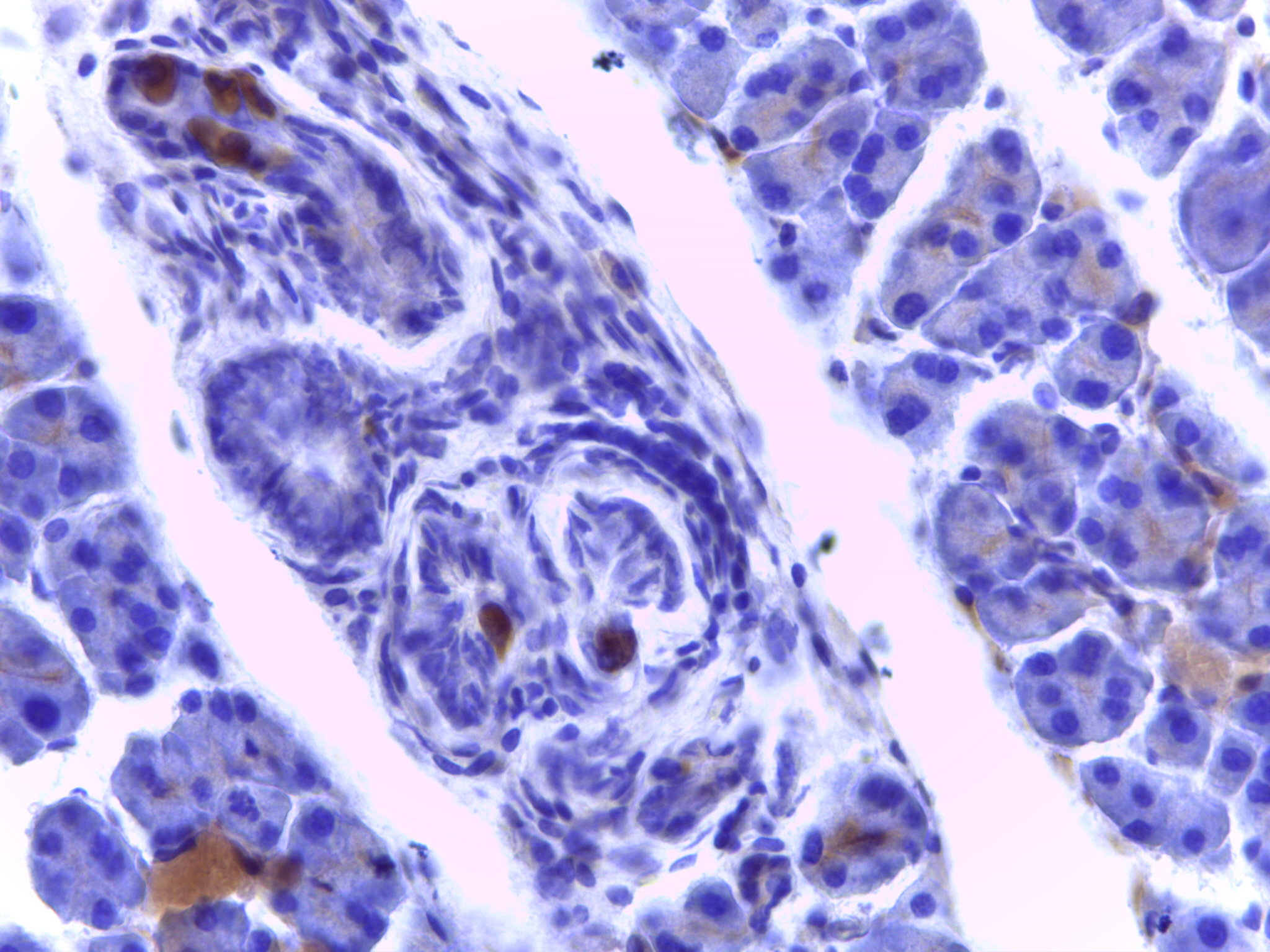

Supplement: Supplementary file 1 — Source Data Fig. 1 [file 44319_2024_104_MOESM1_ESM.zip › Figure 1/1B/Elast high.jpg]

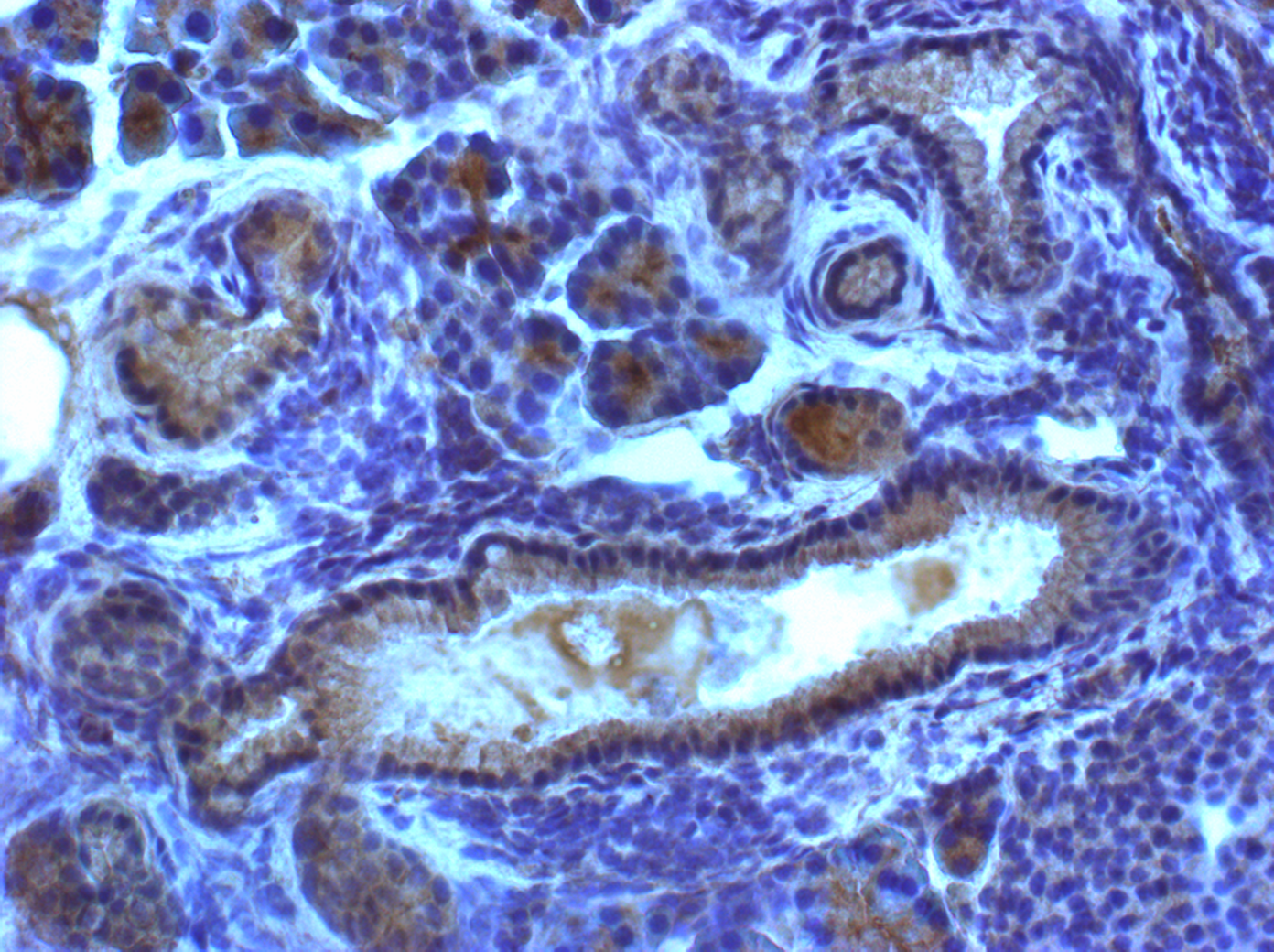

Supplement: Supplementary file 1 — Source Data Fig. 1 [file 44319_2024_104_MOESM1_ESM.zip › Figure 1/1B/Elast low.tif]

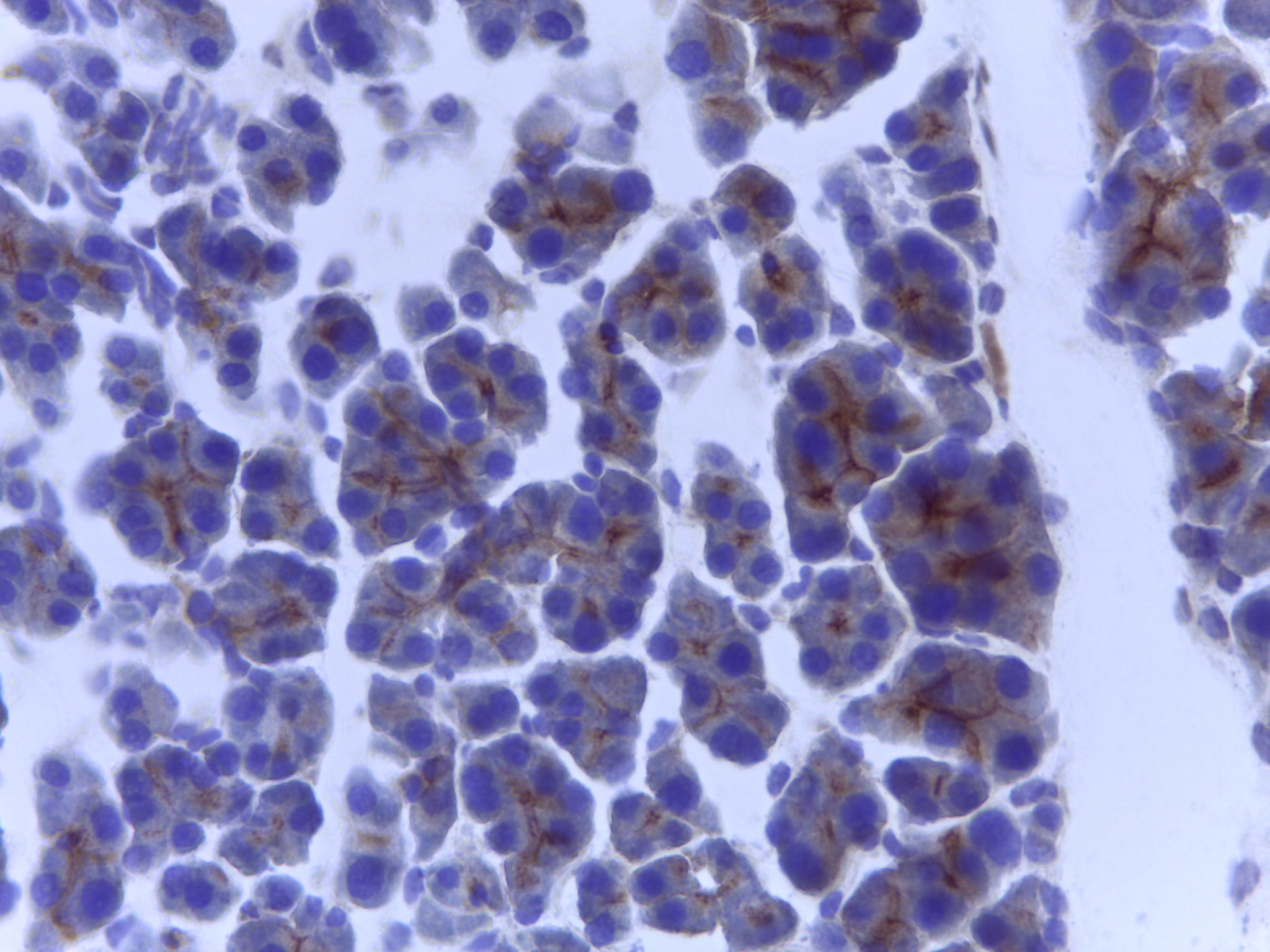

Supplement: Supplementary file 1 — Source Data Fig. 1 [file 44319_2024_104_MOESM1_ESM.zip › Figure 1/1B/Elast normal.jpg]

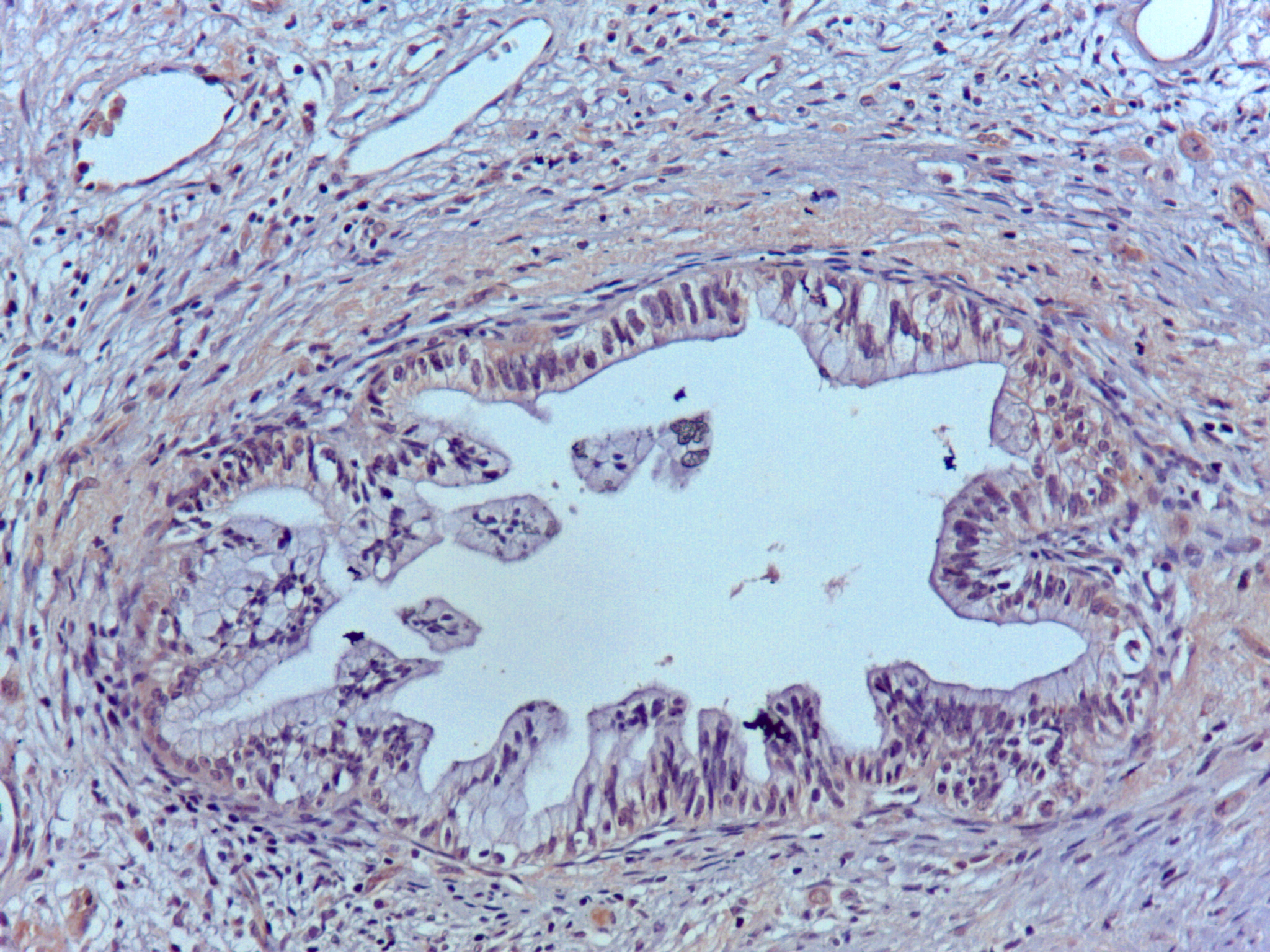

Supplement: Supplementary file 1 — Source Data Fig. 1 [file 44319_2024_104_MOESM1_ESM.zip › Figure 1/1B/Human high.jpg]

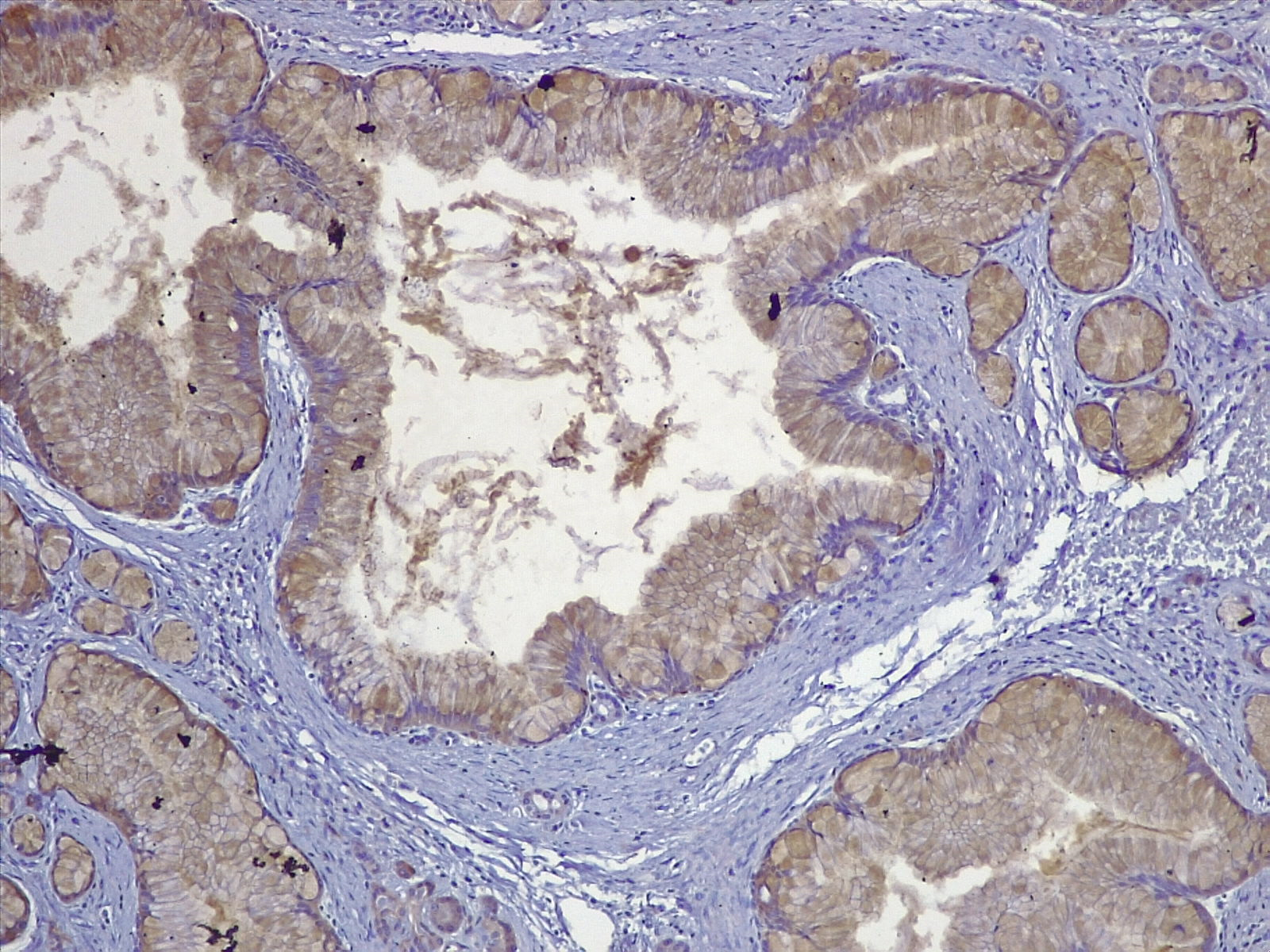

Supplement: Supplementary file 1 — Source Data Fig. 1 [file 44319_2024_104_MOESM1_ESM.zip › Figure 1/1B/Human low.jpg]

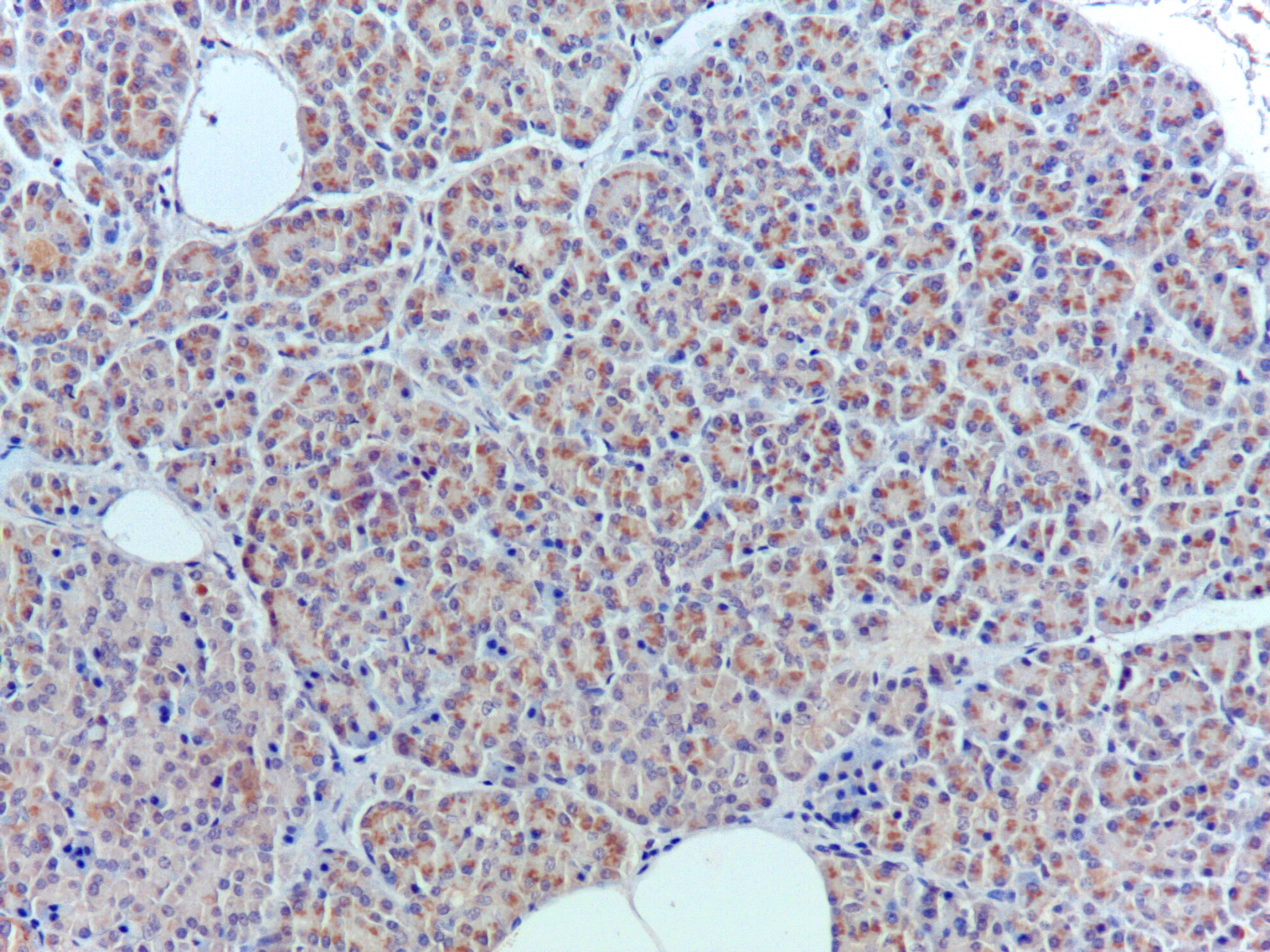

Supplement: Supplementary file 1 — Source Data Fig. 1 [file 44319_2024_104_MOESM1_ESM.zip › Figure 1/1B/Human normal.jpg]

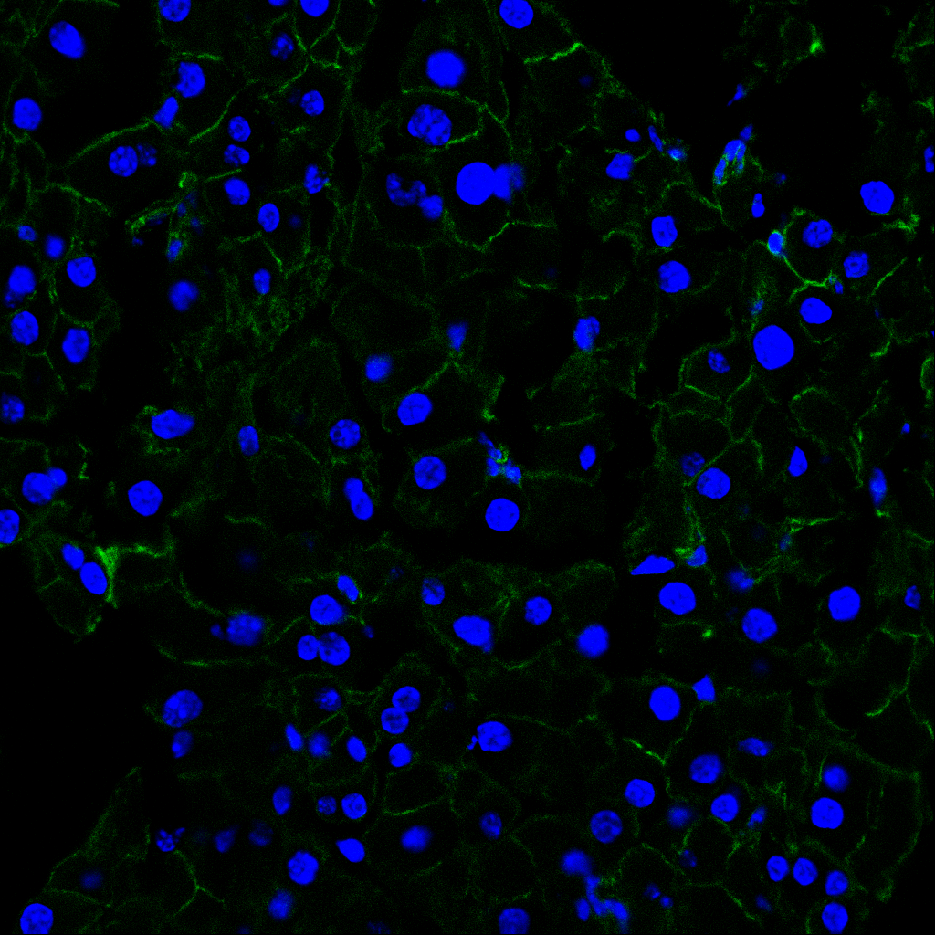

Supplement: Supplementary file 1 — Source Data Fig. 1 [file 44319_2024_104_MOESM1_ESM.zip › Figure 1/1C/Ecad.tif]

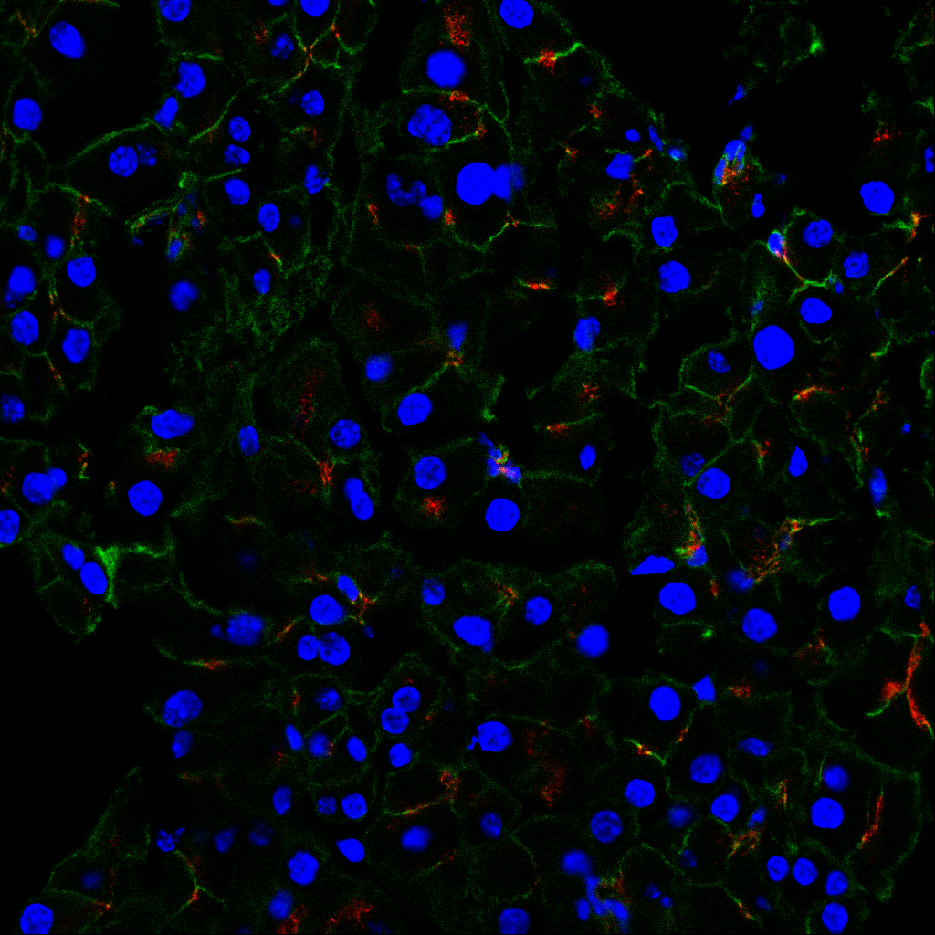

Supplement: Supplementary file 1 — Source Data Fig. 1 [file 44319_2024_104_MOESM1_ESM.zip › Figure 1/1C/Merge Ecad.tif]

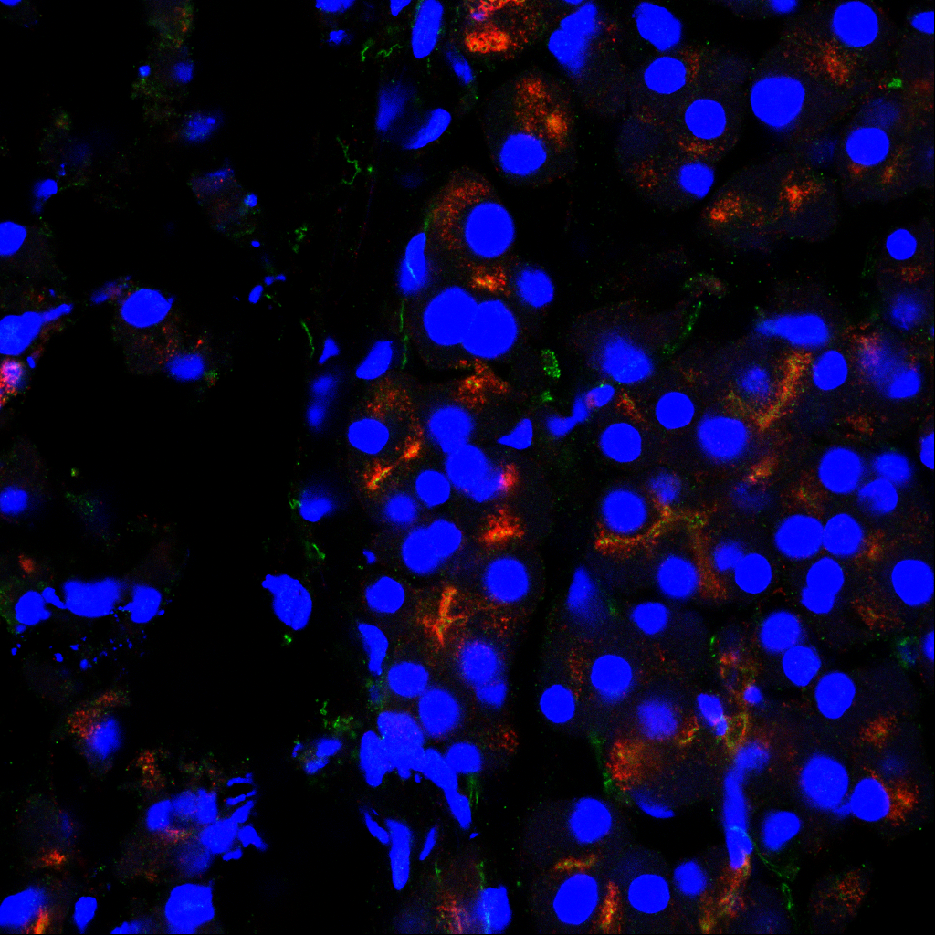

Supplement: Supplementary file 1 — Source Data Fig. 1 [file 44319_2024_104_MOESM1_ESM.zip › Figure 1/1C/Merge Zo1.tif]

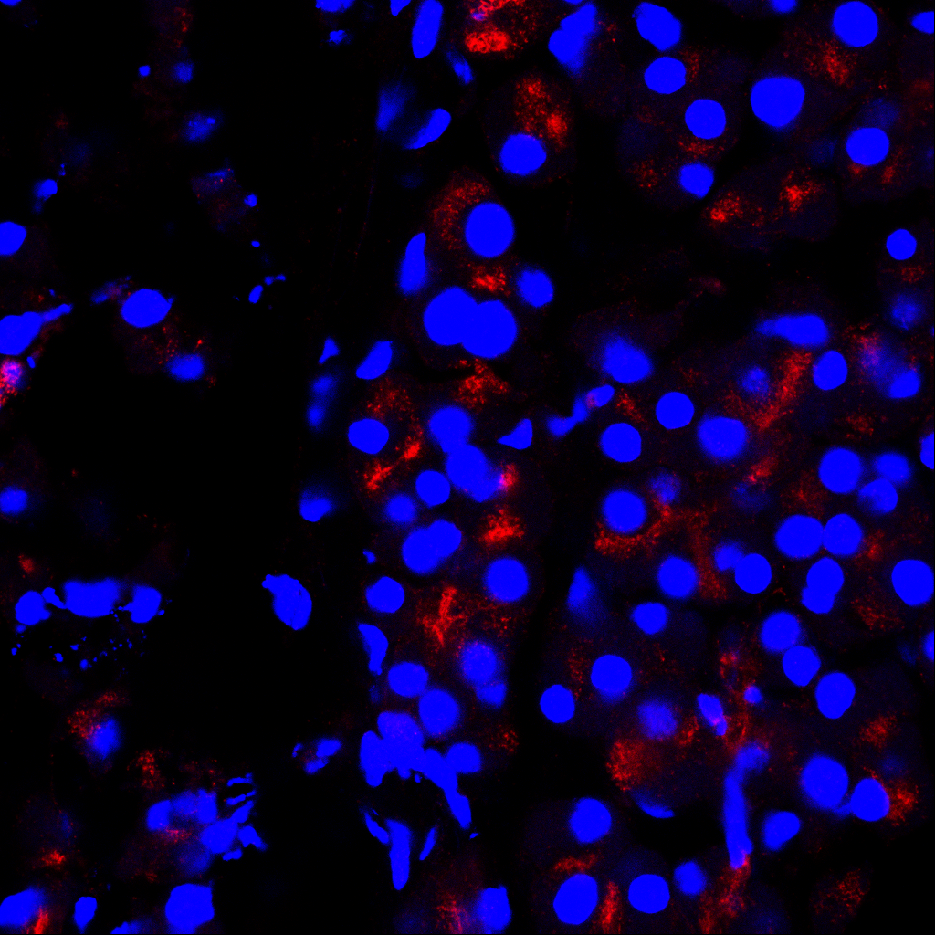

Supplement: Supplementary file 1 — Source Data Fig. 1 [file 44319_2024_104_MOESM1_ESM.zip › Figure 1/1C/Nlgn2 left.tif]

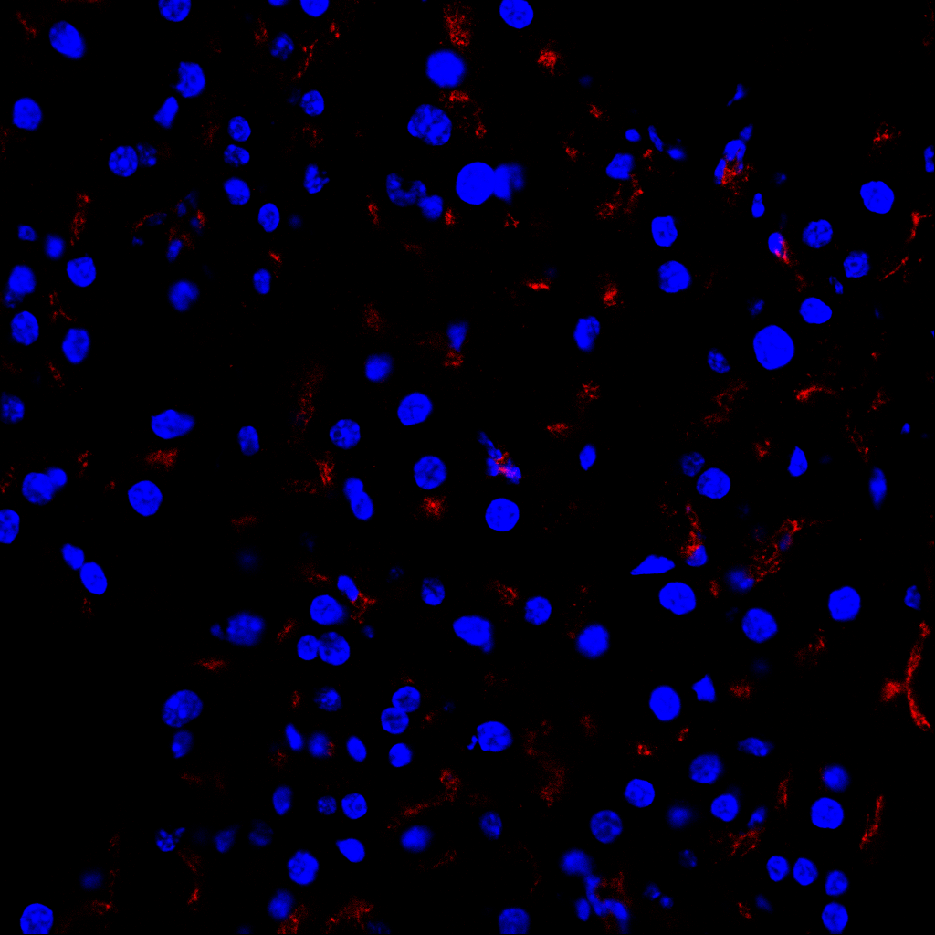

Supplement: Supplementary file 1 — Source Data Fig. 1 [file 44319_2024_104_MOESM1_ESM.zip › Figure 1/1C/Nlgn2 right.tif]

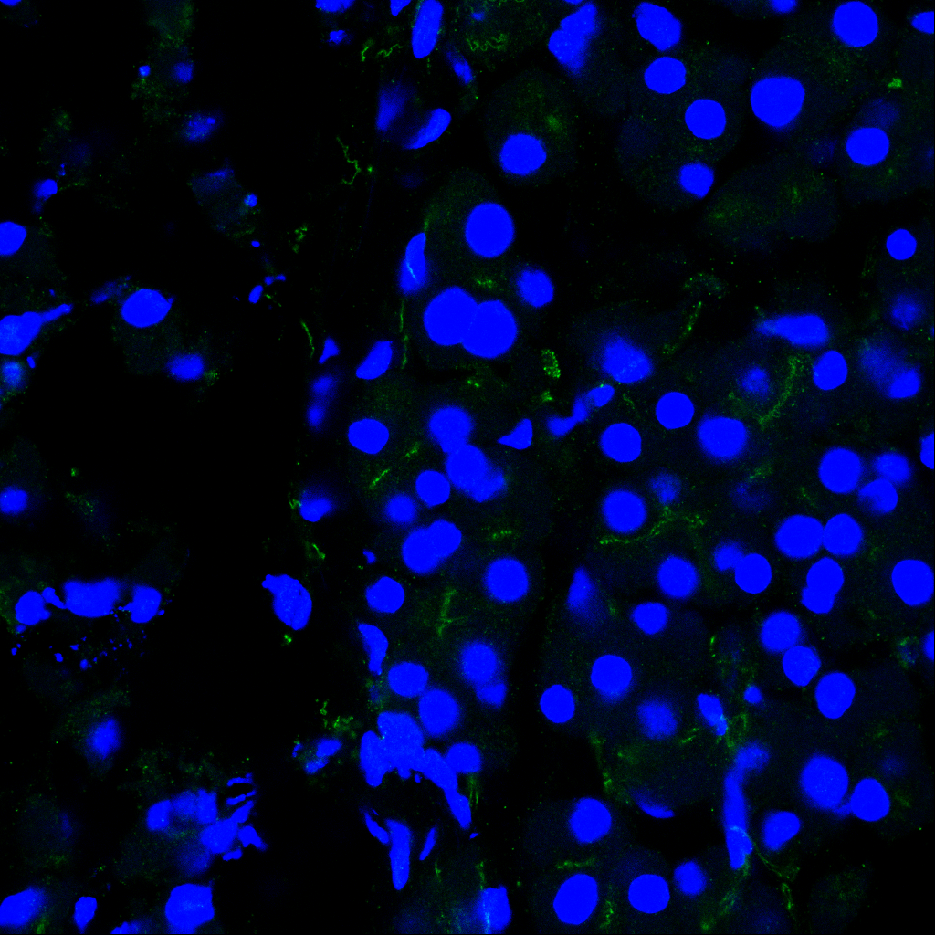

Supplement: Supplementary file 1 — Source Data Fig. 1 [file 44319_2024_104_MOESM1_ESM.zip › Figure 1/1C/Zo1.tif]

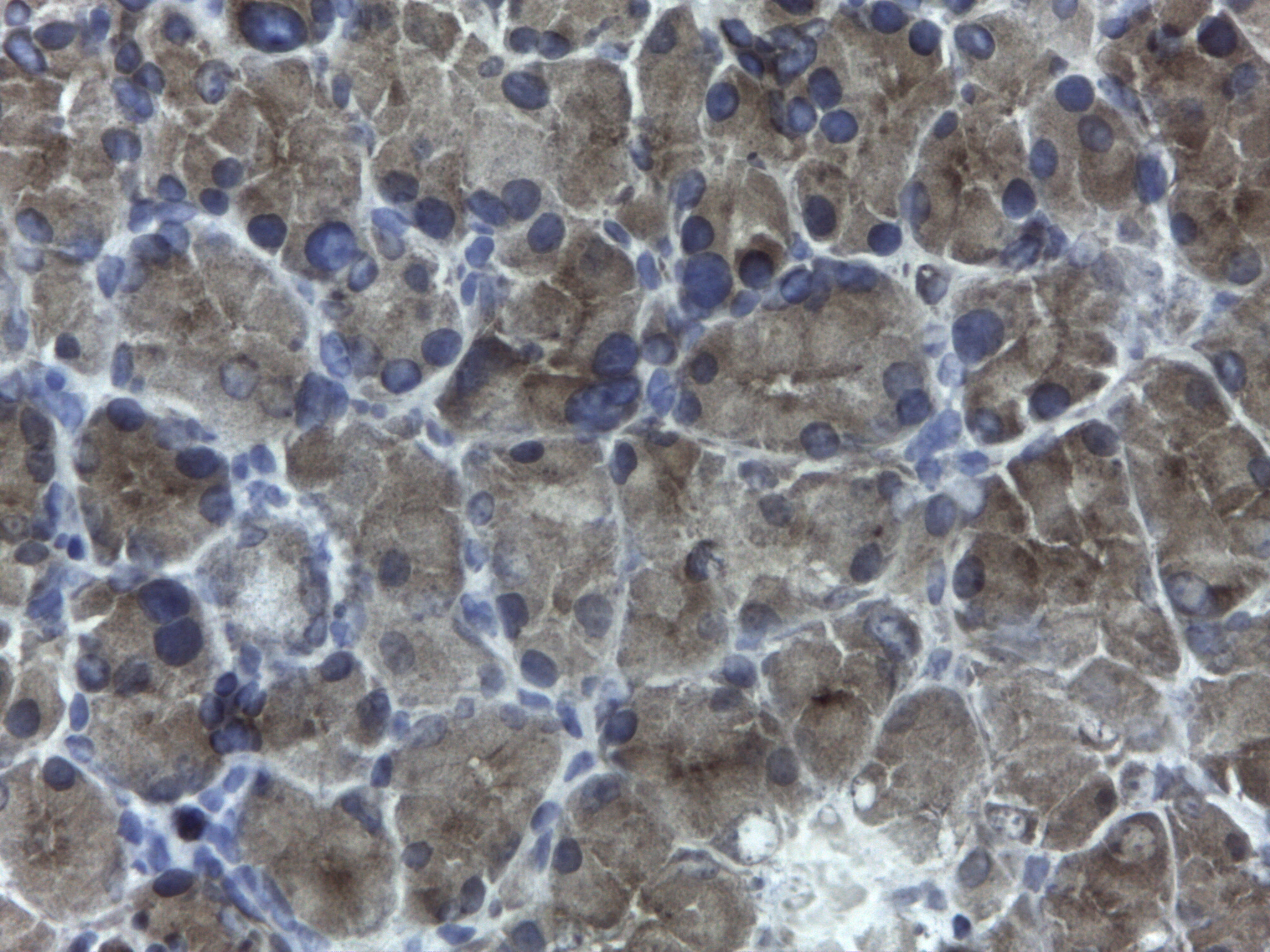

Supplement: Supplementary file 1 — Source Data Fig. 1 [file 44319_2024_104_MOESM1_ESM.zip › Figure 1/1D/Elast ceru 3m recovery.jpg]

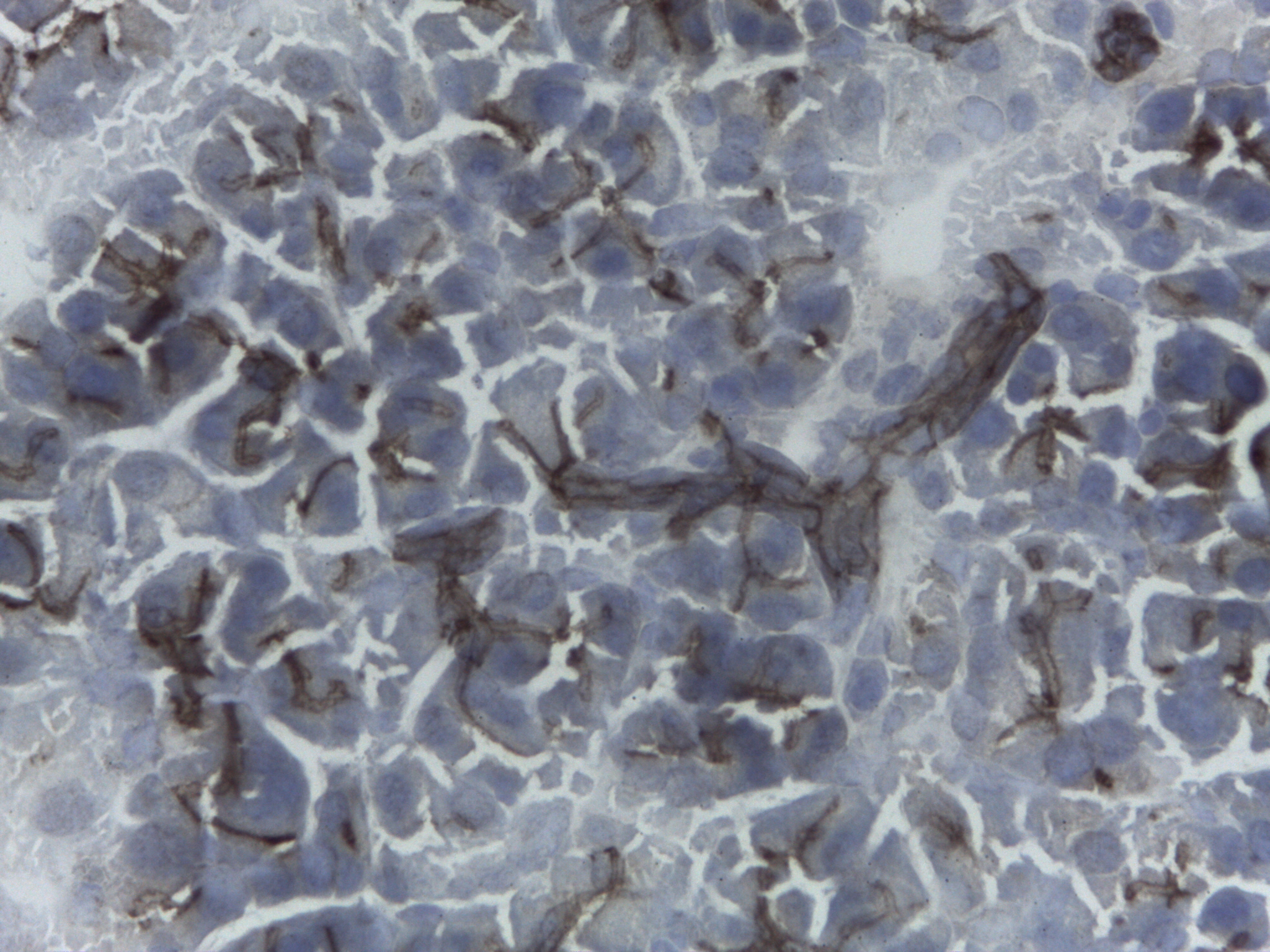

Supplement: Supplementary file 1 — Source Data Fig. 1 [file 44319_2024_104_MOESM1_ESM.zip › Figure 1/1D/Elast ceru 8m recovery.jpg]

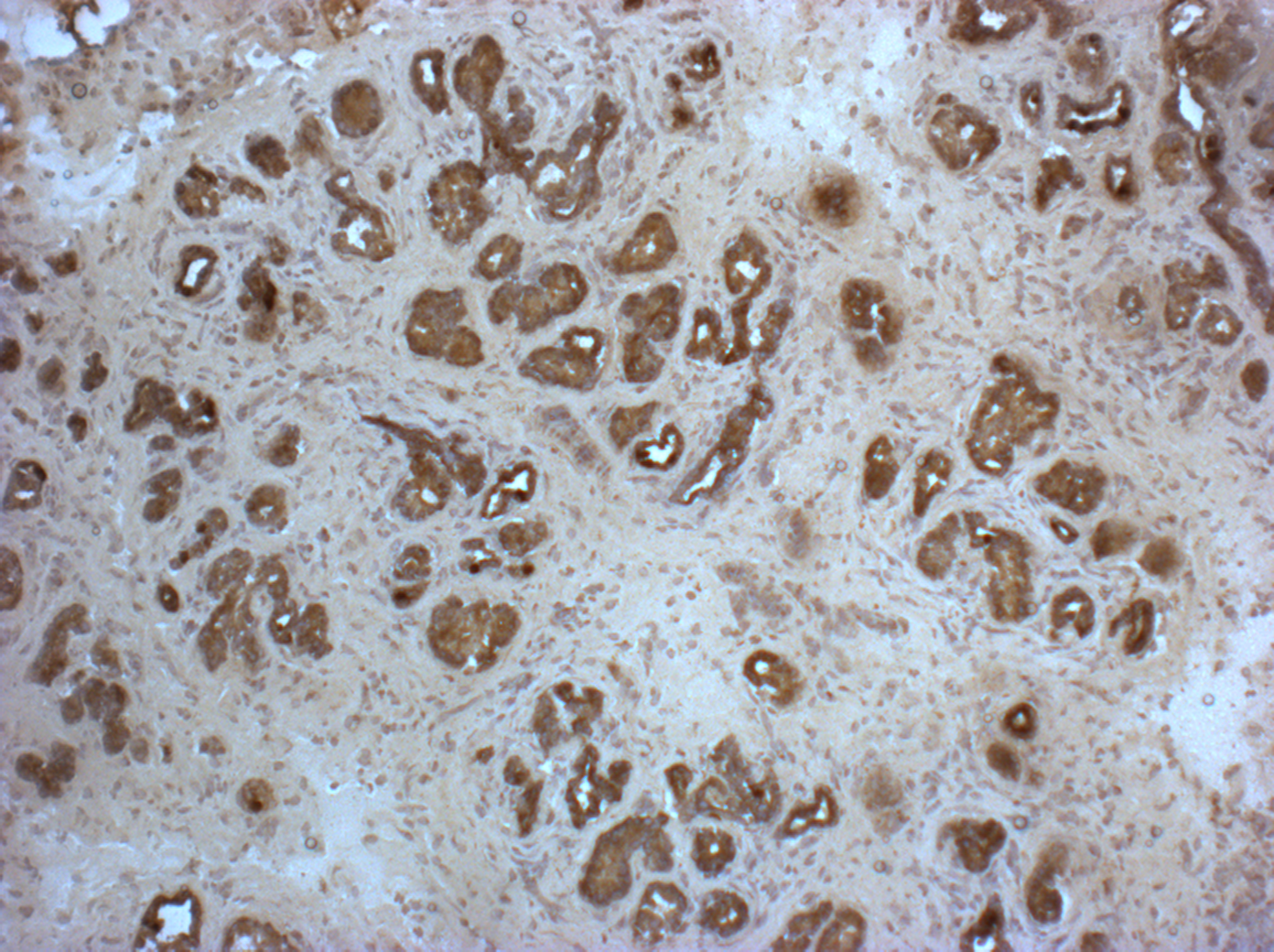

Supplement: Supplementary file 1 — Source Data Fig. 1 [file 44319_2024_104_MOESM1_ESM.zip › Figure 1/1D/Elast ceru.tif]

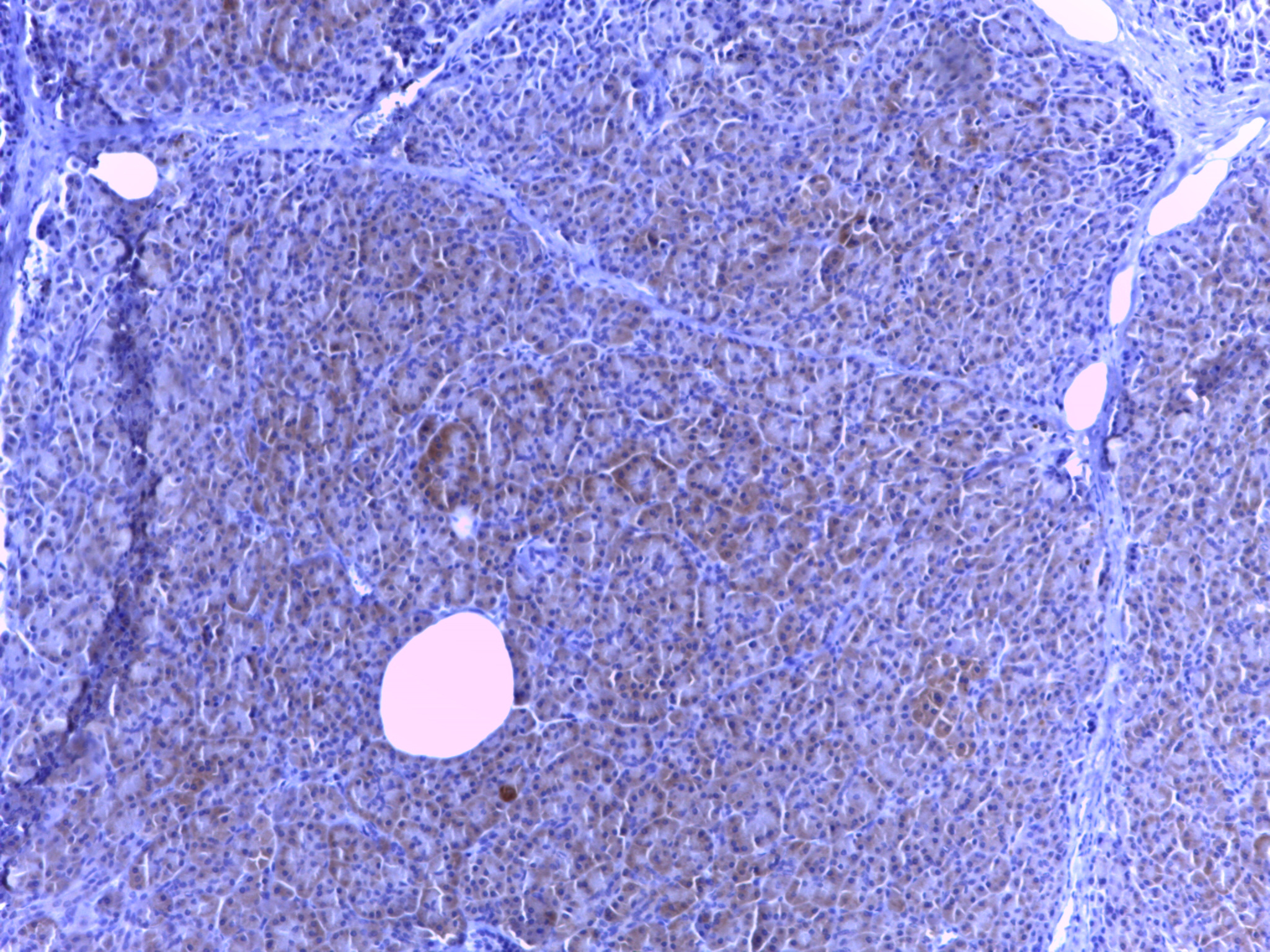

Supplement: Supplementary file 1 — Source Data Fig. 1 [file 44319_2024_104_MOESM1_ESM.zip › Figure 1/1D/Human normal.jpg]

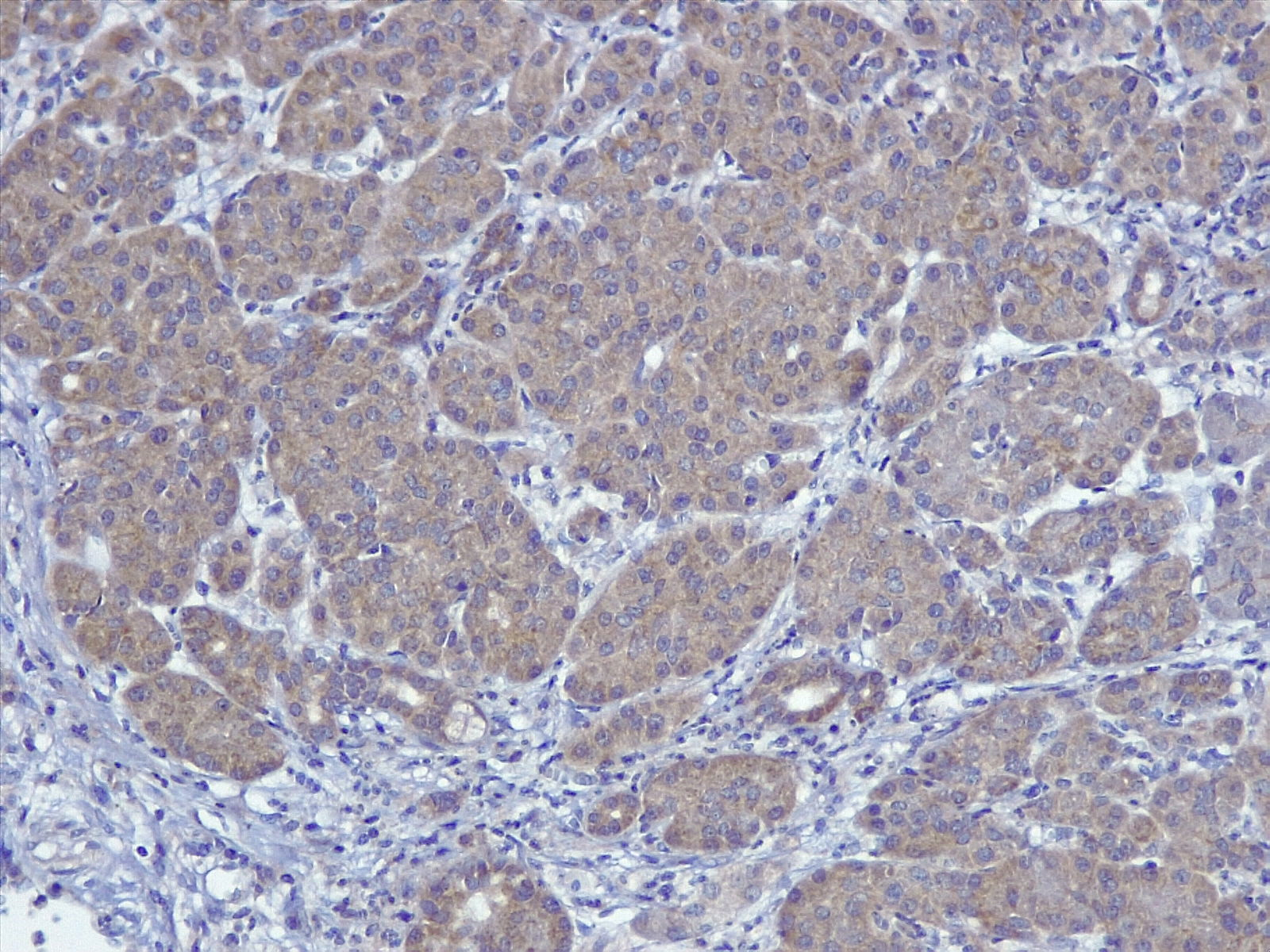

Supplement: Supplementary file 1 — Source Data Fig. 1 [file 44319_2024_104_MOESM1_ESM.zip › Figure 1/1D/Human pancreatitis.jpg]

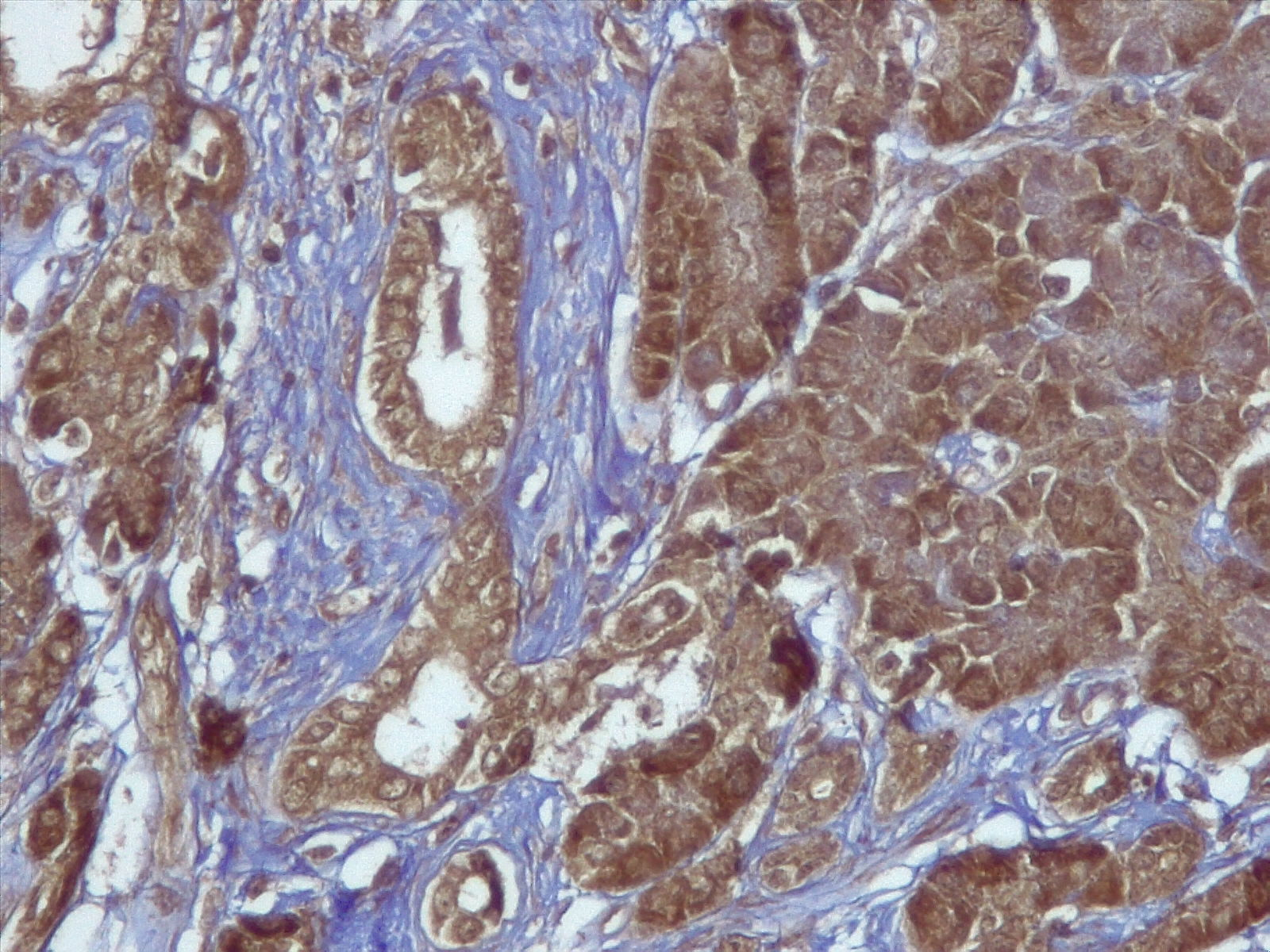

Supplement: Supplementary file 1 — Source Data Fig. 1 [file 44319_2024_104_MOESM1_ESM.zip › Figure 1/1D/Human PD patient.jpg]

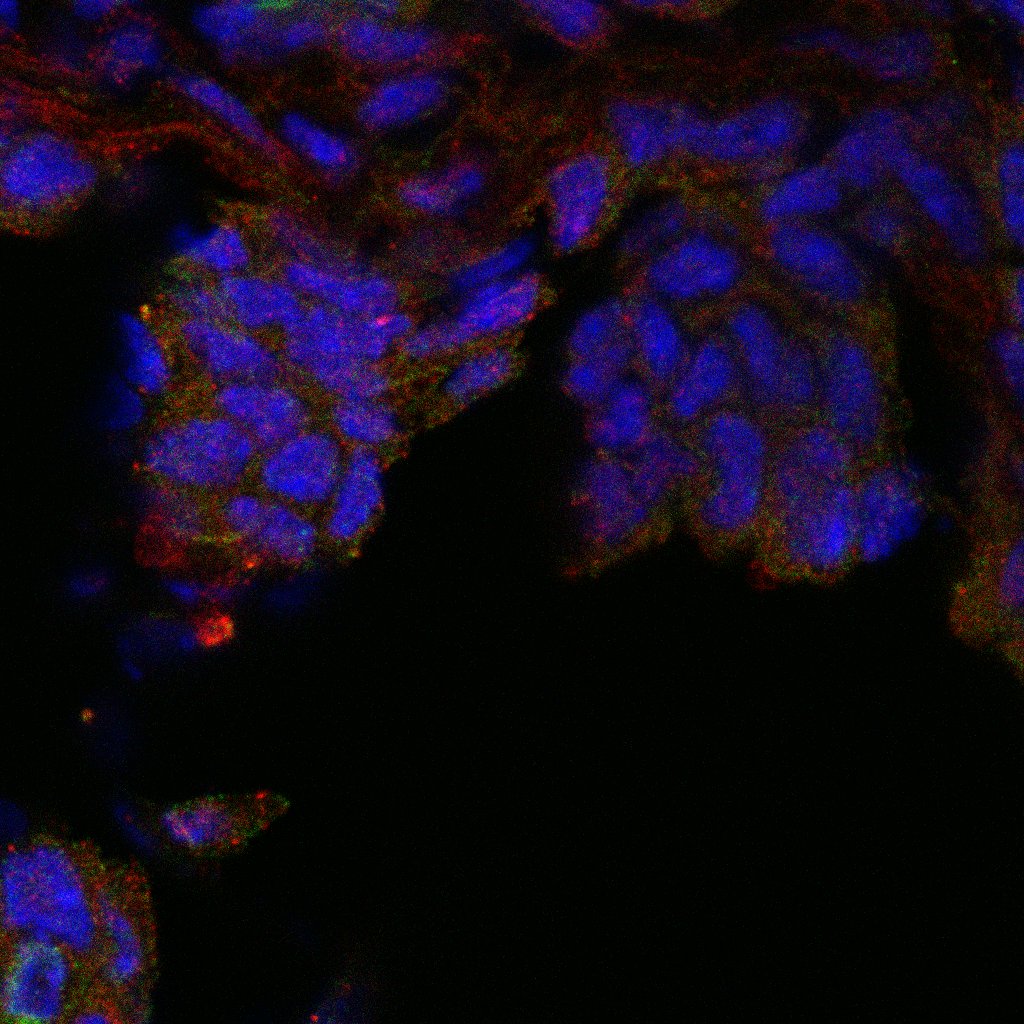

Supplement: Supplementary file 1 — Source Data Fig. 1 [file 44319_2024_104_MOESM1_ESM.zip › Figure 1/1E/High merge Zoom.jpg]

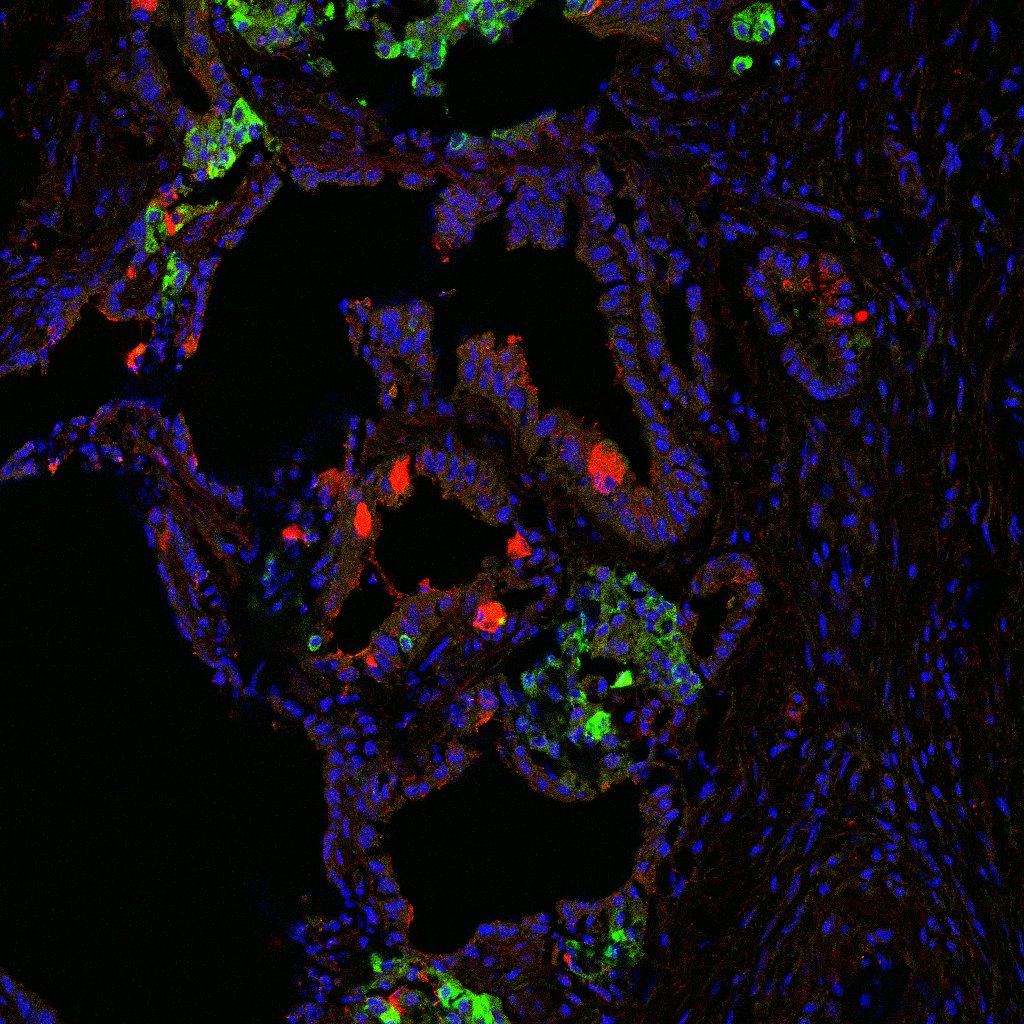

Supplement: Supplementary file 1 — Source Data Fig. 1 [file 44319_2024_104_MOESM1_ESM.zip › Figure 1/1E/High merge.jpg]

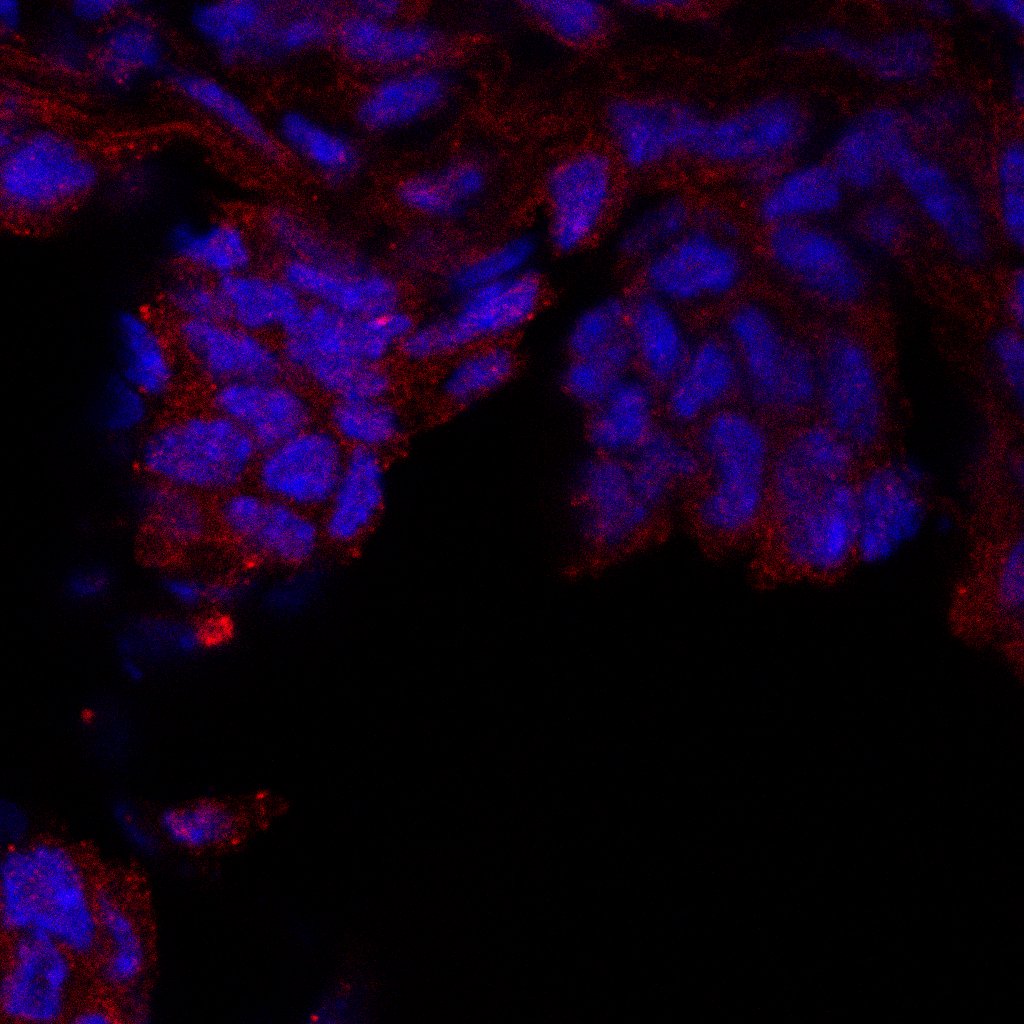

Supplement: Supplementary file 1 — Source Data Fig. 1 [file 44319_2024_104_MOESM1_ESM.zip › Figure 1/1E/High Nlgn2 Zoom.jpg]

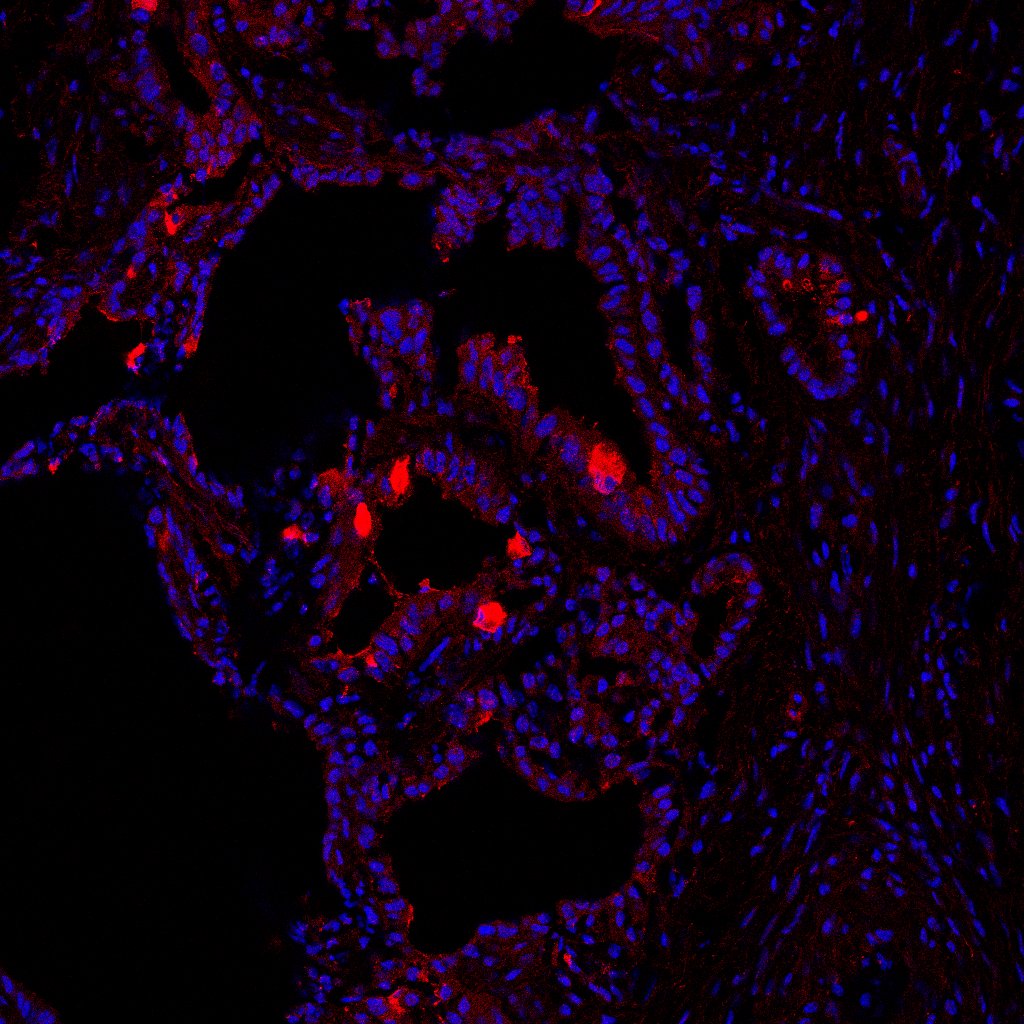

Supplement: Supplementary file 1 — Source Data Fig. 1 [file 44319_2024_104_MOESM1_ESM.zip › Figure 1/1E/High Nlgn2.jpg]

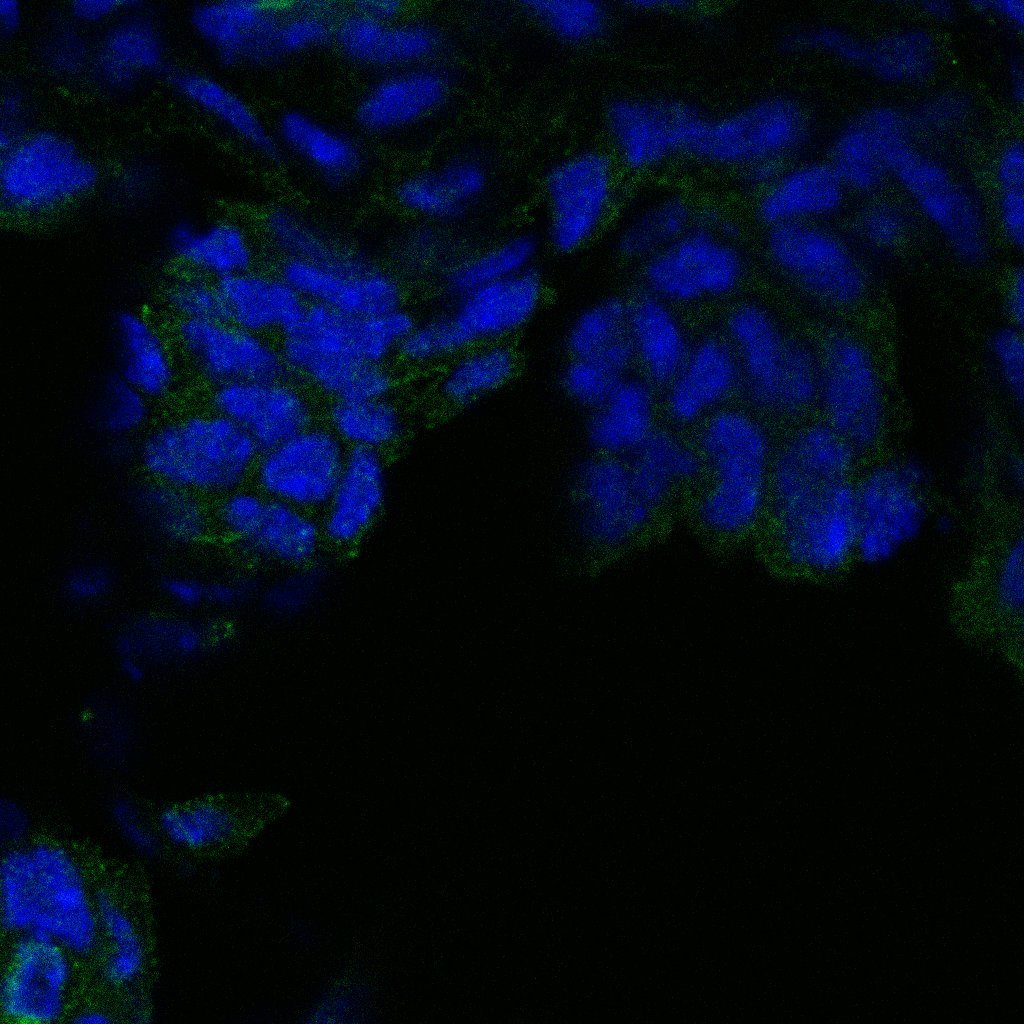

Supplement: Supplementary file 1 — Source Data Fig. 1 [file 44319_2024_104_MOESM1_ESM.zip › Figure 1/1E/High Zo1 Zoom.jpg]

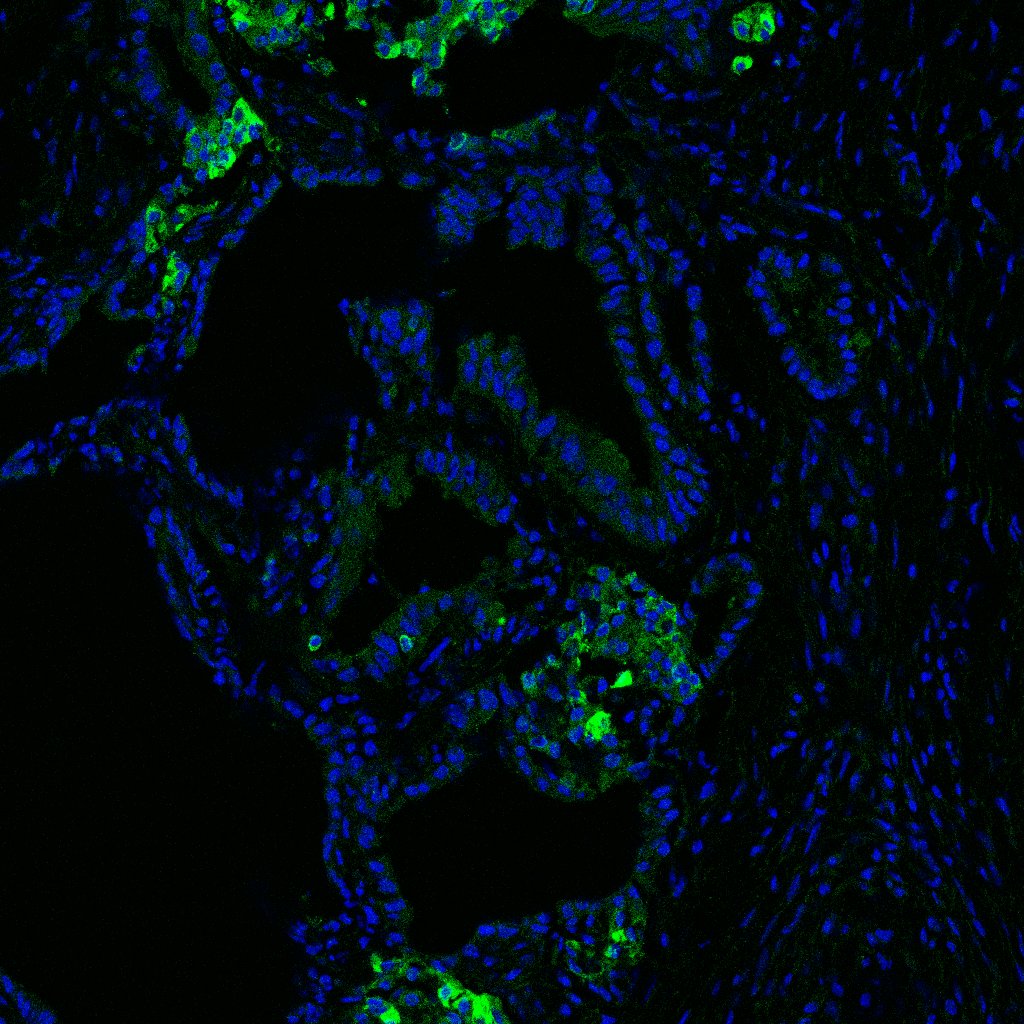

Supplement: Supplementary file 1 — Source Data Fig. 1 [file 44319_2024_104_MOESM1_ESM.zip › Figure 1/1E/High Zo1.jpg]

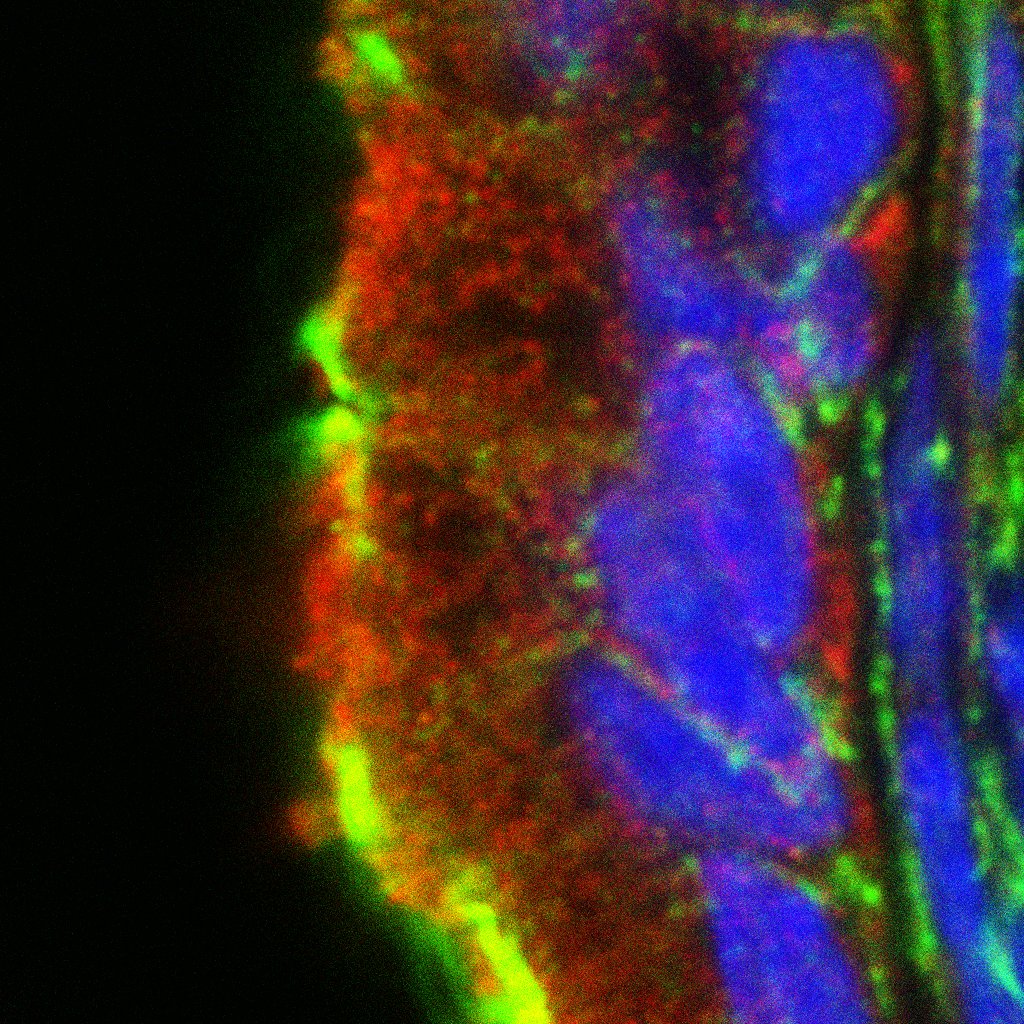

Supplement: Supplementary file 1 — Source Data Fig. 1 [file 44319_2024_104_MOESM1_ESM.zip › Figure 1/1E/Low merge Zoom.jpg]

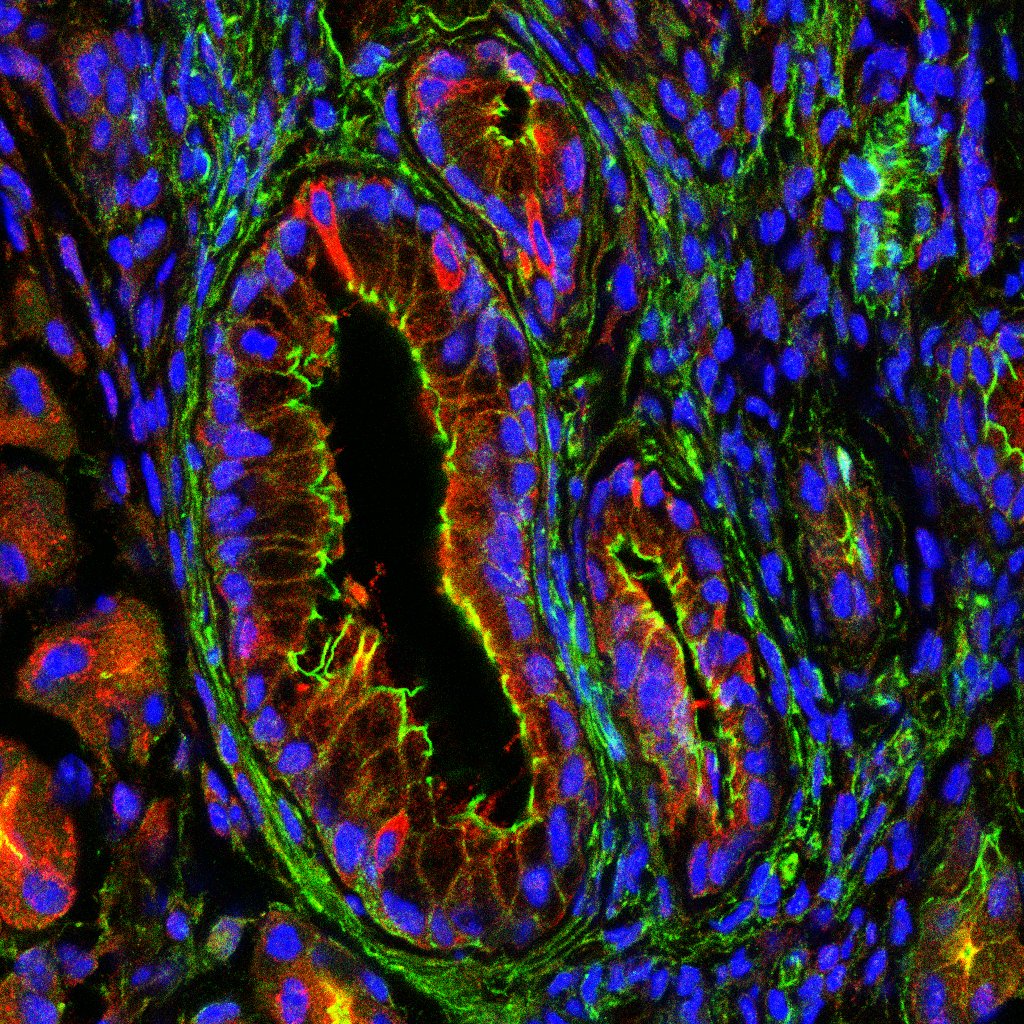

Supplement: Supplementary file 1 — Source Data Fig. 1 [file 44319_2024_104_MOESM1_ESM.zip › Figure 1/1E/Low merge.jpg]

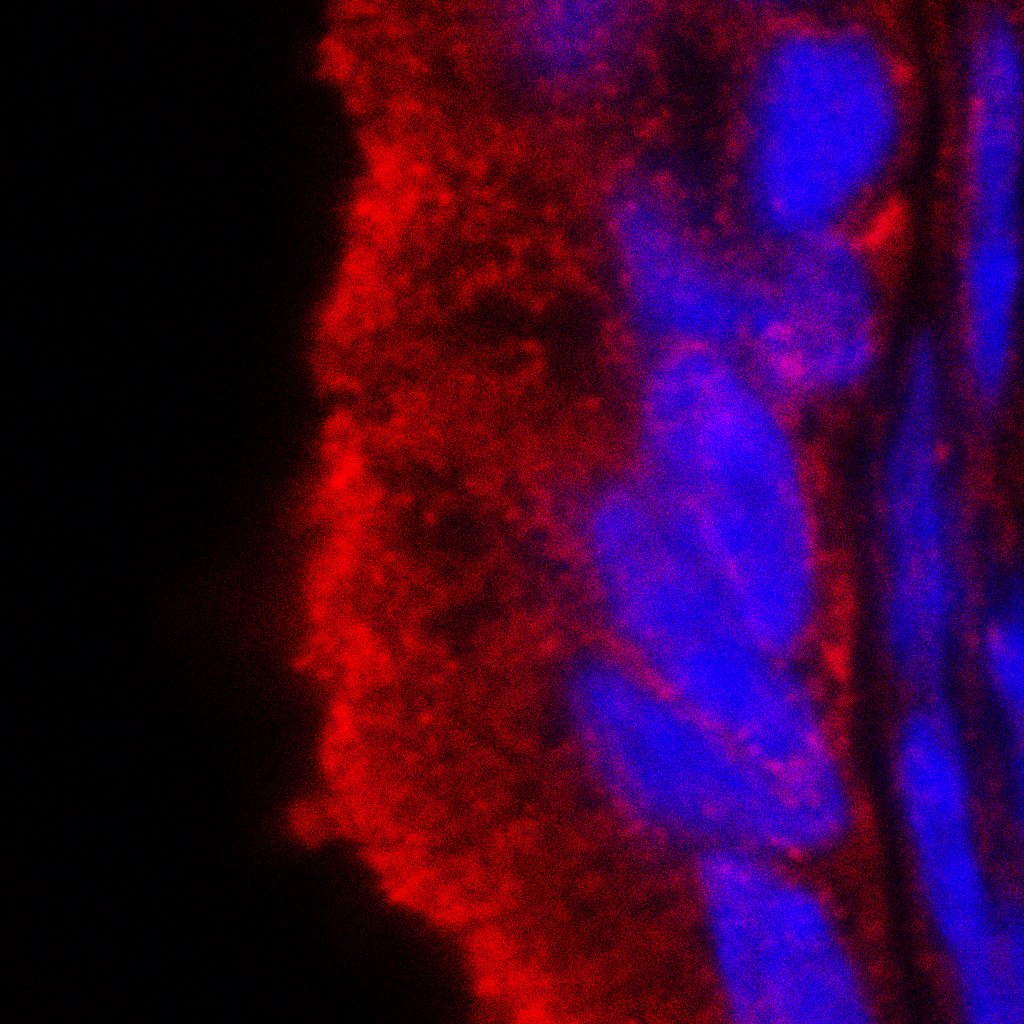

Supplement: Supplementary file 1 — Source Data Fig. 1 [file 44319_2024_104_MOESM1_ESM.zip › Figure 1/1E/Low Nlgn2 Zoom.jpg]

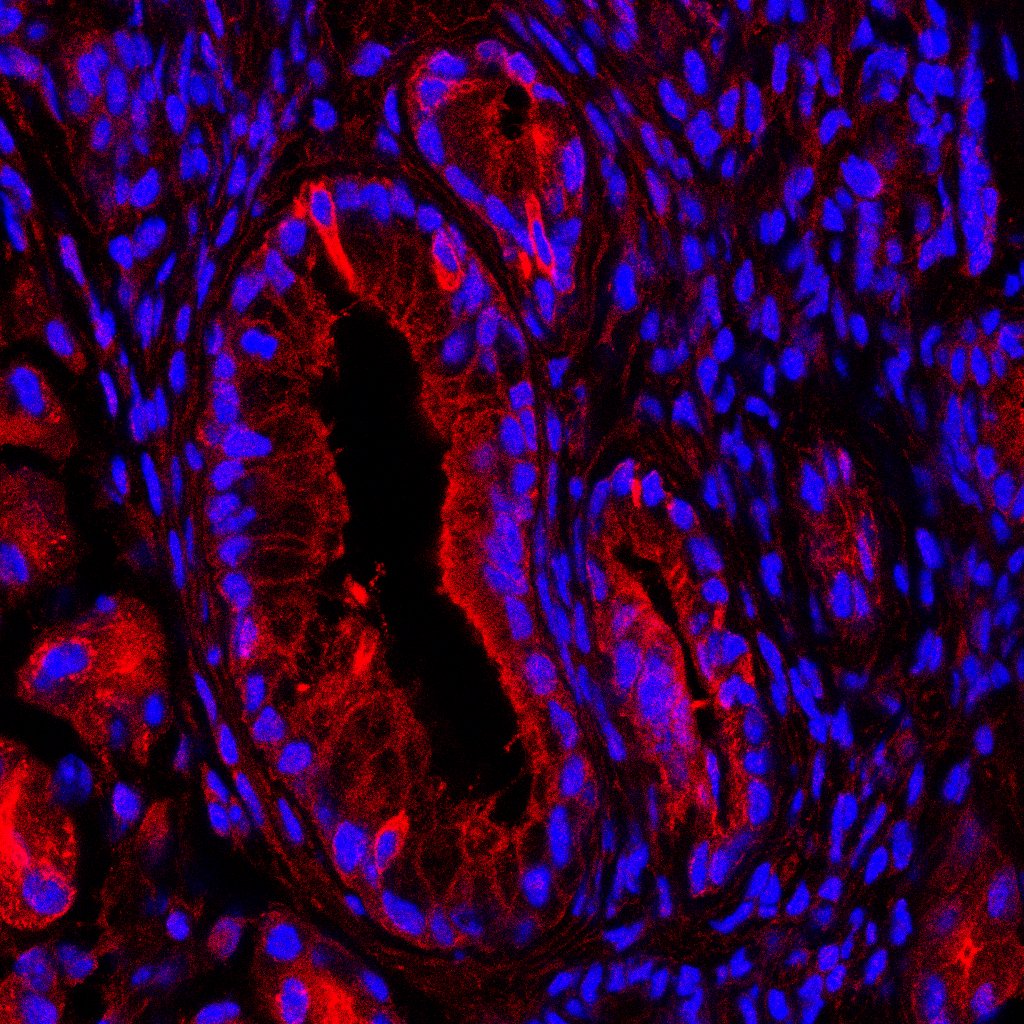

Supplement: Supplementary file 1 — Source Data Fig. 1 [file 44319_2024_104_MOESM1_ESM.zip › Figure 1/1E/Low Nlgn2.jpg]

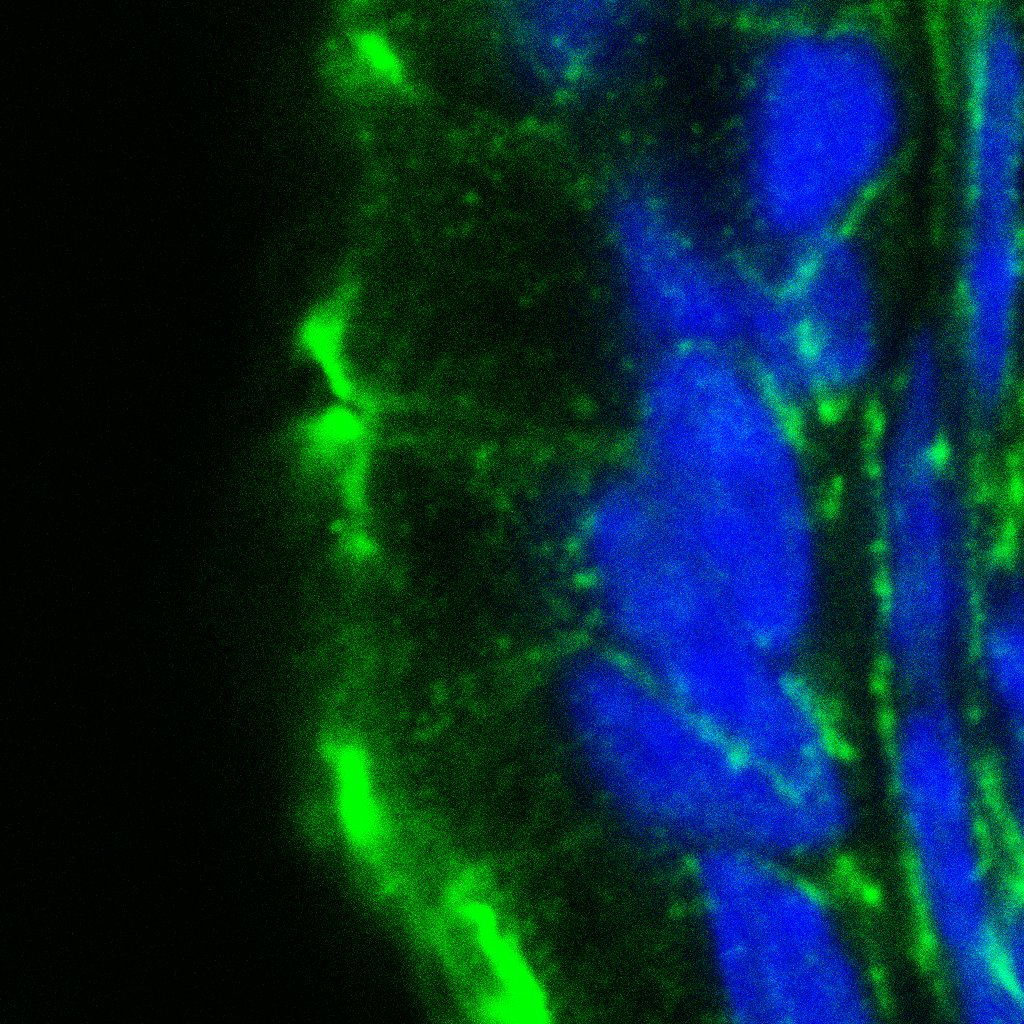

Supplement: Supplementary file 1 — Source Data Fig. 1 [file 44319_2024_104_MOESM1_ESM.zip › Figure 1/1E/Low Zo1 Zoom.jpg]

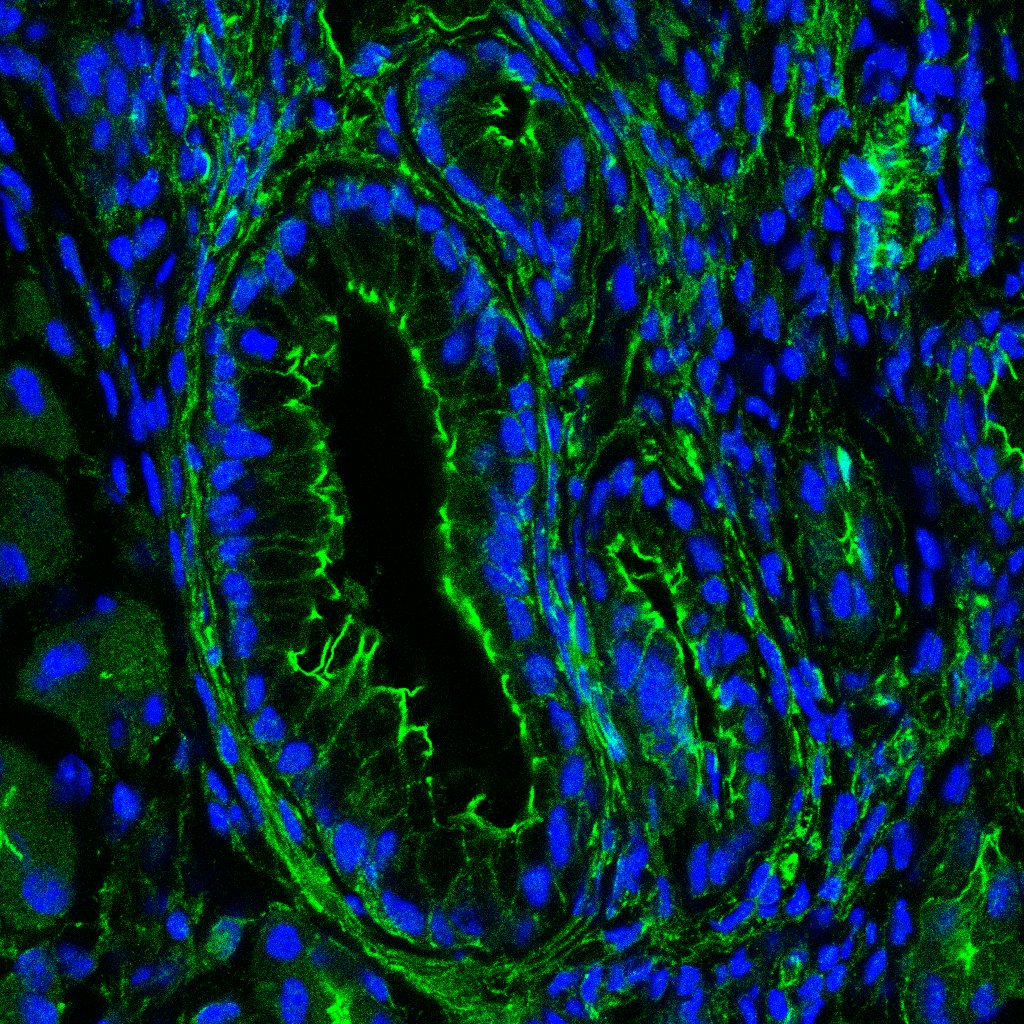

Supplement: Supplementary file 1 — Source Data Fig. 1 [file 44319_2024_104_MOESM1_ESM.zip › Figure 1/1E/Low Zo1.jpg]

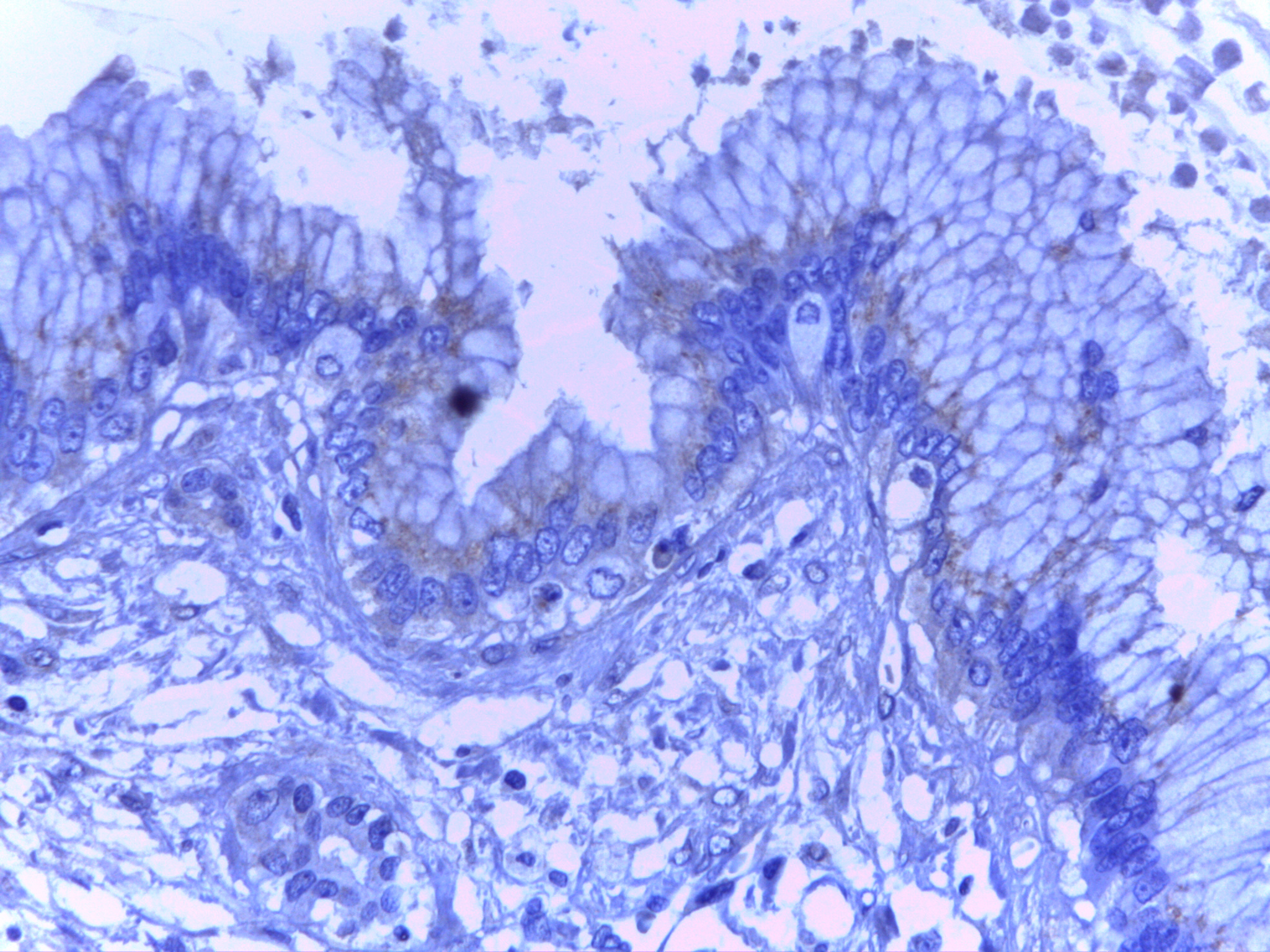

Supplement: Supplementary file 2 — Source Data Fig. 2 [file 44319_2024_104_MOESM2_ESM.zip › Figure 2/2B/PDAC Apical.jpg]

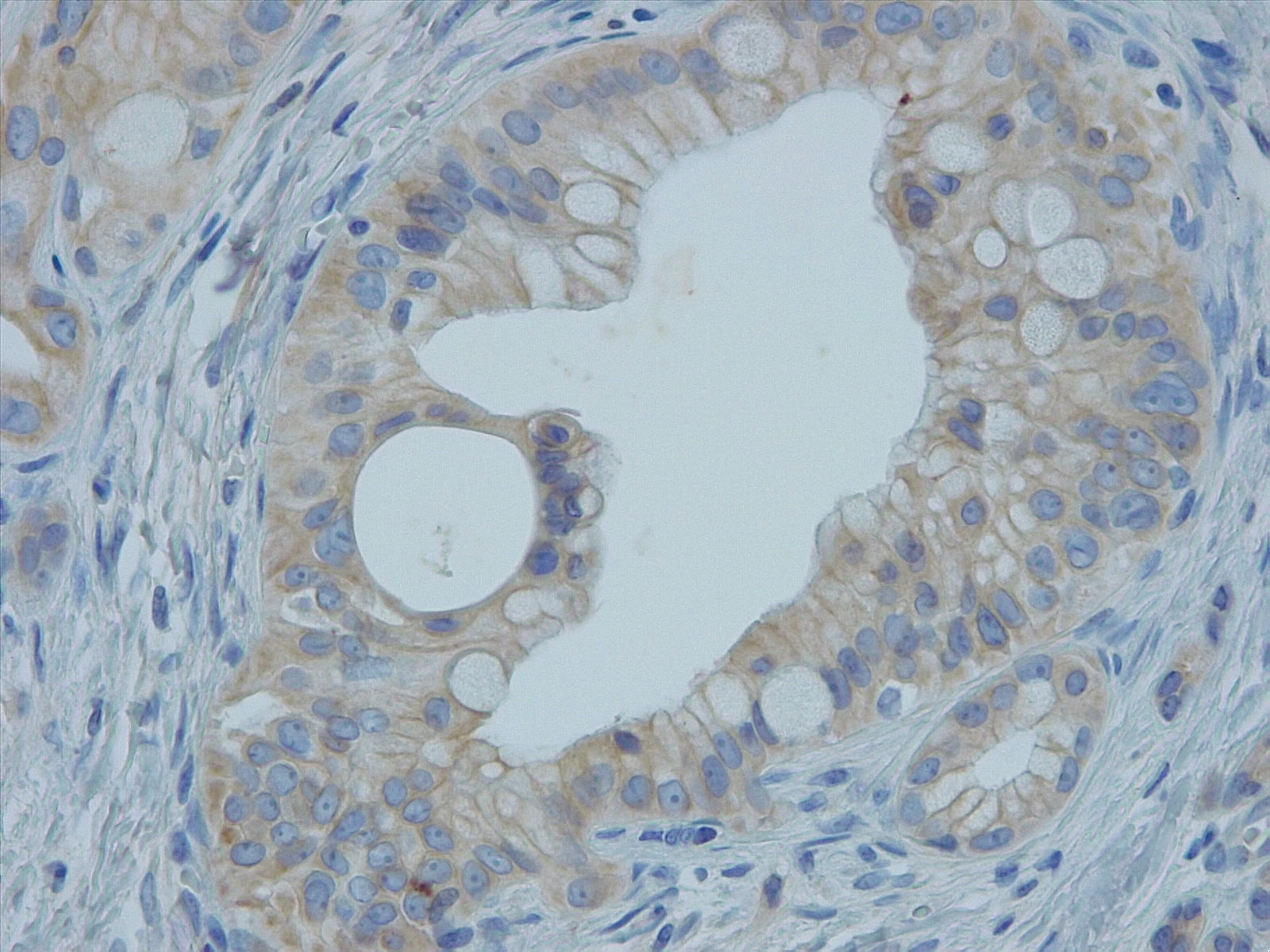

Supplement: Supplementary file 2 — Source Data Fig. 2 [file 44319_2024_104_MOESM2_ESM.zip › Figure 2/2B/PDAC Diffuse.jpg]

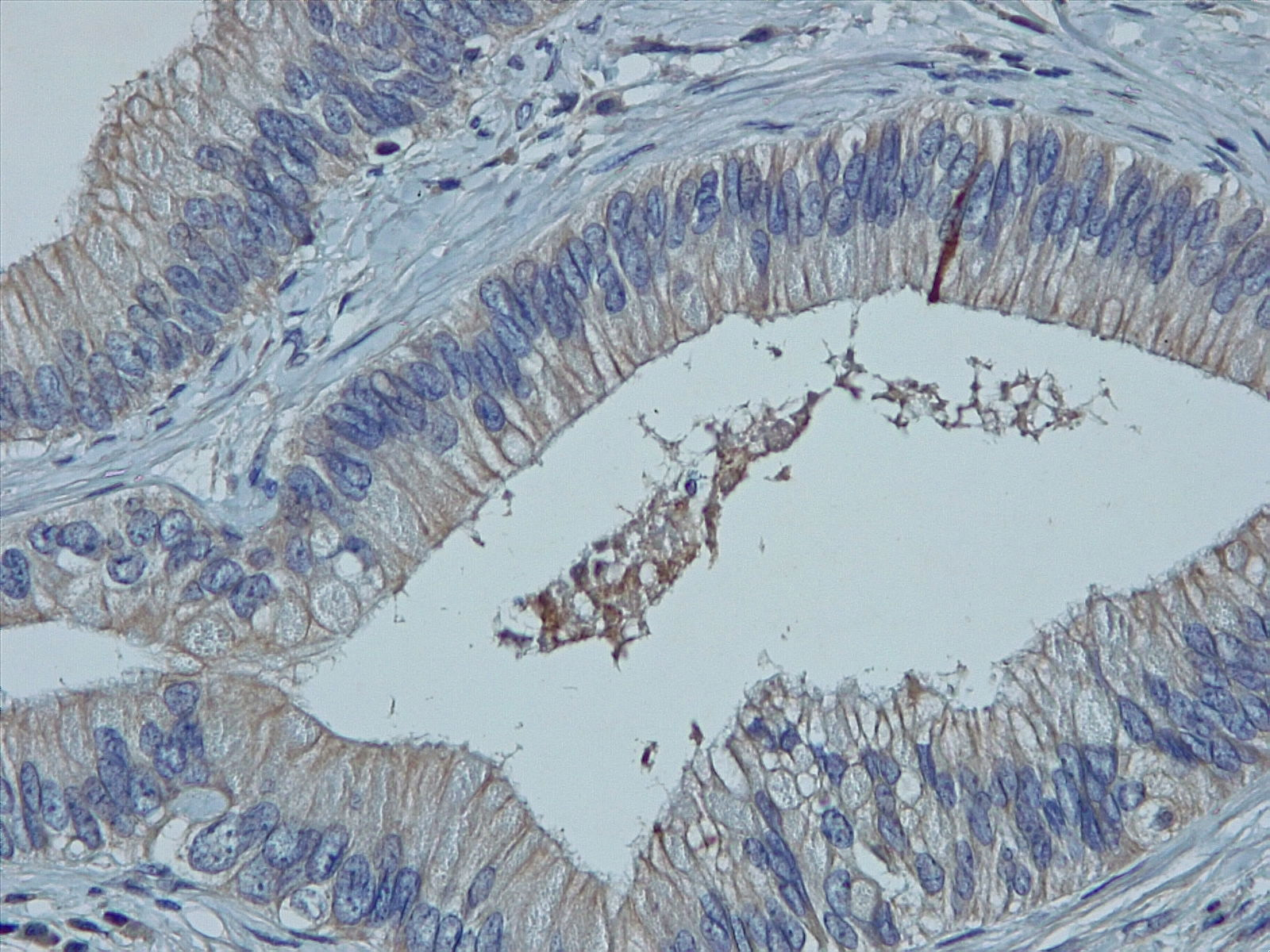

Supplement: Supplementary file 2 — Source Data Fig. 2 [file 44319_2024_104_MOESM2_ESM.zip › Figure 2/2B/PDAC Membr.jpg]

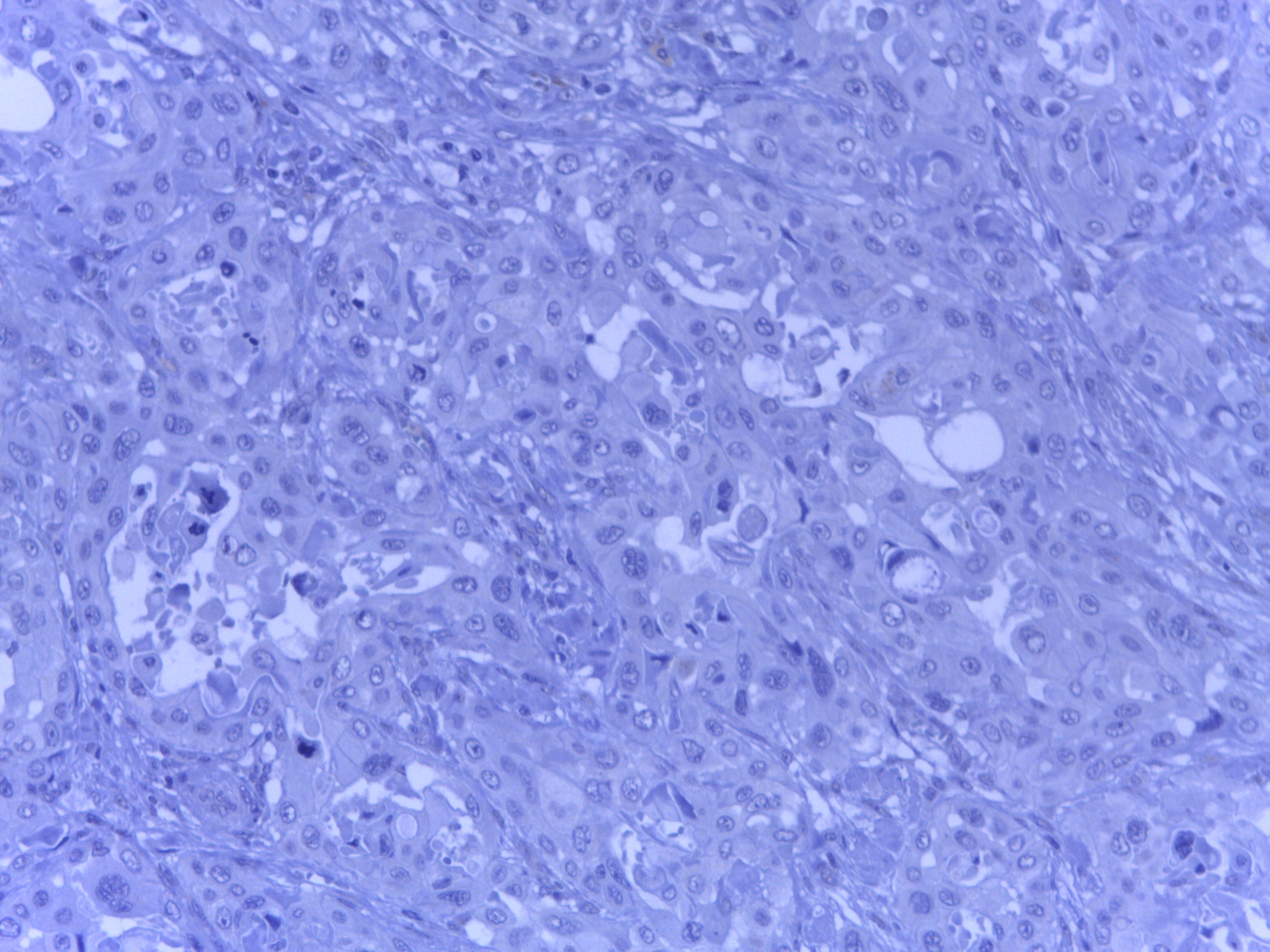

Supplement: Supplementary file 2 — Source Data Fig. 2 [file 44319_2024_104_MOESM2_ESM.zip › Figure 2/2B/PDAC Neg.jpg]

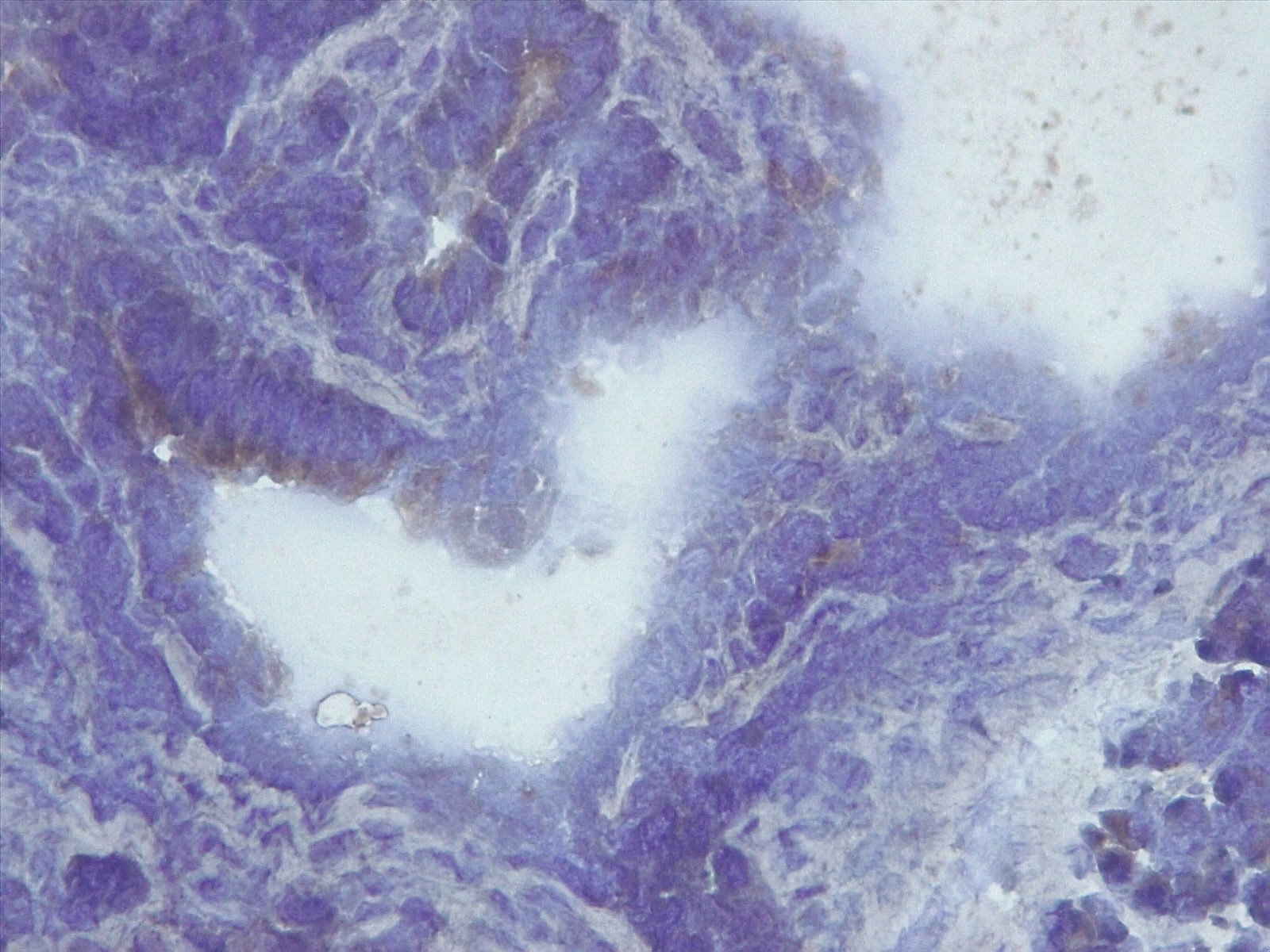

Supplement: Supplementary file 2 — Source Data Fig. 2 [file 44319_2024_104_MOESM2_ESM.zip › Figure 2/2B/Pdx1-KRas Zoom1.jpg]

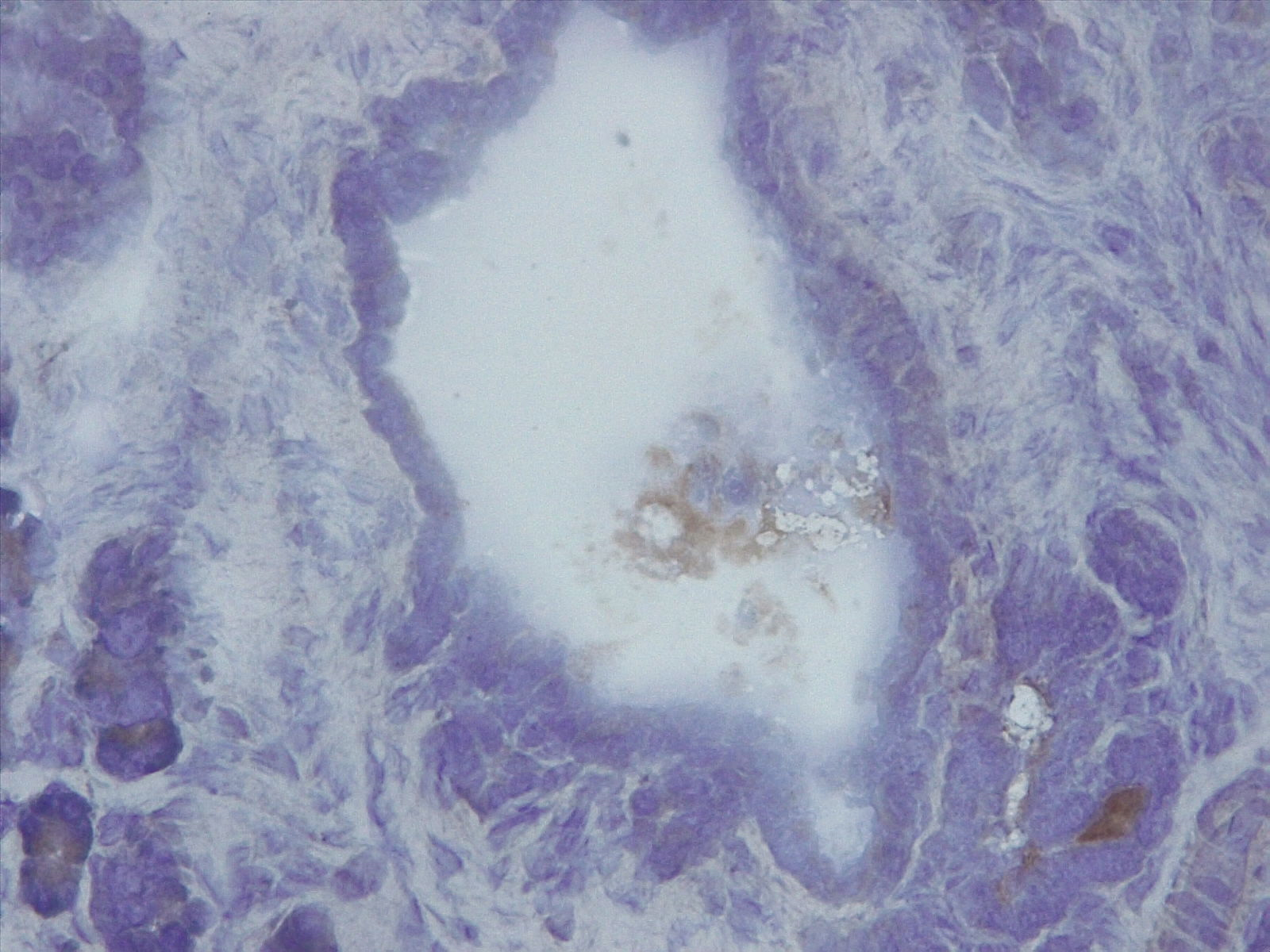

Supplement: Supplementary file 2 — Source Data Fig. 2 [file 44319_2024_104_MOESM2_ESM.zip › Figure 2/2B/Pdx1-KRas Zoom2.jpg]

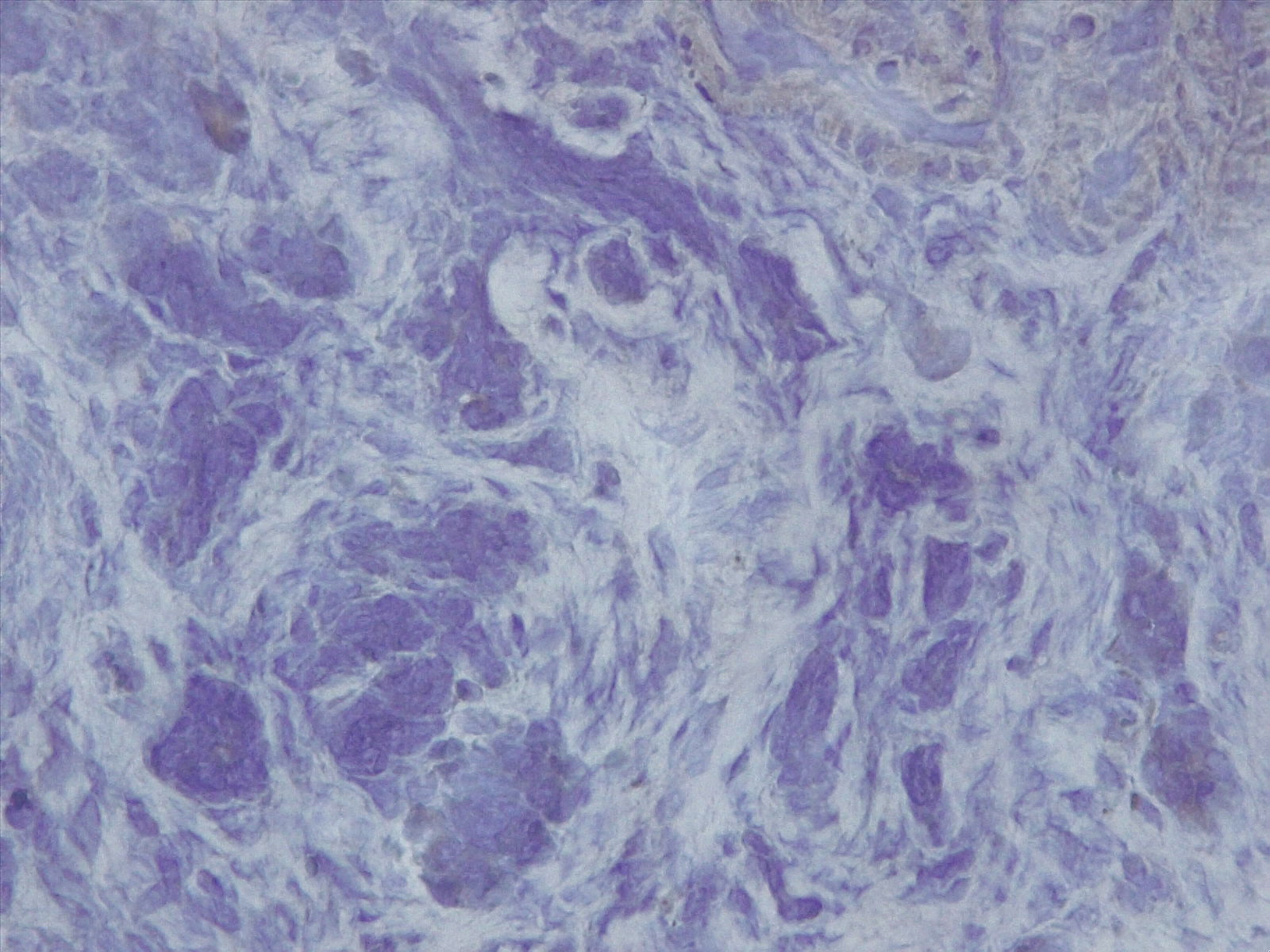

Supplement: Supplementary file 2 — Source Data Fig. 2 [file 44319_2024_104_MOESM2_ESM.zip › Figure 2/2B/Pdx1-KRas Zoom3.jpg]

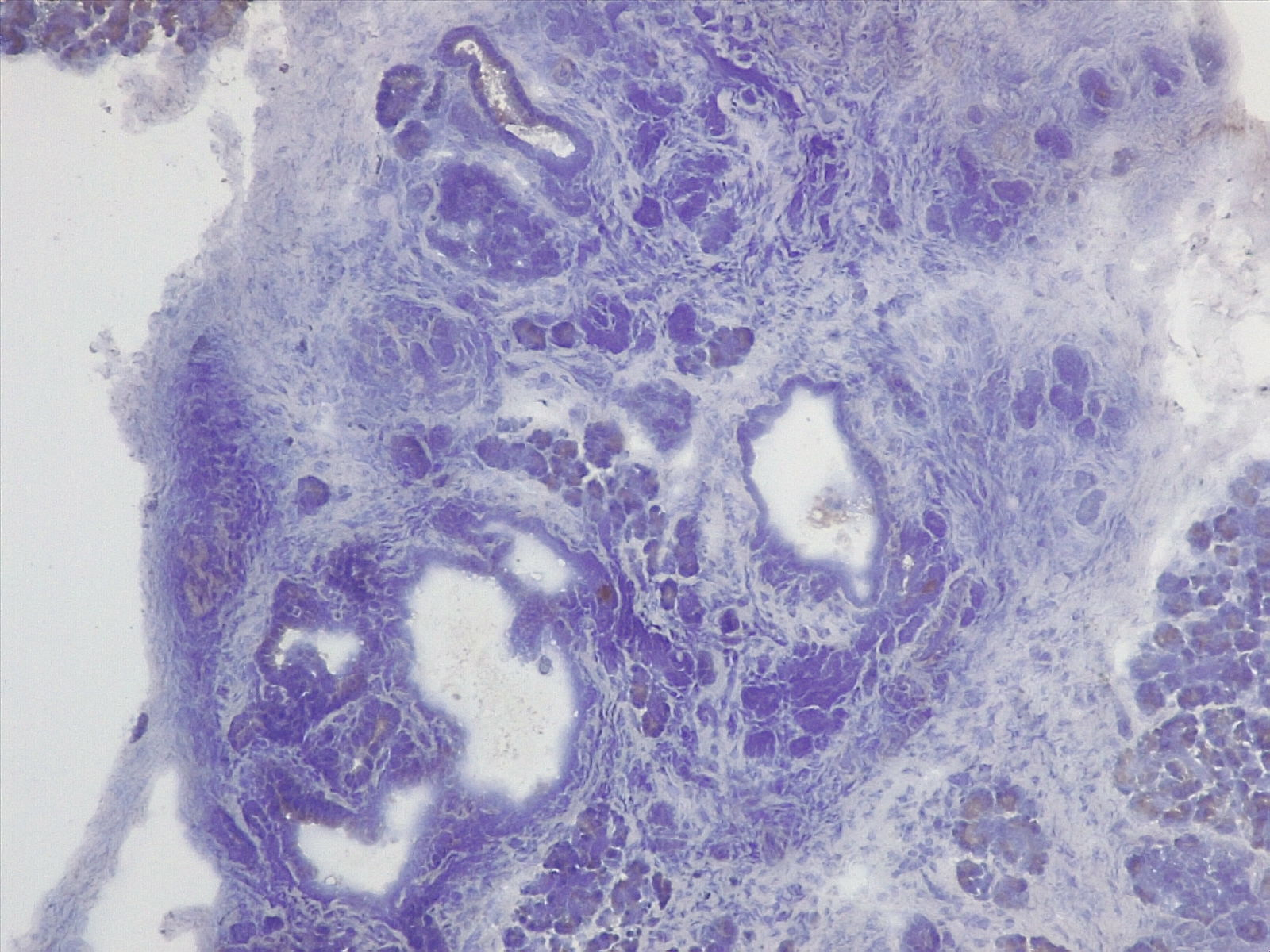

Supplement: Supplementary file 2 — Source Data Fig. 2 [file 44319_2024_104_MOESM2_ESM.zip › Figure 2/2B/Pdx1-KRas.jpg]

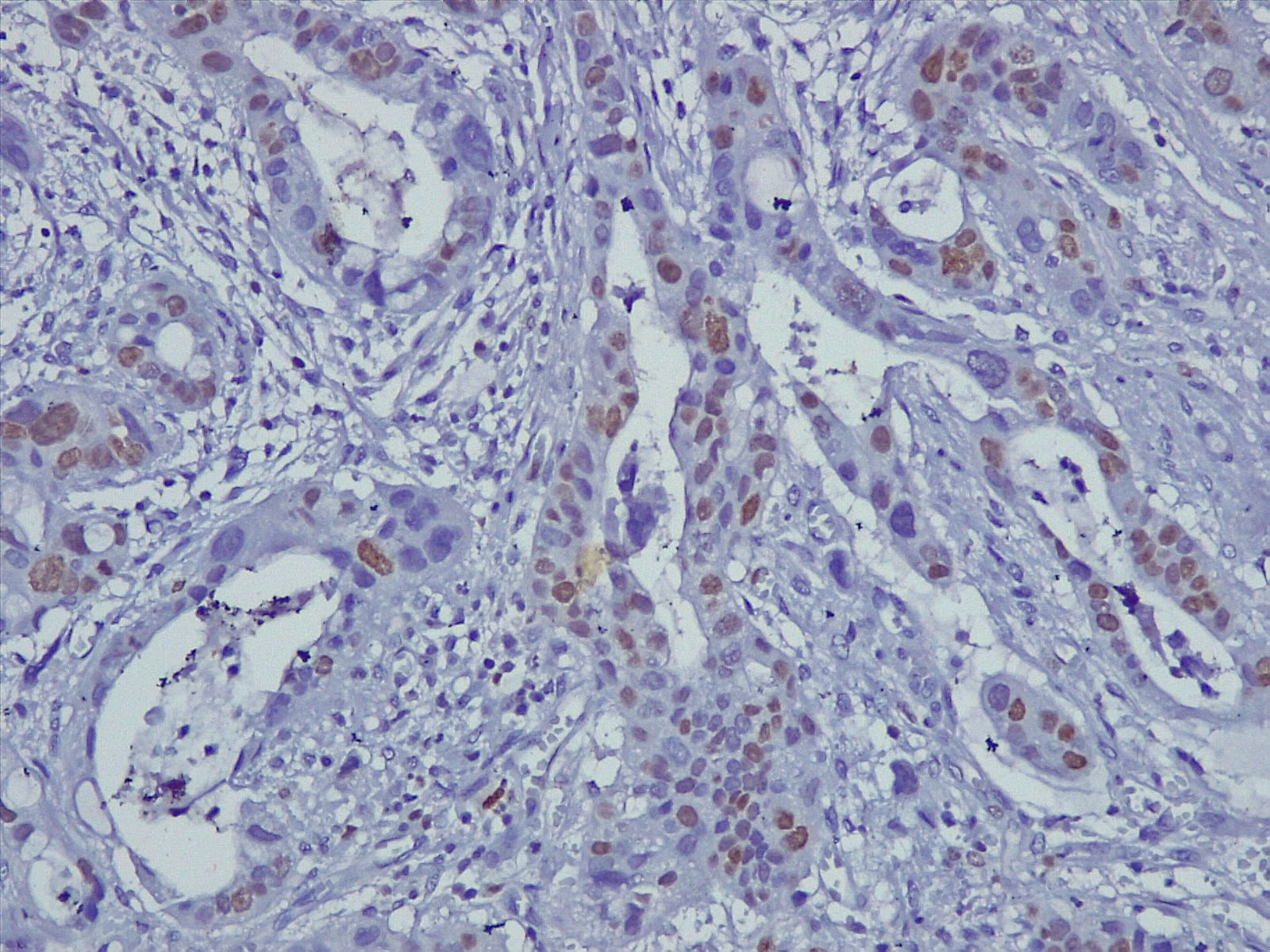

Supplement: Supplementary file 2 — Source Data Fig. 2 [file 44319_2024_104_MOESM2_ESM.zip › Figure 2/2C/KI67+.jpg]

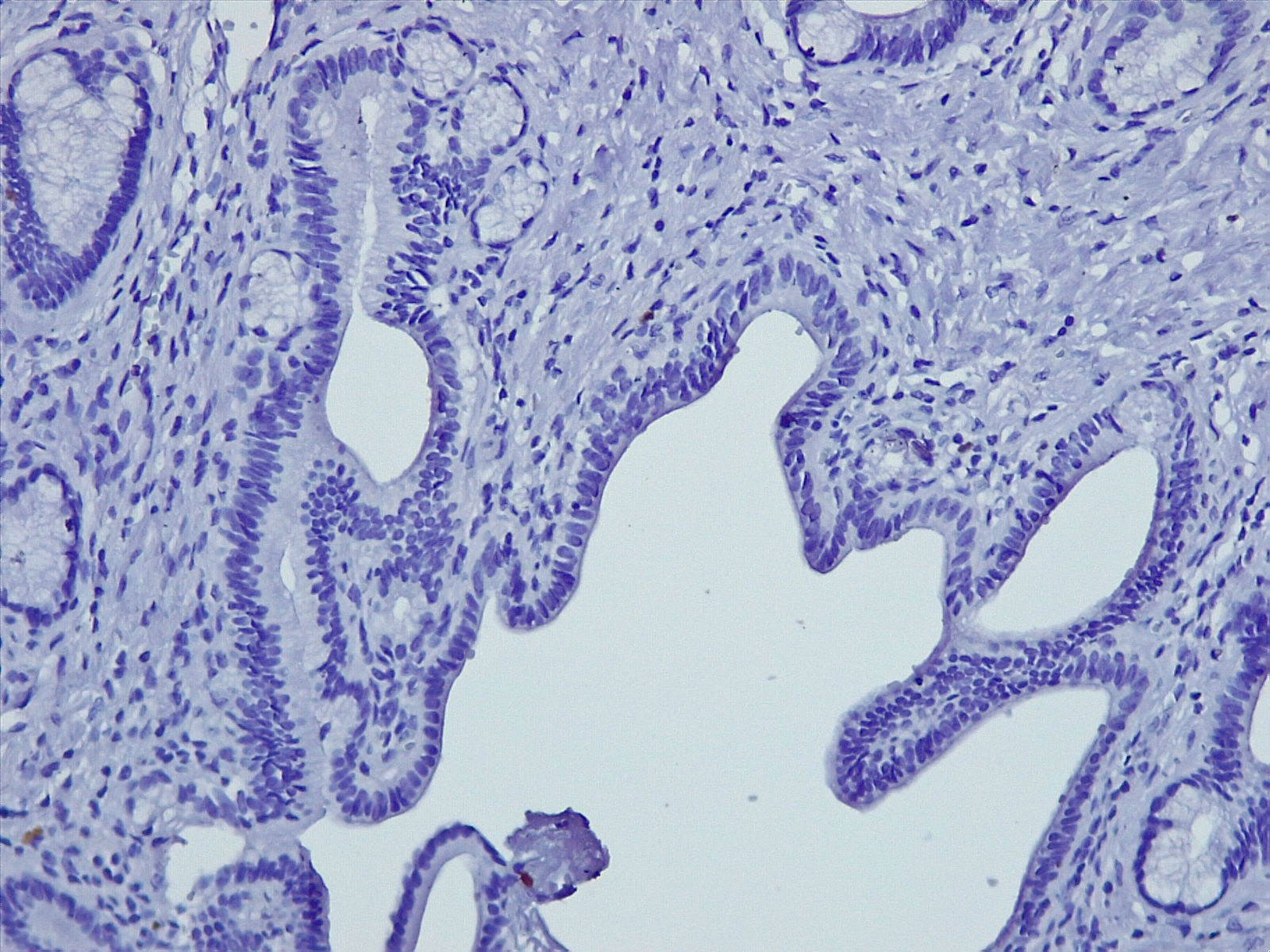

Supplement: Supplementary file 2 — Source Data Fig. 2 [file 44319_2024_104_MOESM2_ESM.zip › Figure 2/2C/KI67-.jpg]

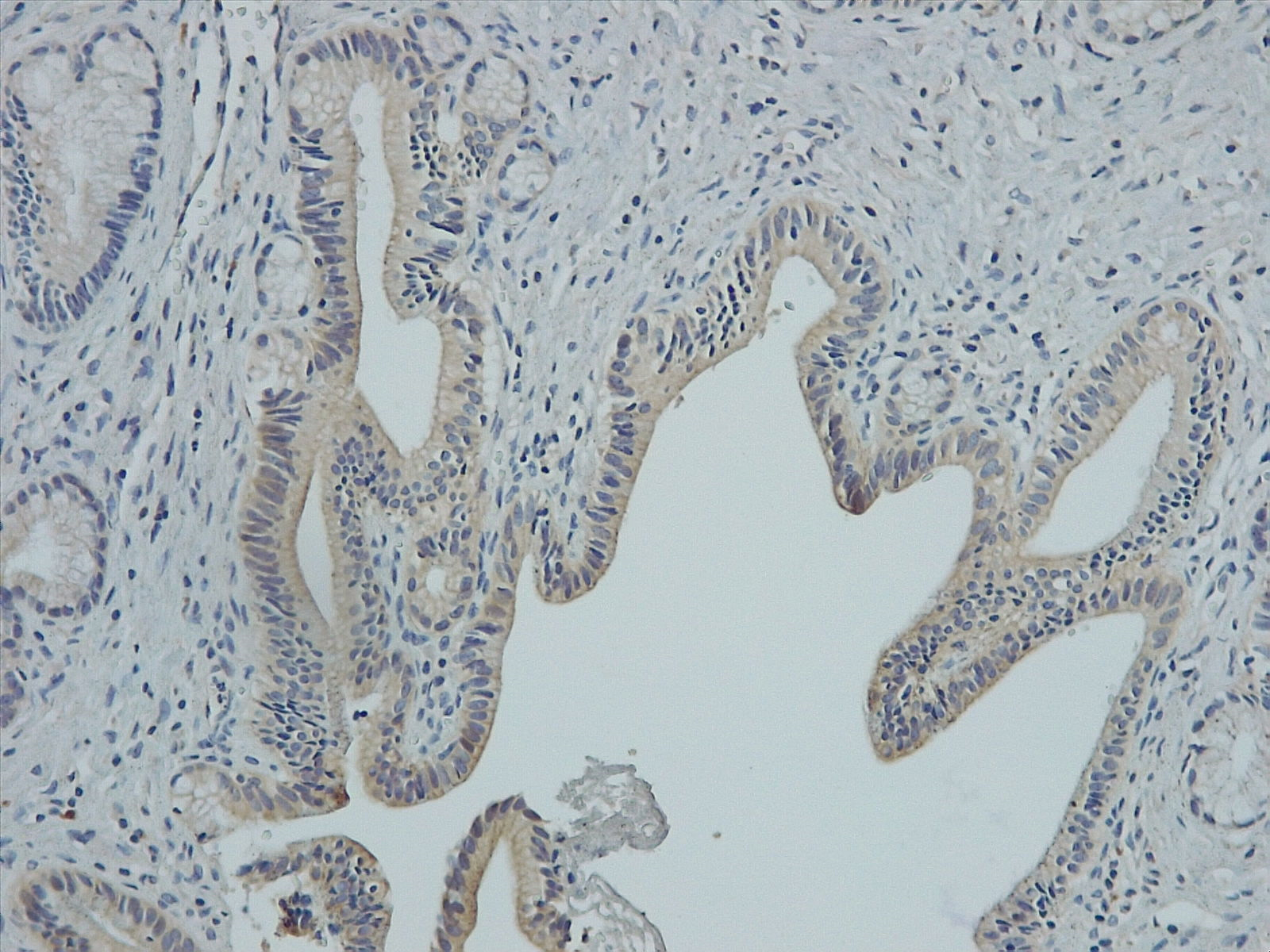

Supplement: Supplementary file 2 — Source Data Fig. 2 [file 44319_2024_104_MOESM2_ESM.zip › Figure 2/2C/N2+.jpg]

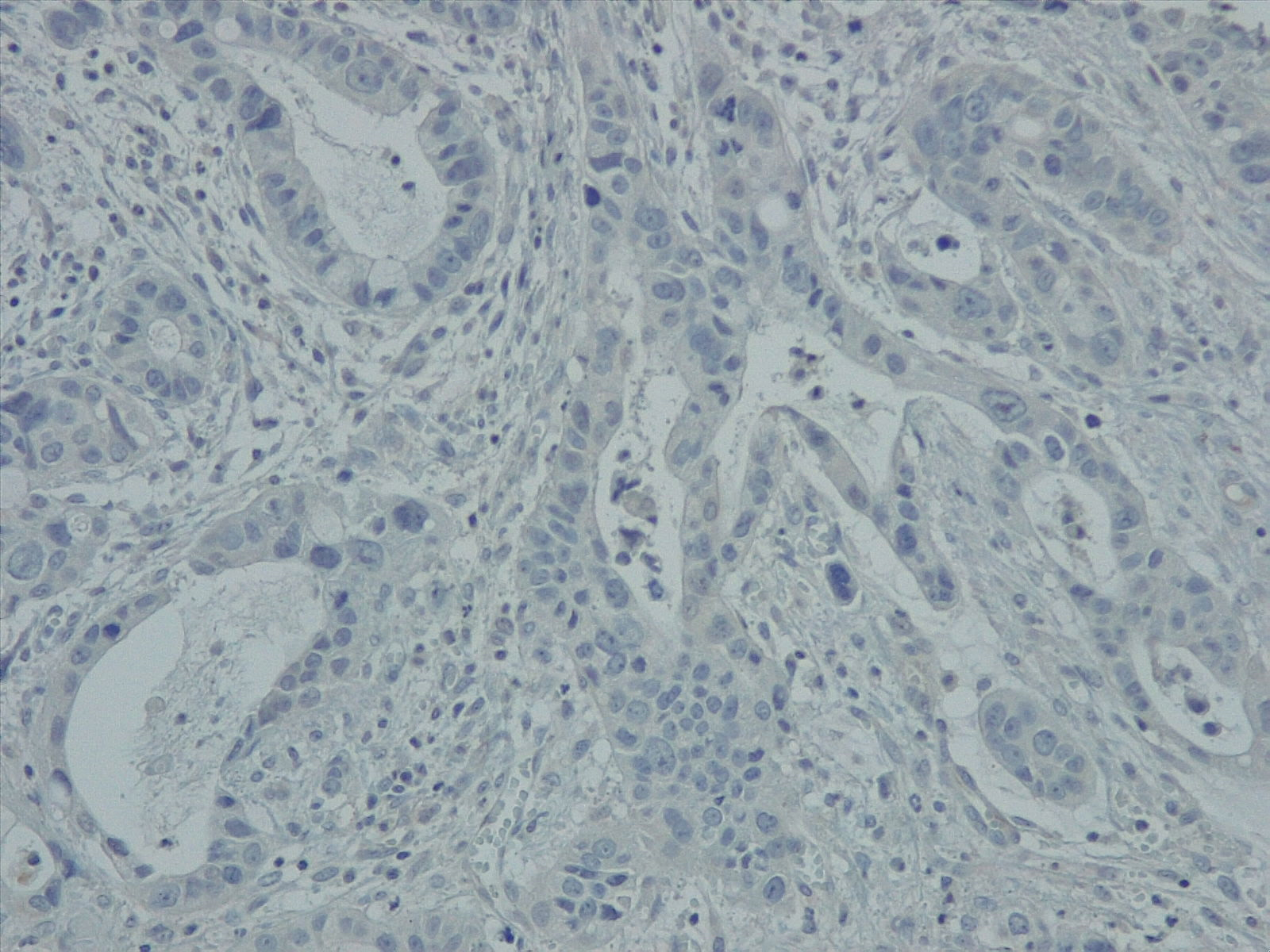

Supplement: Supplementary file 2 — Source Data Fig. 2 [file 44319_2024_104_MOESM2_ESM.zip › Figure 2/2C/N2-.jpg]

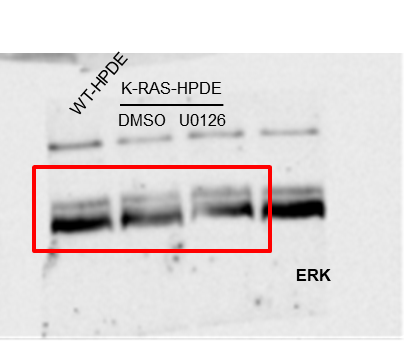

Supplement: Supplementary file 3 — Source Data Fig. 3 [file 44319_2024_104_MOESM3_ESM.zip › Figure 3/3A/WB ERK.tif]

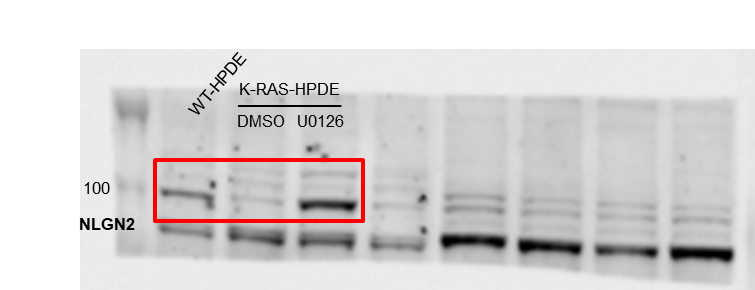

Supplement: Supplementary file 3 — Source Data Fig. 3 [file 44319_2024_104_MOESM3_ESM.zip › Figure 3/3A/WB NLGN2.tif]

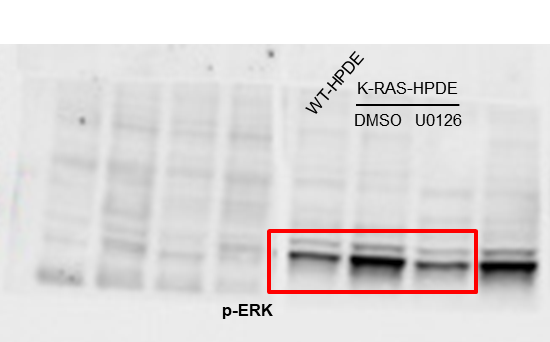

Supplement: Supplementary file 3 — Source Data Fig. 3 [file 44319_2024_104_MOESM3_ESM.zip › Figure 3/3A/WB PERK.tif]

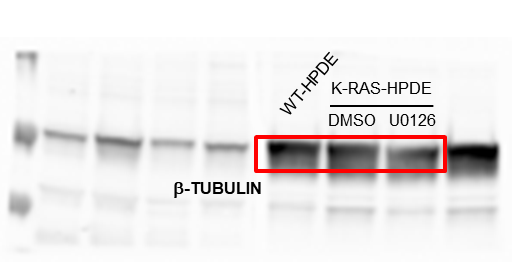

Supplement: Supplementary file 3 — Source Data Fig. 3 [file 44319_2024_104_MOESM3_ESM.zip › Figure 3/3A/WB TUBULIN.tif]

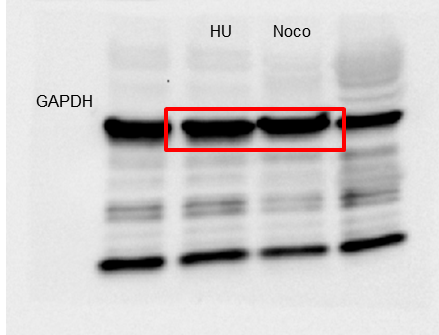

Supplement: Supplementary file 3 — Source Data Fig. 3 [file 44319_2024_104_MOESM3_ESM.zip › Figure 3/3B/WB GAPDH.tif]

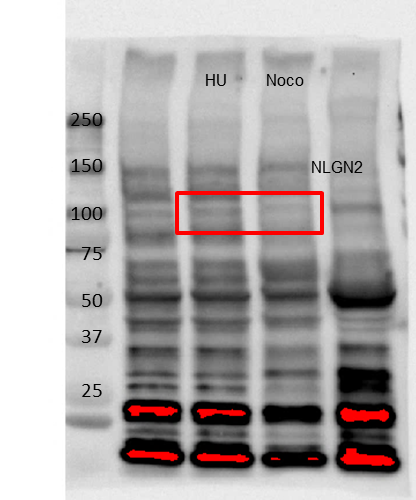

Supplement: Supplementary file 3 — Source Data Fig. 3 [file 44319_2024_104_MOESM3_ESM.zip › Figure 3/3B/WB NLGN2.tif]

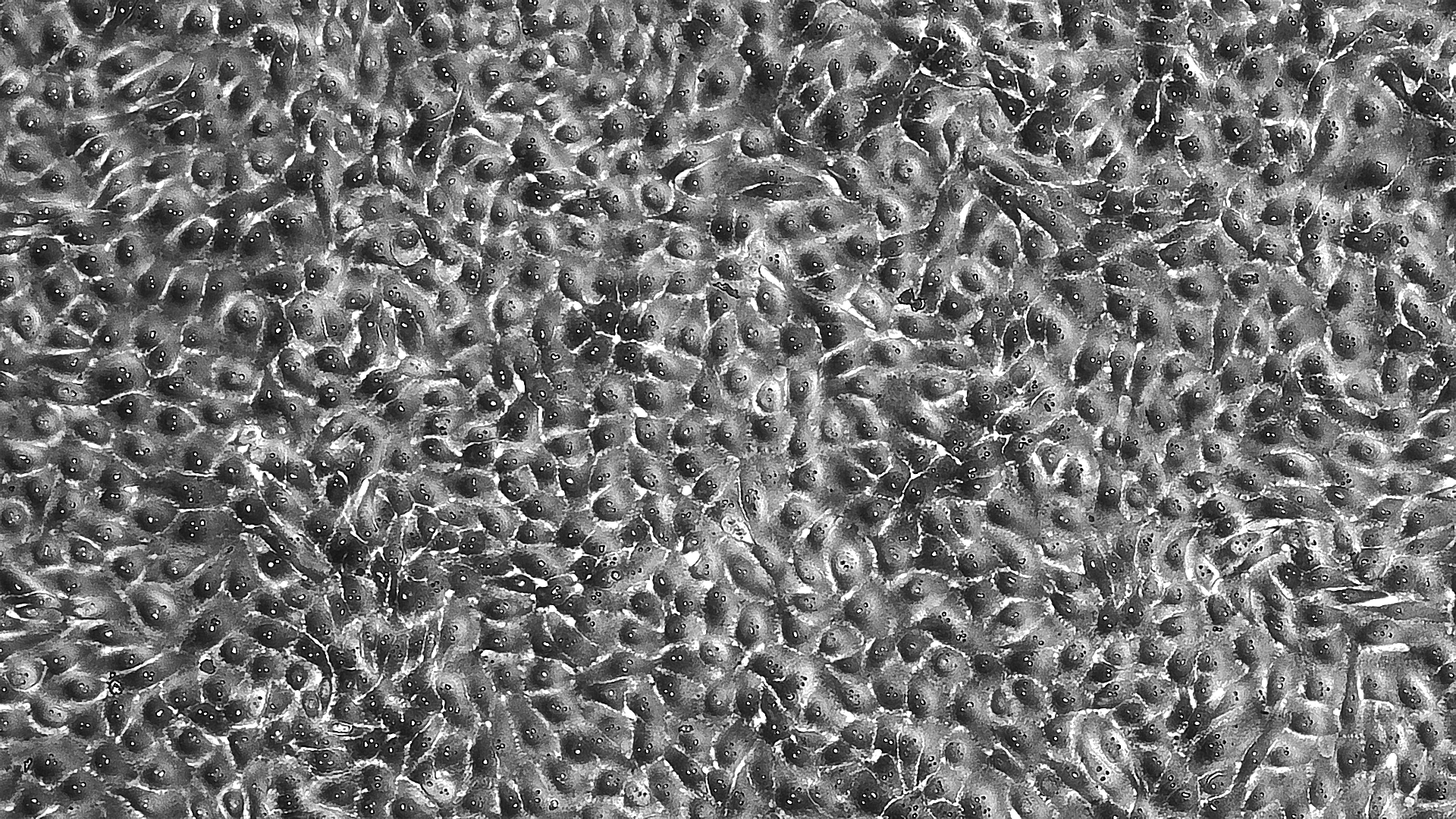

Supplement: Supplementary file 4 — Source Data Fig. 4 [file 44319_2024_104_MOESM4_ESM.zip › Figure 4/4A/Ctrl early zoom.tiff]

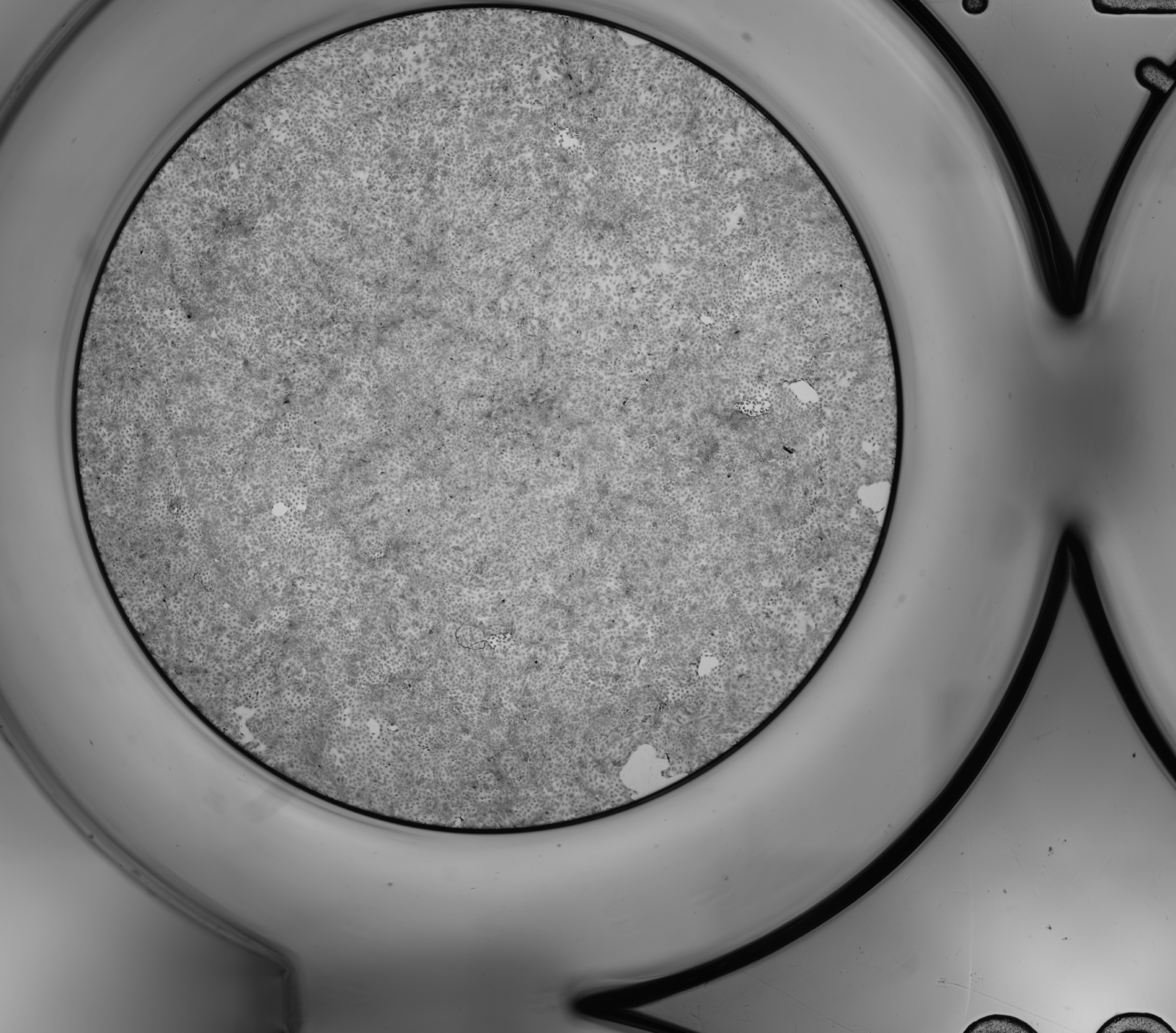

Supplement: Supplementary file 4 — Source Data Fig. 4 [file 44319_2024_104_MOESM4_ESM.zip › Figure 4/4A/Ctrl early.tif]

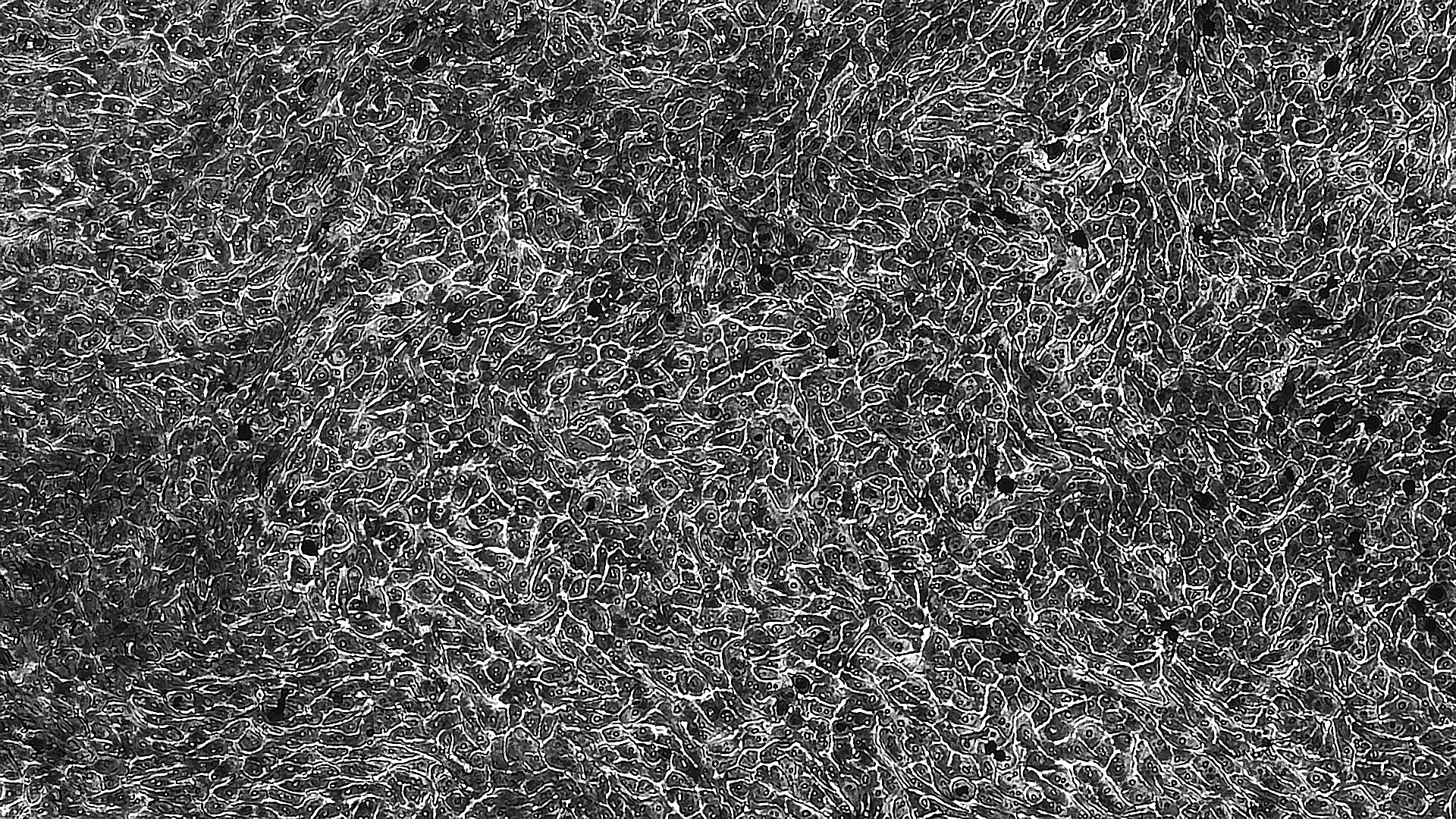

Supplement: Supplementary file 4 — Source Data Fig. 4 [file 44319_2024_104_MOESM4_ESM.zip › Figure 4/4A/Ctrl mature zoom.tiff]

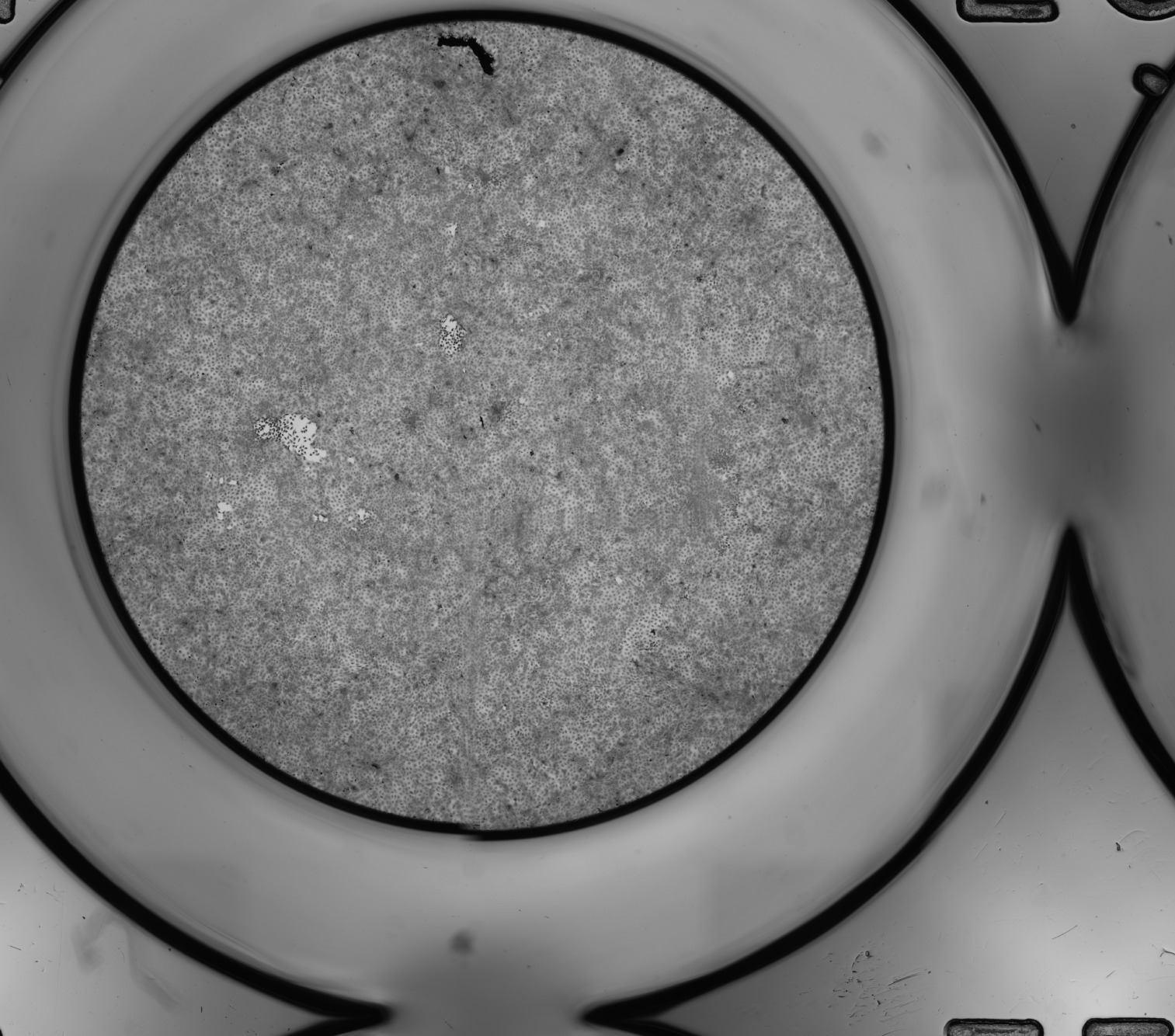

Supplement: Supplementary file 4 — Source Data Fig. 4 [file 44319_2024_104_MOESM4_ESM.zip › Figure 4/4A/Ctrl mature.tif]

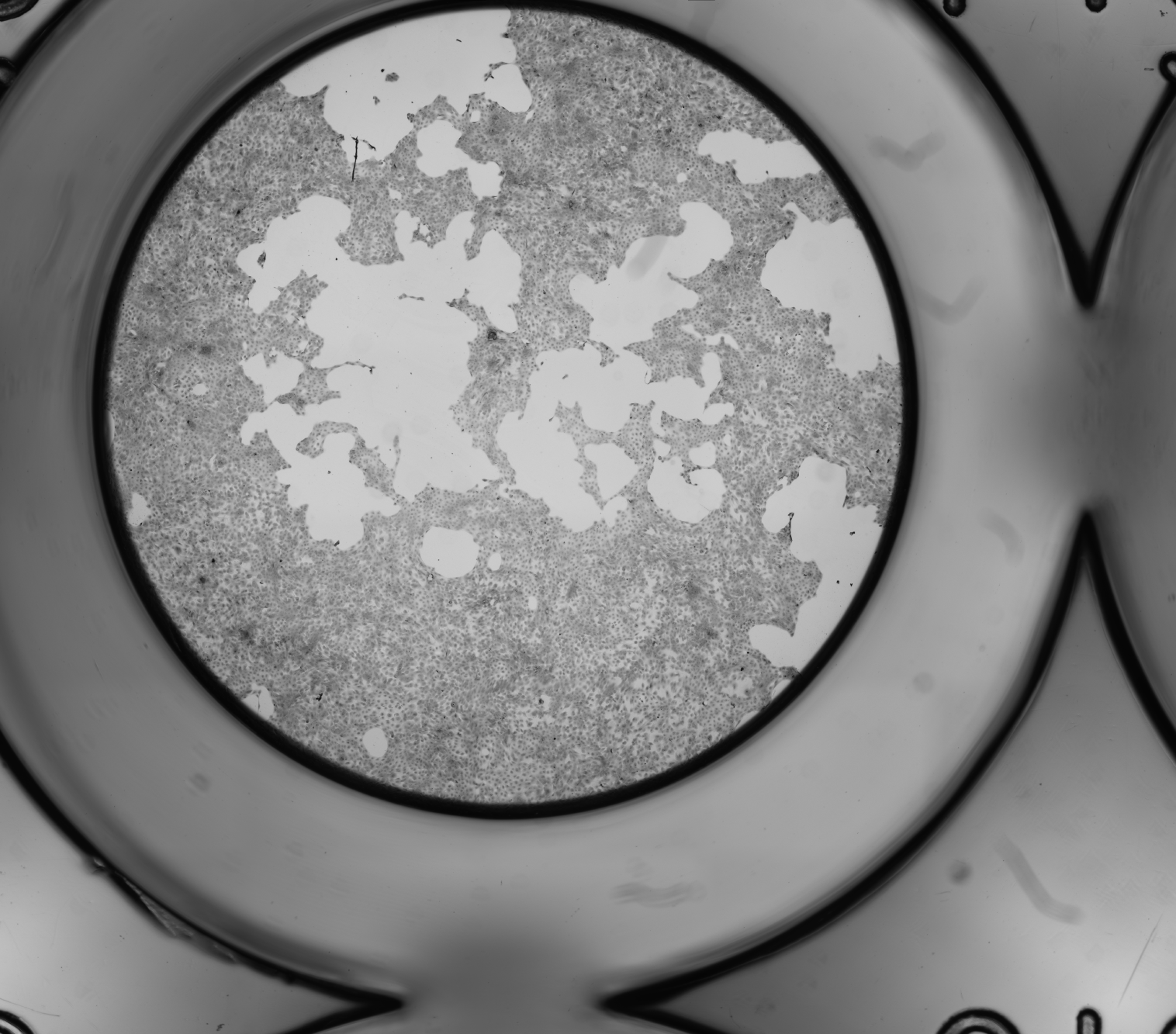

Supplement: Supplementary file 4 — Source Data Fig. 4 [file 44319_2024_104_MOESM4_ESM.zip › Figure 4/4A/Ctrl sub.tif]

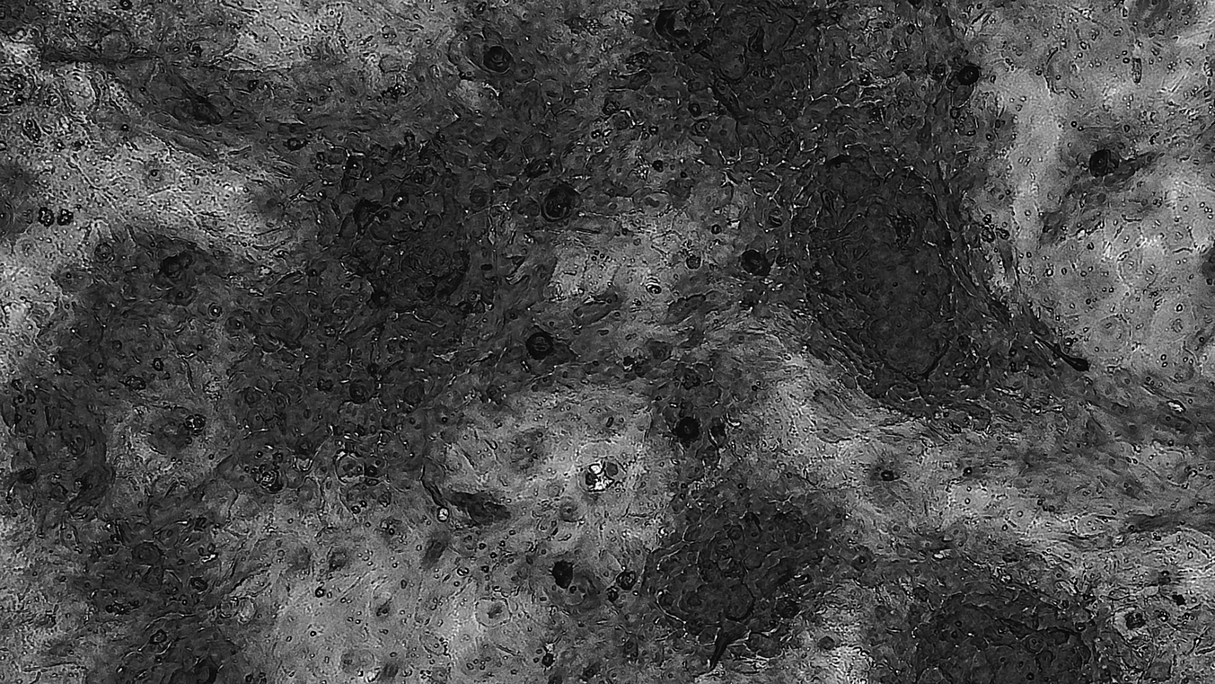

Supplement: Supplementary file 4 — Source Data Fig. 4 [file 44319_2024_104_MOESM4_ESM.zip › Figure 4/4A/sh mature zoom.tif]

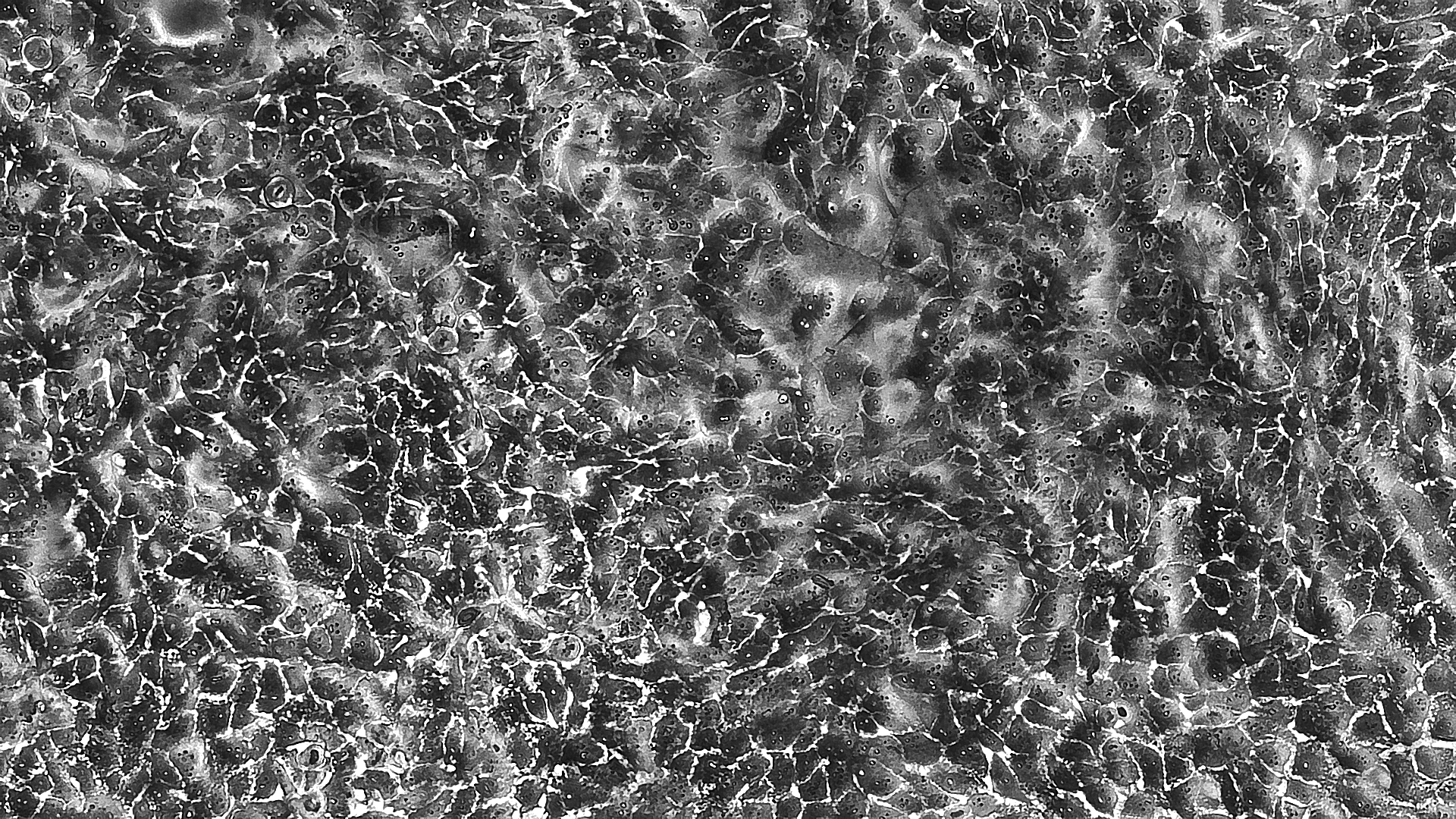

Supplement: Supplementary file 4 — Source Data Fig. 4 [file 44319_2024_104_MOESM4_ESM.zip › Figure 4/4A/shNLGN2 early zoom.tiff]

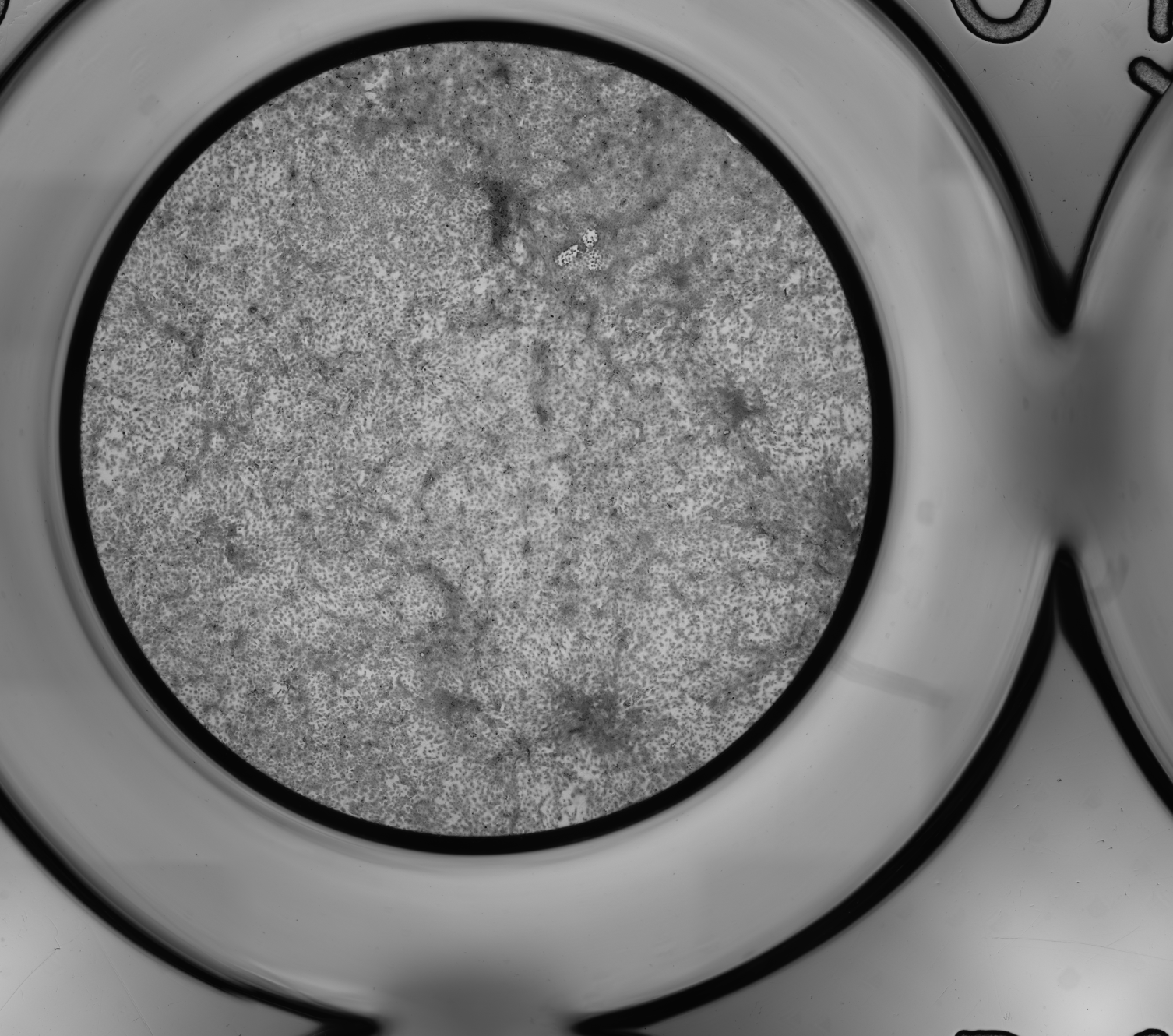

Supplement: Supplementary file 4 — Source Data Fig. 4 [file 44319_2024_104_MOESM4_ESM.zip › Figure 4/4A/shNLGN2 early.tif]

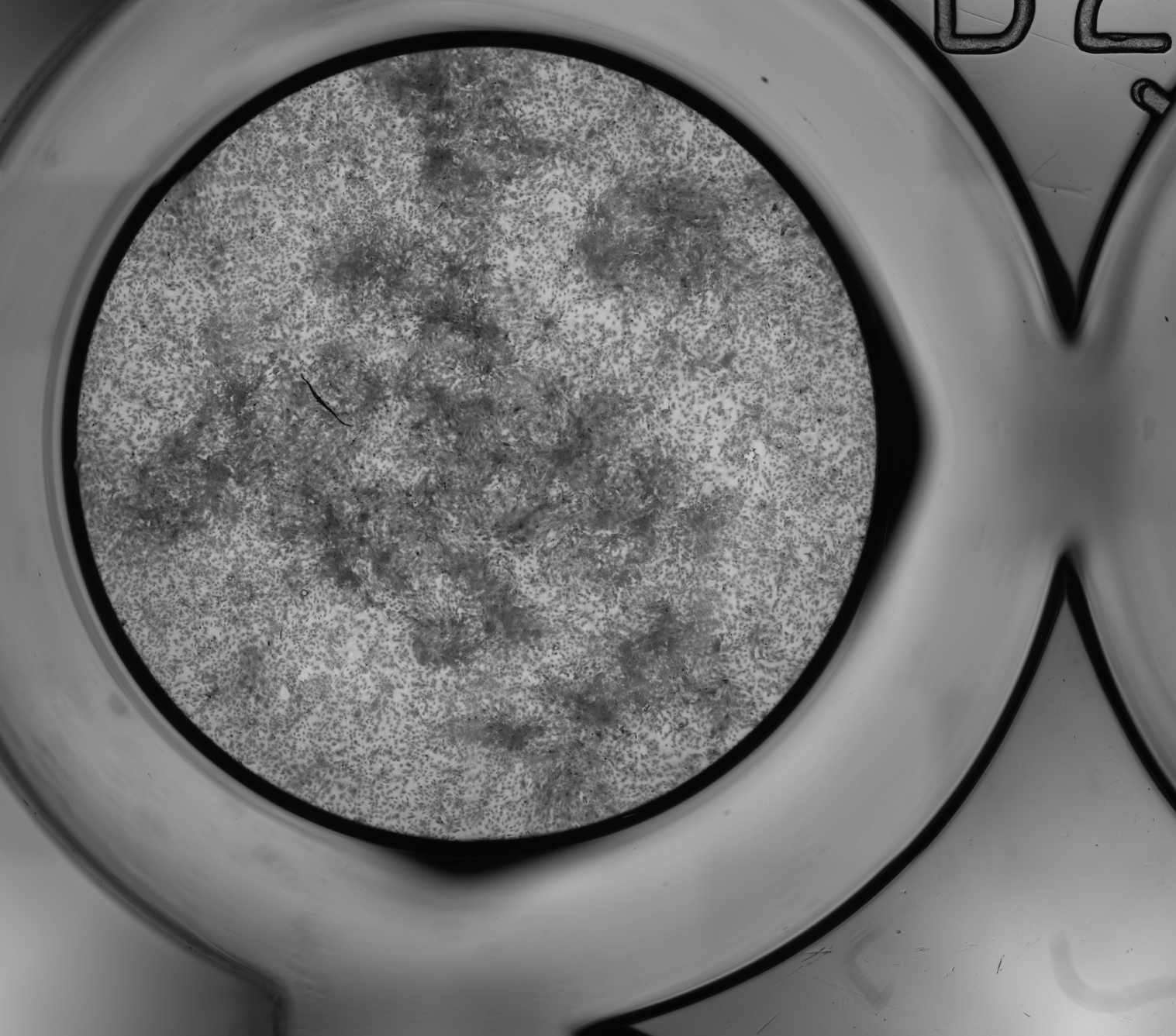

Supplement: Supplementary file 4 — Source Data Fig. 4 [file 44319_2024_104_MOESM4_ESM.zip › Figure 4/4A/shNLGN2 Mature.tif]

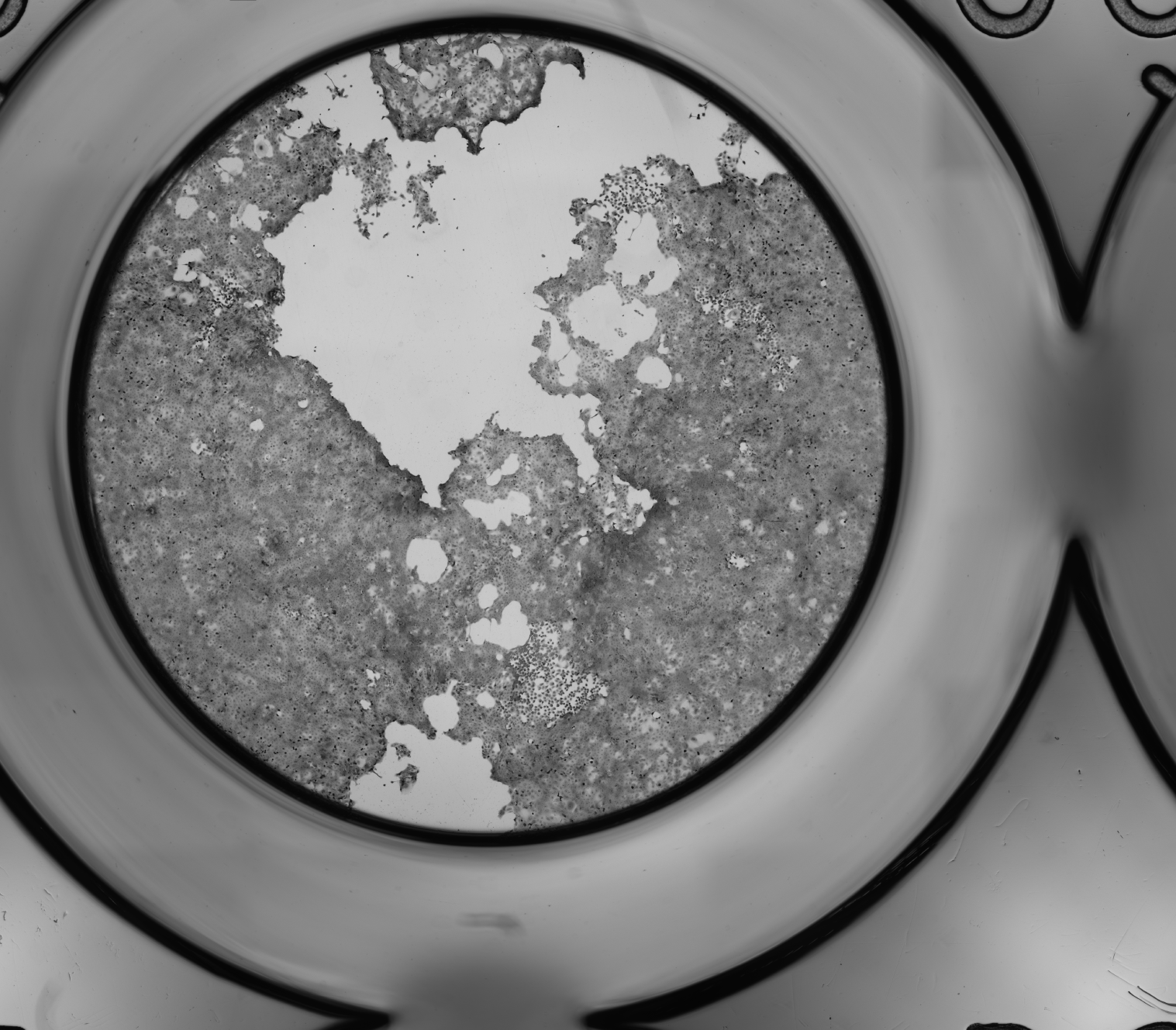

Supplement: Supplementary file 4 — Source Data Fig. 4 [file 44319_2024_104_MOESM4_ESM.zip › Figure 4/4A/shNLGN2 sub.tif]

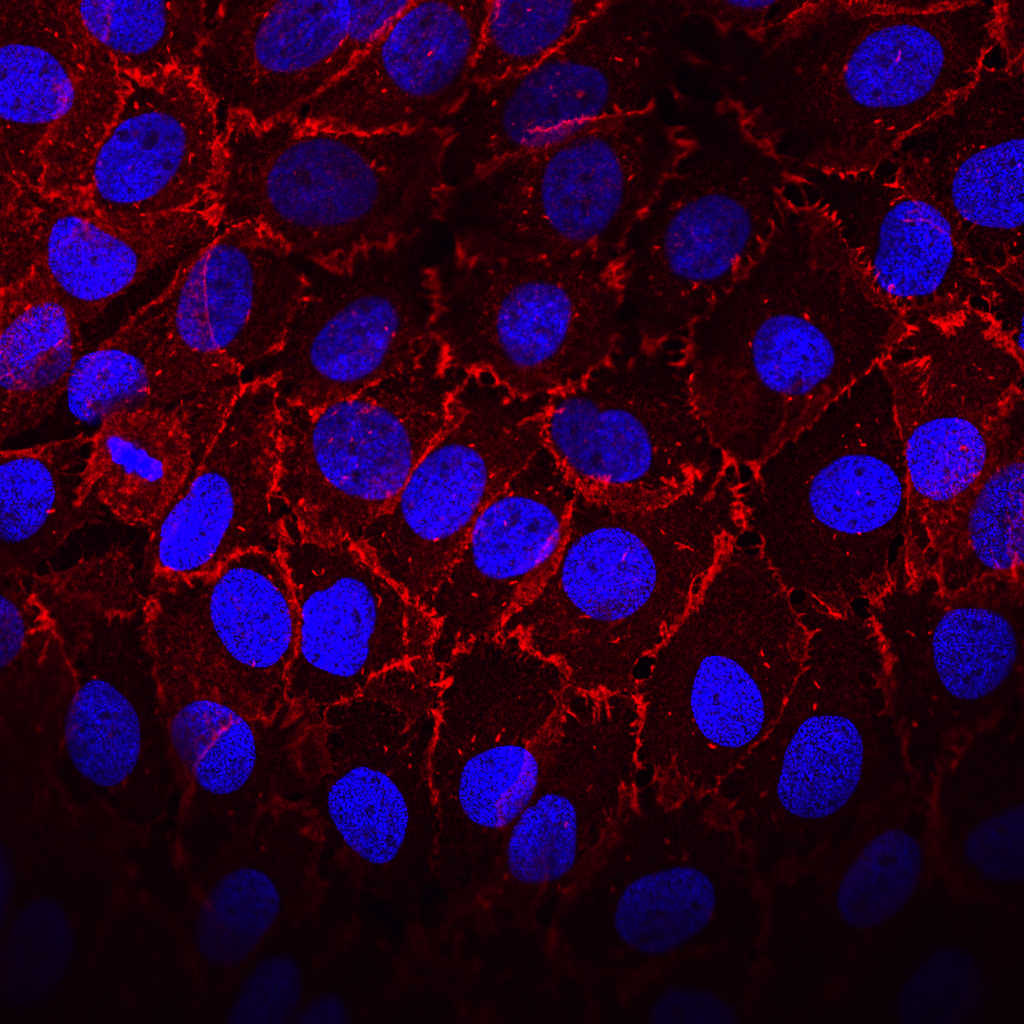

Supplement: Supplementary file 4 — Source Data Fig. 4 [file 44319_2024_104_MOESM4_ESM.zip › Figure 4/4B/Ctrl early.tif]

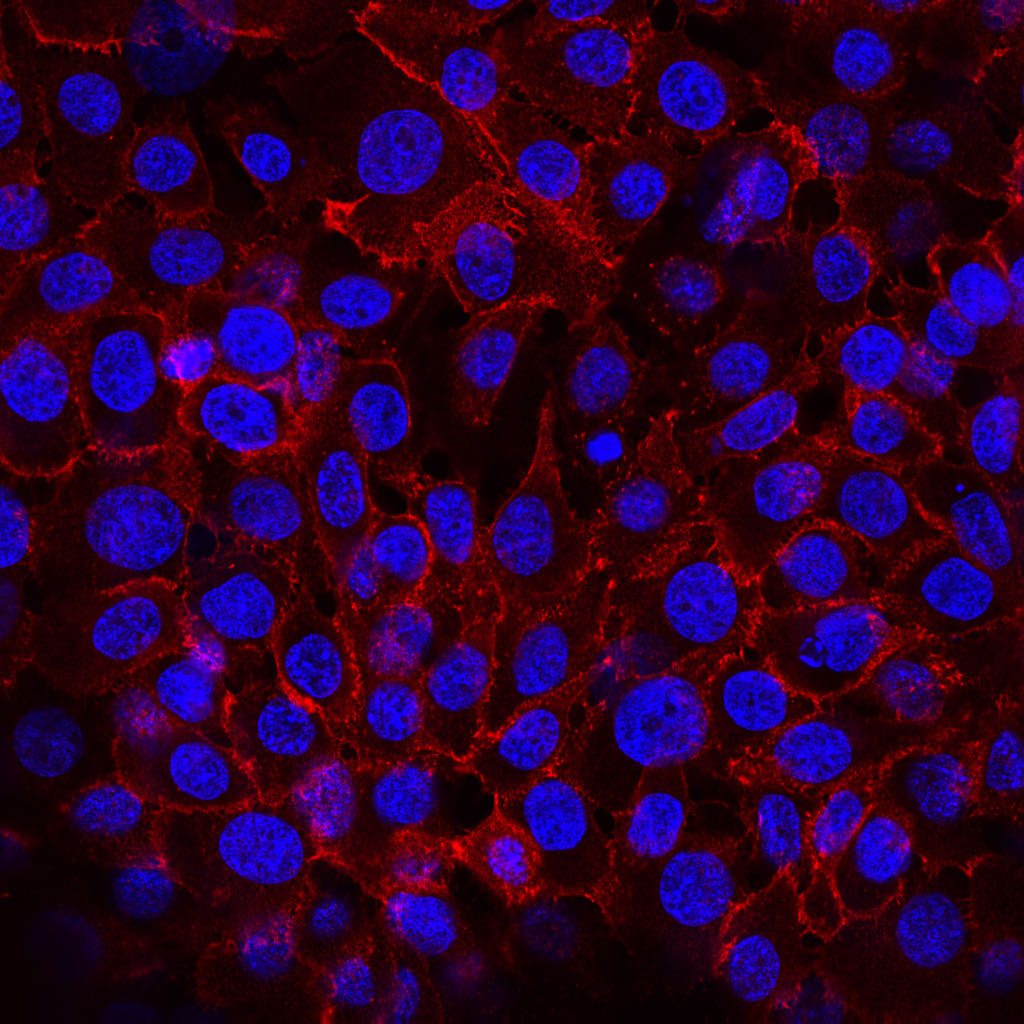

Supplement: Supplementary file 4 — Source Data Fig. 4 [file 44319_2024_104_MOESM4_ESM.zip › Figure 4/4B/Ctrl mature.tif]

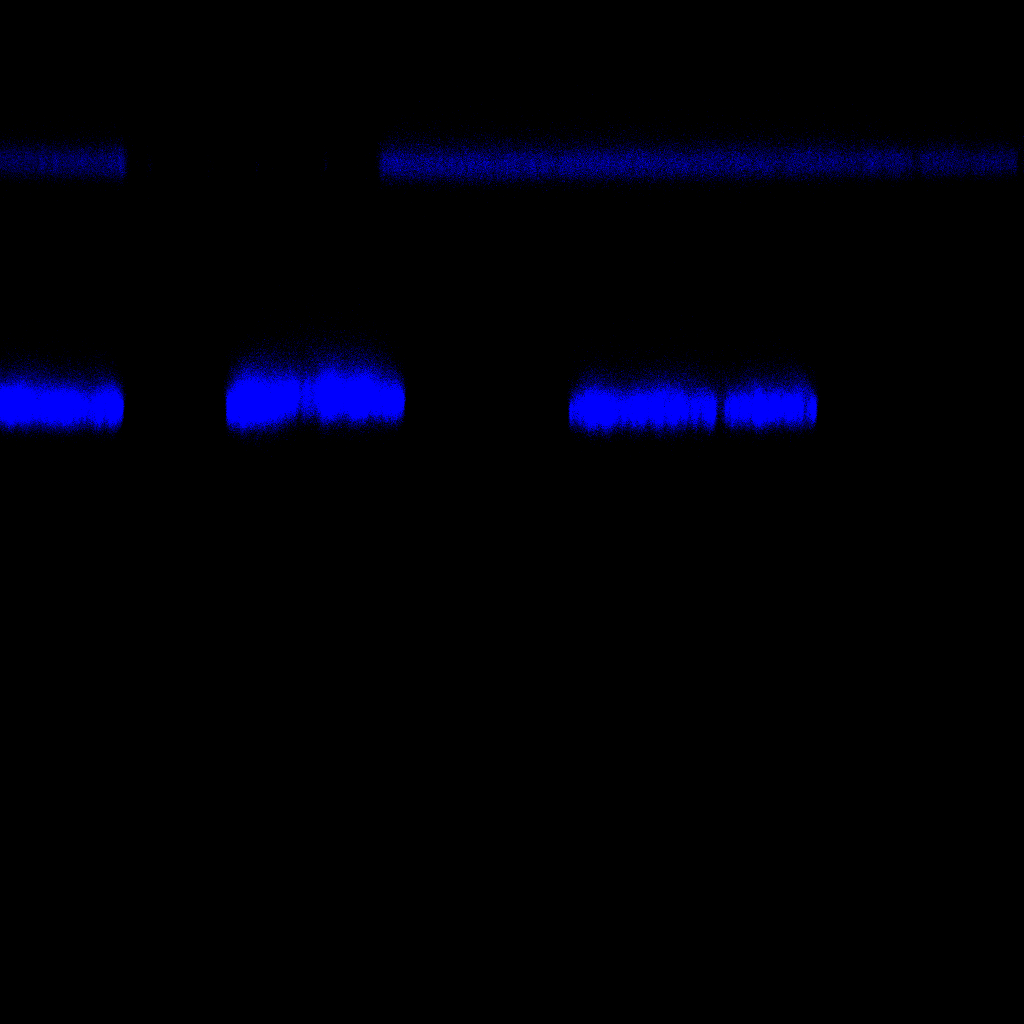

Supplement: Supplementary file 4 — Source Data Fig. 4 [file 44319_2024_104_MOESM4_ESM.zip › Figure 4/4B/Ctrl Z.tif]

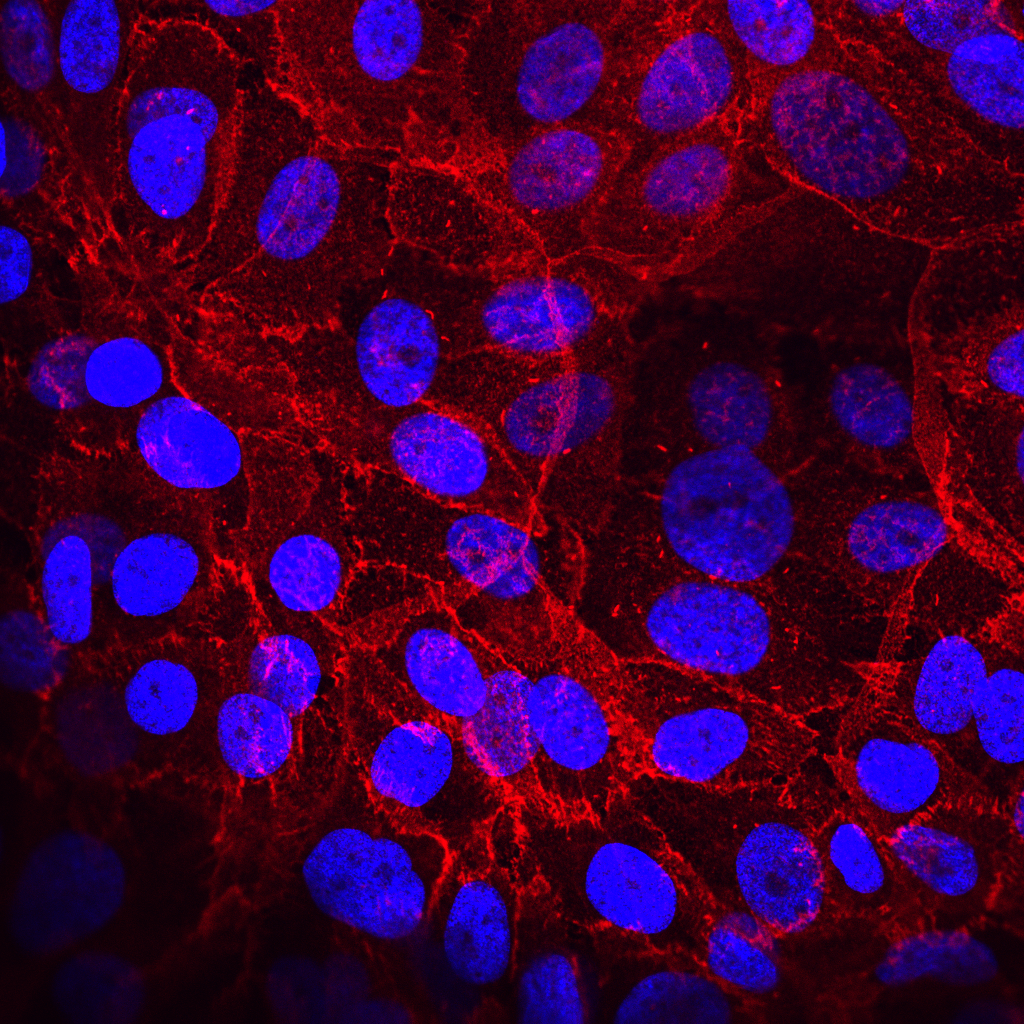

Supplement: Supplementary file 4 — Source Data Fig. 4 [file 44319_2024_104_MOESM4_ESM.zip › Figure 4/4B/shNLGN2 early.tif]

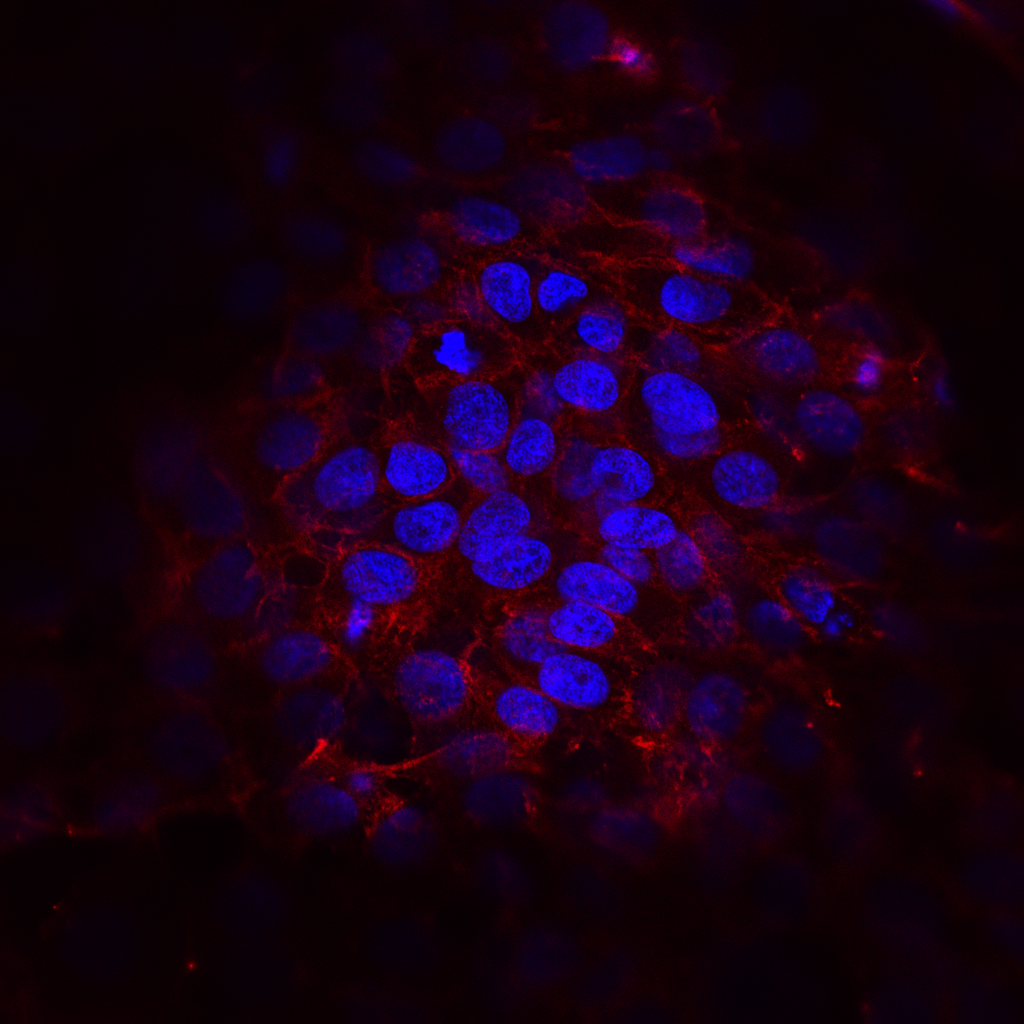

Supplement: Supplementary file 4 — Source Data Fig. 4 [file 44319_2024_104_MOESM4_ESM.zip › Figure 4/4B/shNLGN2 mature stacks/shNLGN2_z0.tif]

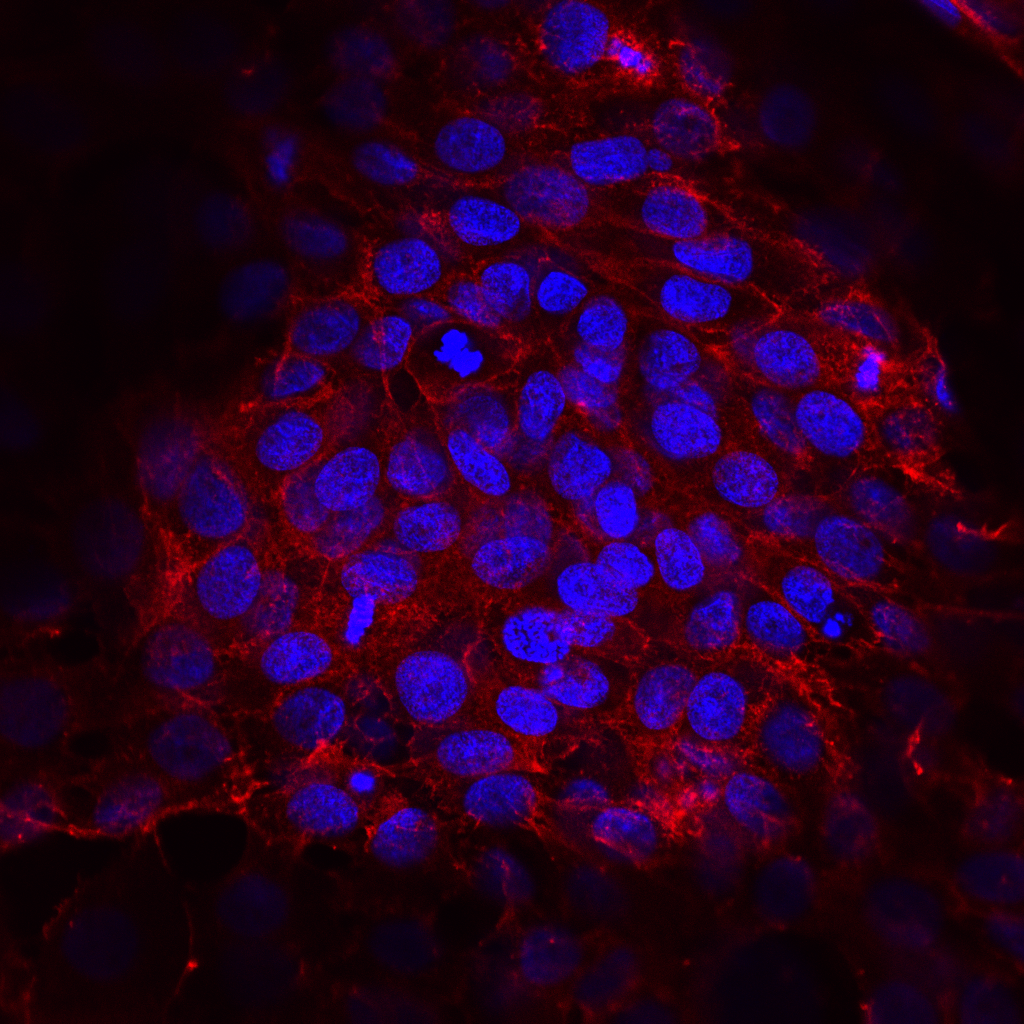

Supplement: Supplementary file 4 — Source Data Fig. 4 [file 44319_2024_104_MOESM4_ESM.zip › Figure 4/4B/shNLGN2 mature stacks/shNLGN2_z1.tif]

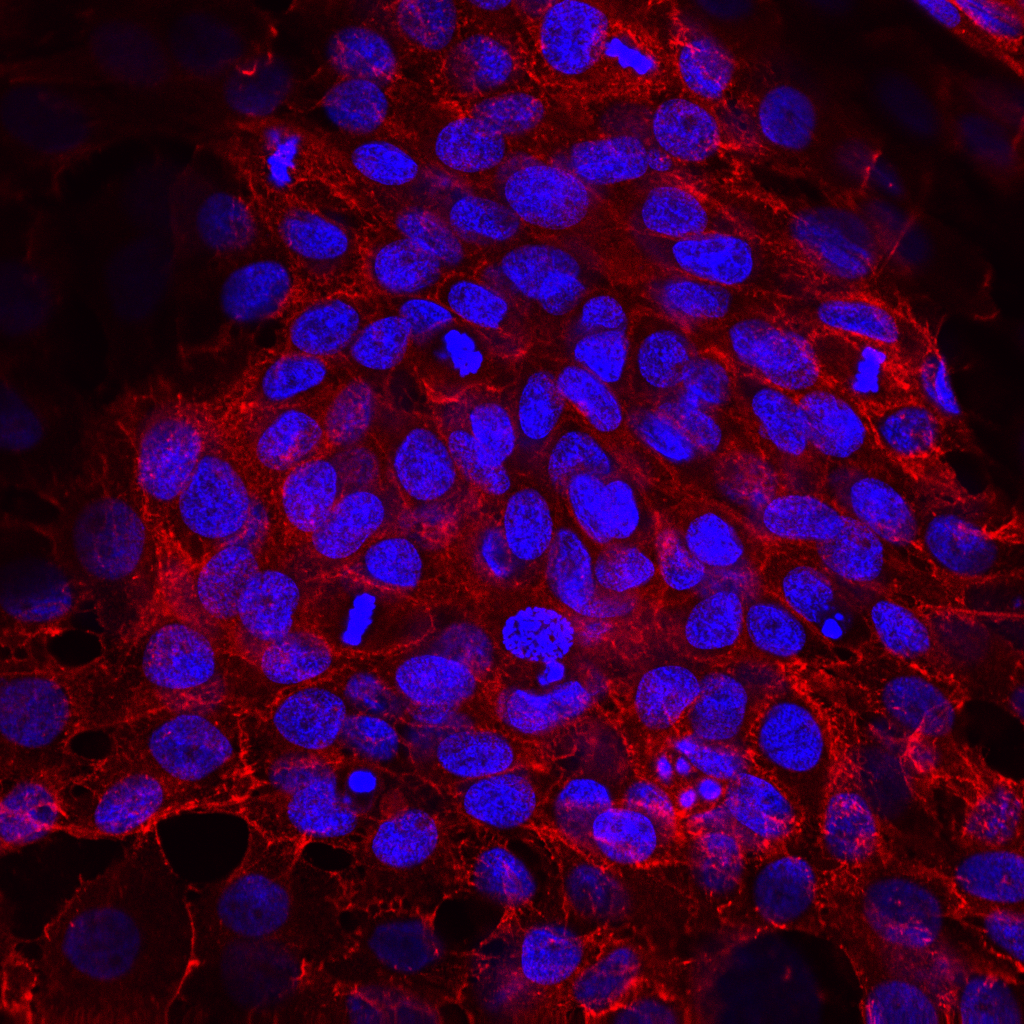

Supplement: Supplementary file 4 — Source Data Fig. 4 [file 44319_2024_104_MOESM4_ESM.zip › Figure 4/4B/shNLGN2 mature stacks/shNLGN2_z2.tif]

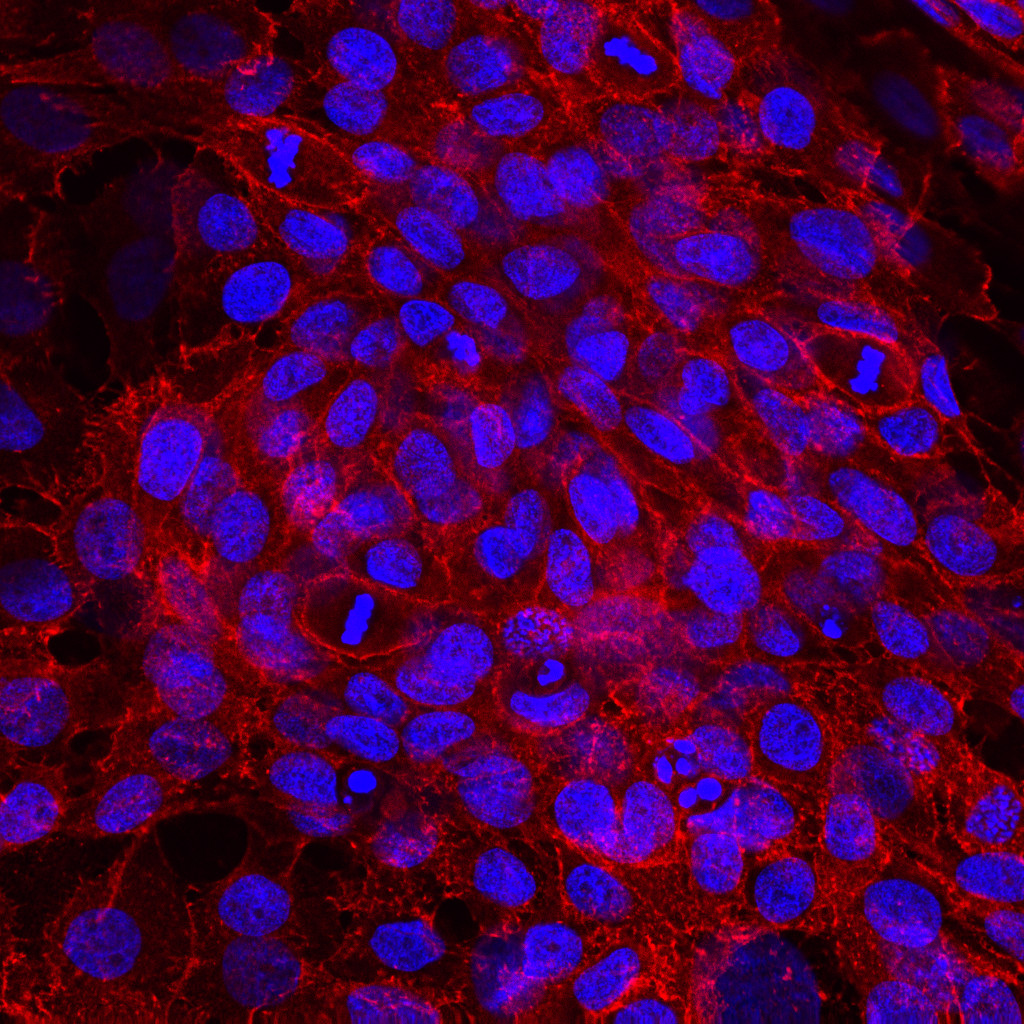

Supplement: Supplementary file 4 — Source Data Fig. 4 [file 44319_2024_104_MOESM4_ESM.zip › Figure 4/4B/shNLGN2 mature stacks/shNLGN2_z3.tif]

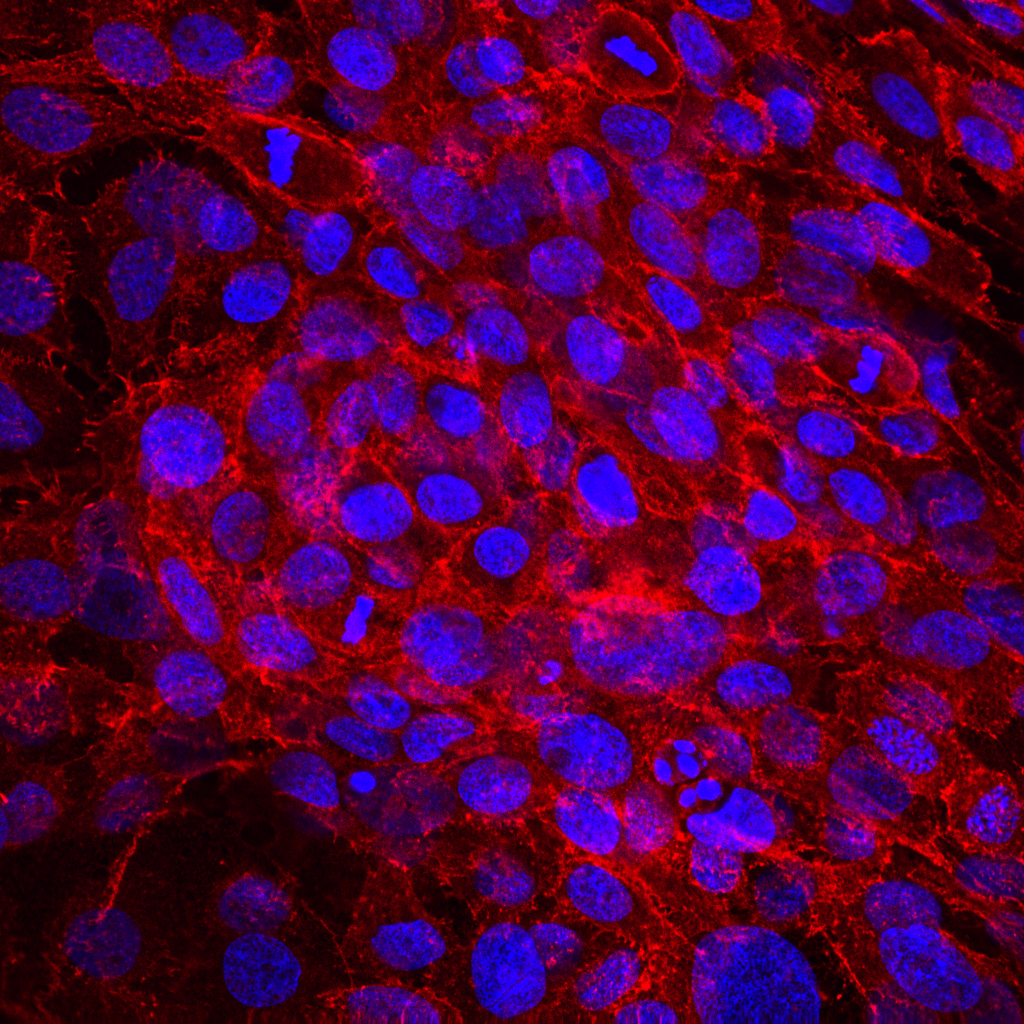

Supplement: Supplementary file 4 — Source Data Fig. 4 [file 44319_2024_104_MOESM4_ESM.zip › Figure 4/4B/shNLGN2 mature stacks/shNLGN2_z4.tif]

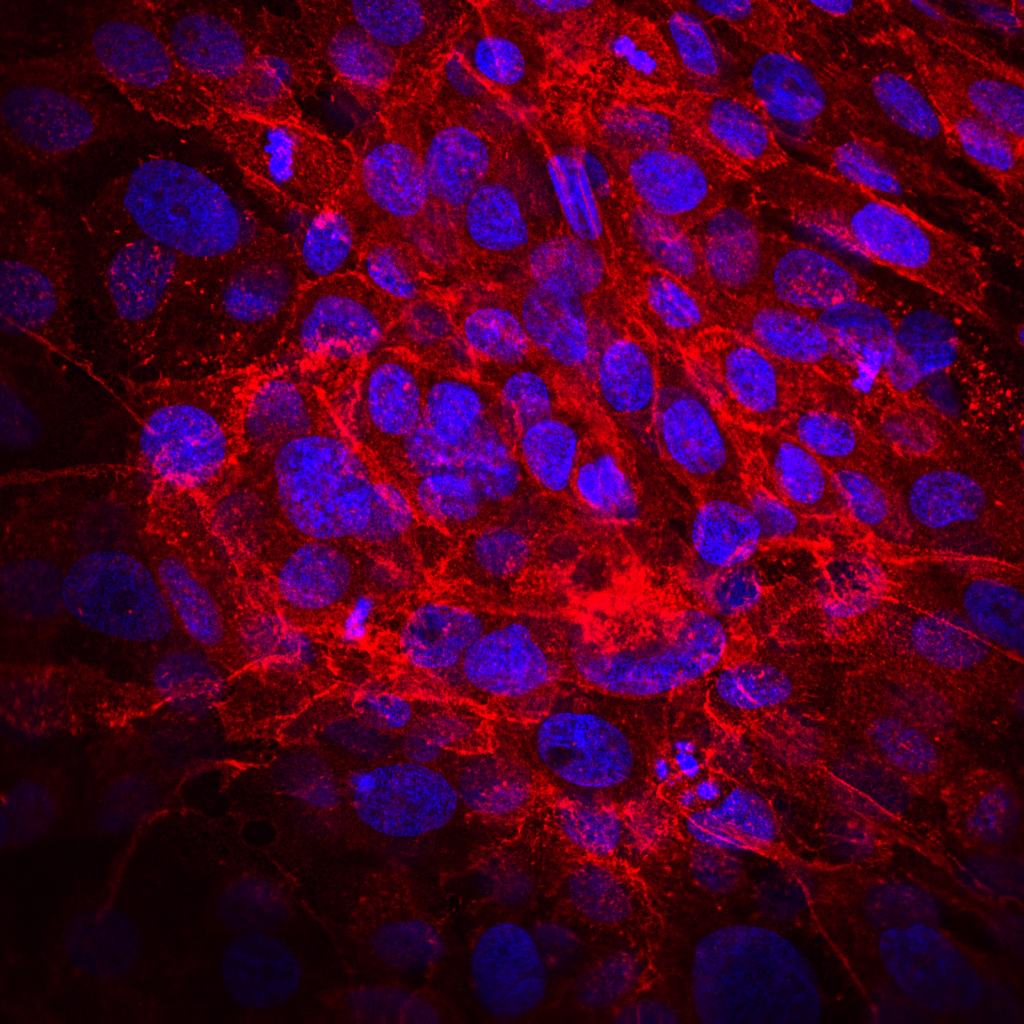

Supplement: Supplementary file 4 — Source Data Fig. 4 [file 44319_2024_104_MOESM4_ESM.zip › Figure 4/4B/shNLGN2 mature stacks/shNLGN2_z5.tif]

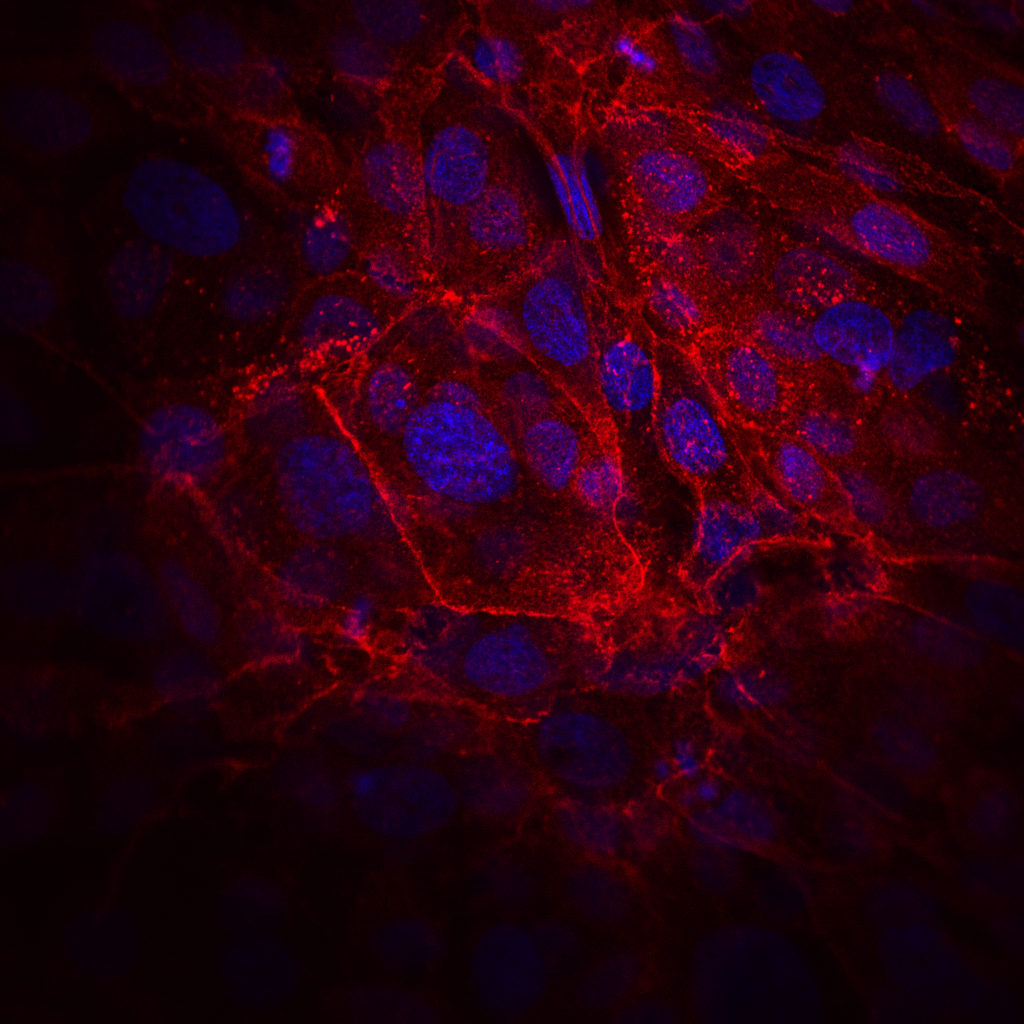

Supplement: Supplementary file 4 — Source Data Fig. 4 [file 44319_2024_104_MOESM4_ESM.zip › Figure 4/4B/shNLGN2 mature stacks/shNLGN2_z6.tif]

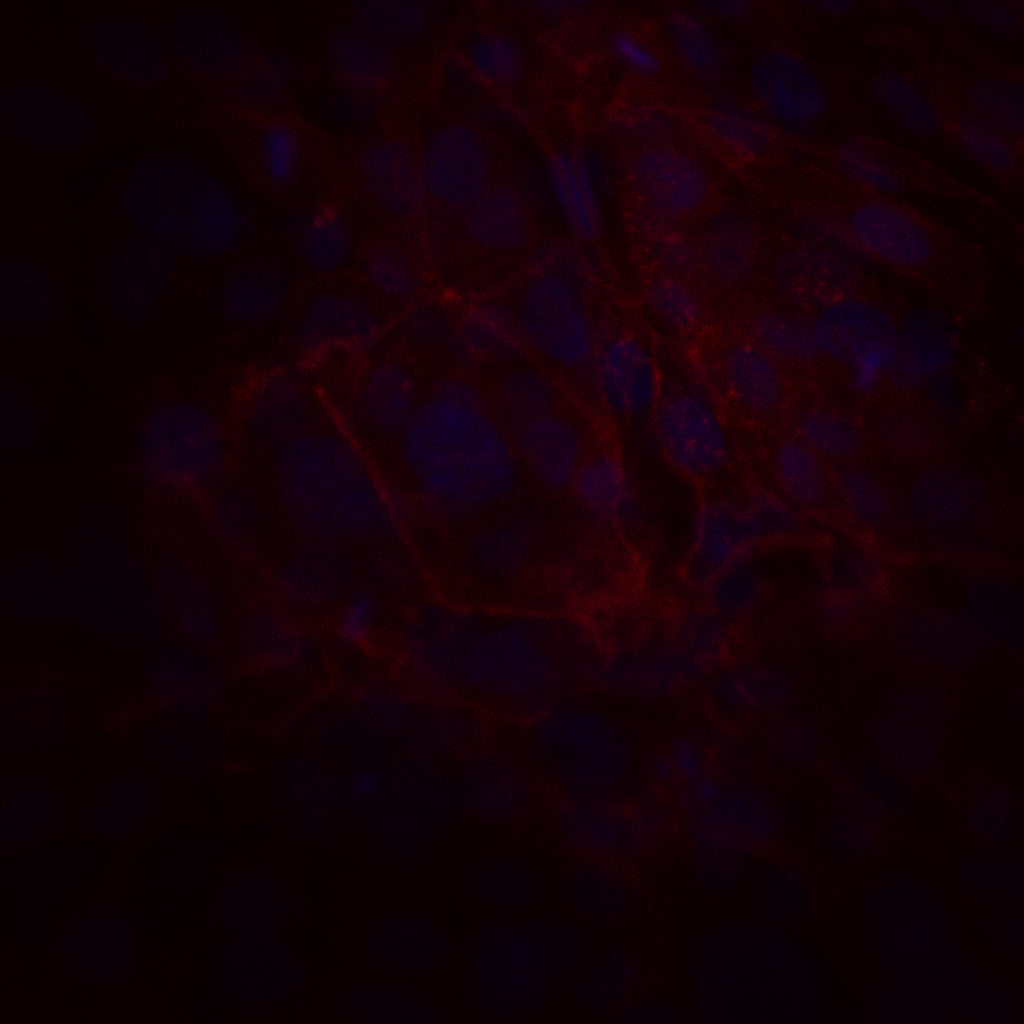

Supplement: Supplementary file 4 — Source Data Fig. 4 [file 44319_2024_104_MOESM4_ESM.zip › Figure 4/4B/shNLGN2 mature stacks/shNLGN2_z7.tif]

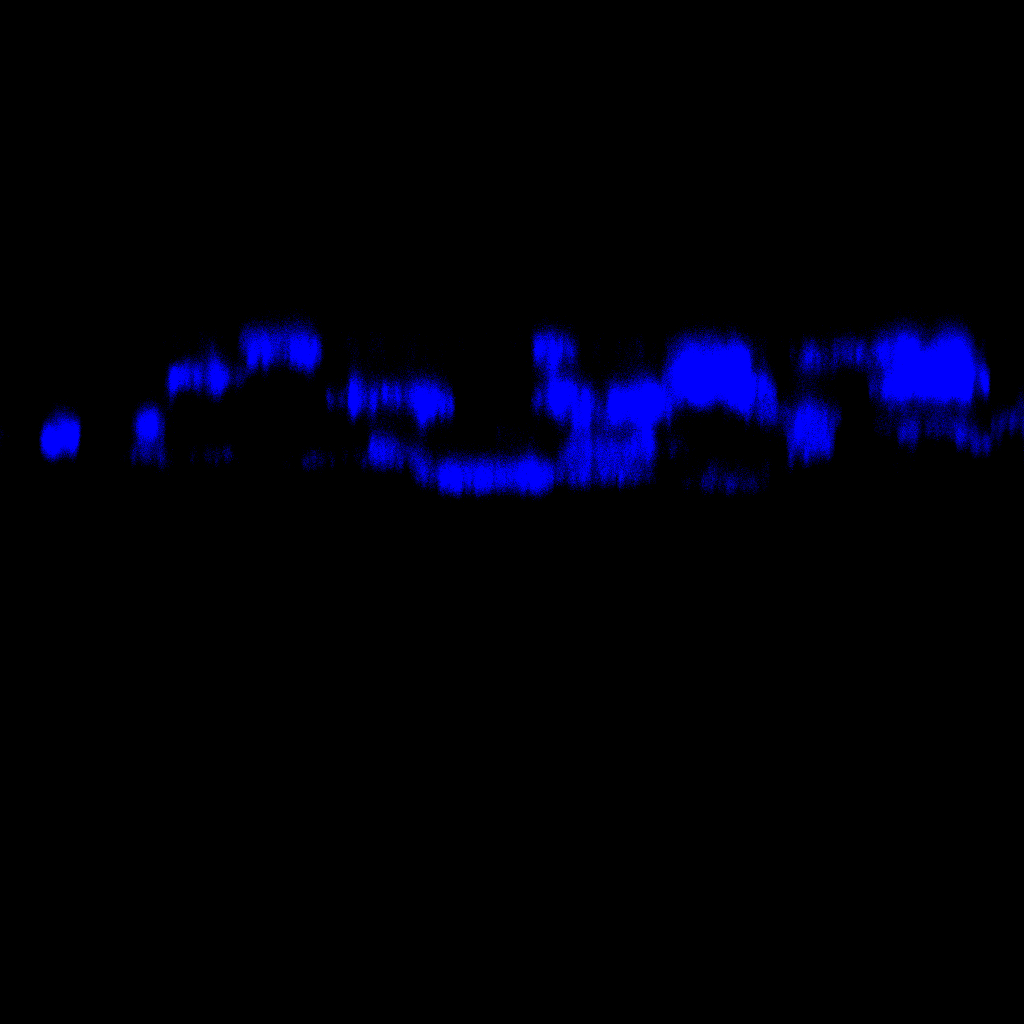

Supplement: Supplementary file 4 — Source Data Fig. 4 [file 44319_2024_104_MOESM4_ESM.zip › Figure 4/4B/shNLGN2 Z.tif]

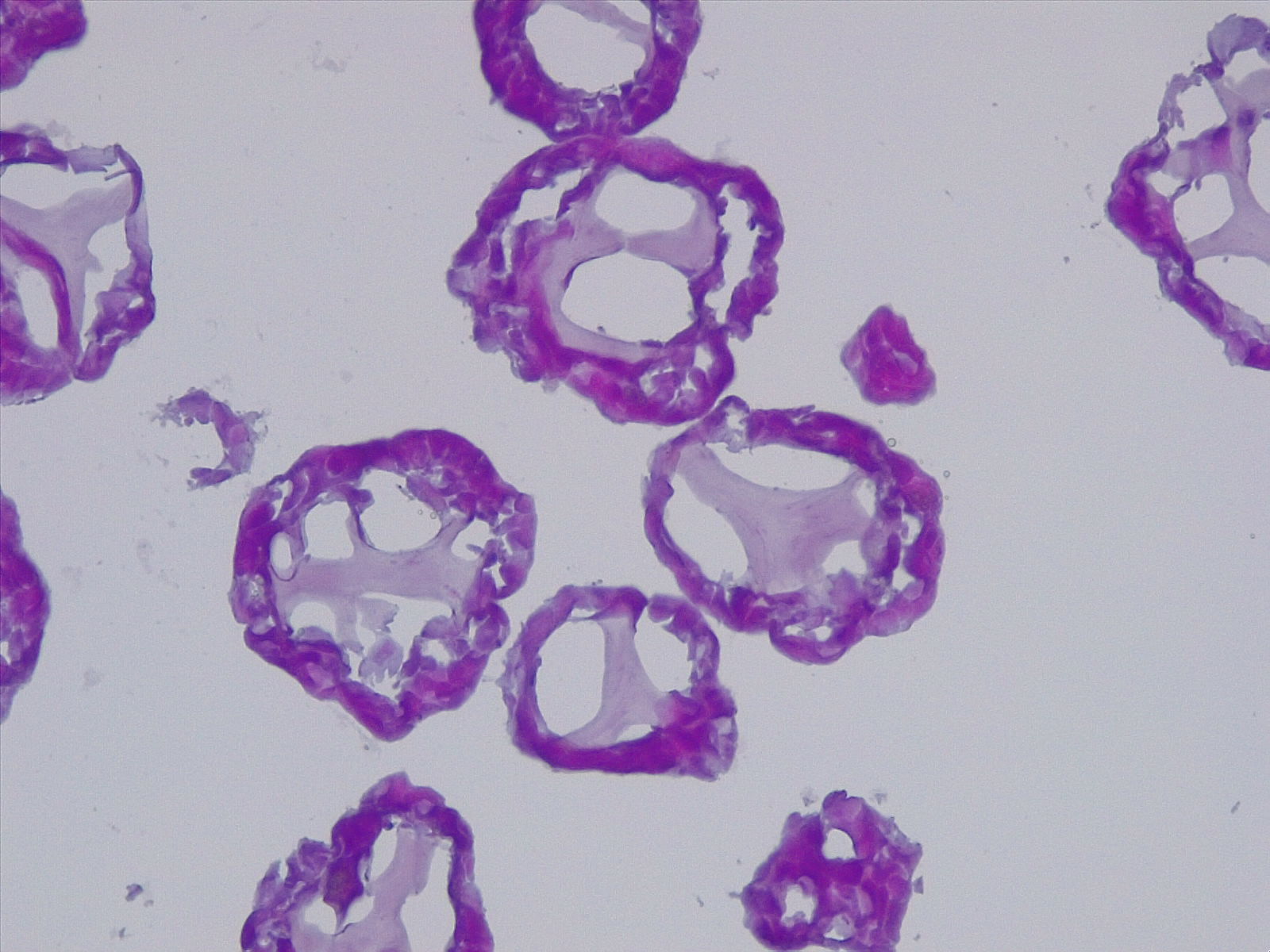

Supplement: Supplementary file 4 — Source Data Fig. 4 [file 44319_2024_104_MOESM4_ESM.zip › Figure 4/4C/Ctrl H&E.jpg]

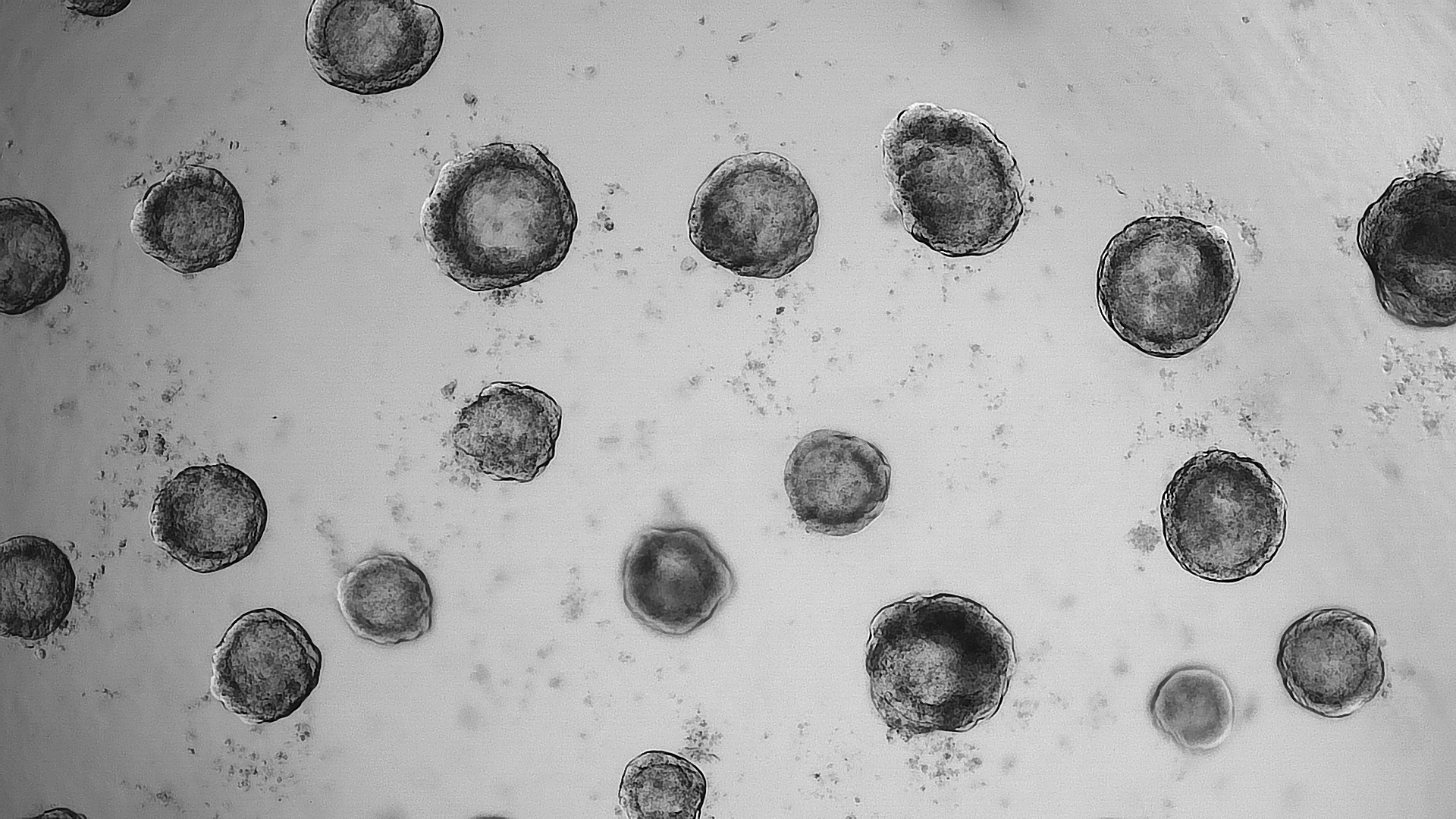

Supplement: Supplementary file 4 — Source Data Fig. 4 [file 44319_2024_104_MOESM4_ESM.zip › Figure 4/4C/Ctrl.tiff]

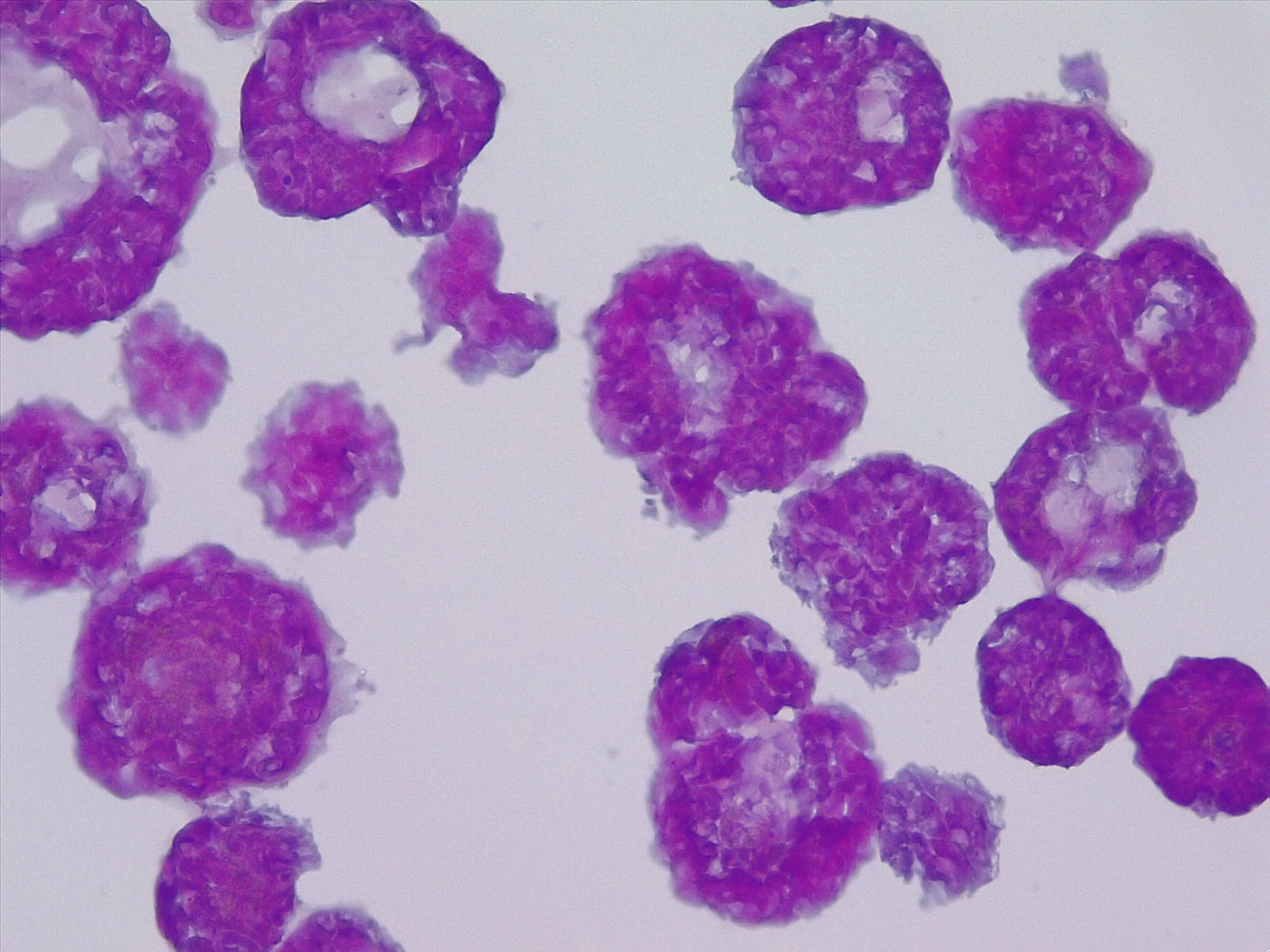

Supplement: Supplementary file 4 — Source Data Fig. 4 [file 44319_2024_104_MOESM4_ESM.zip › Figure 4/4C/shNLGN2 H&E.jpg]

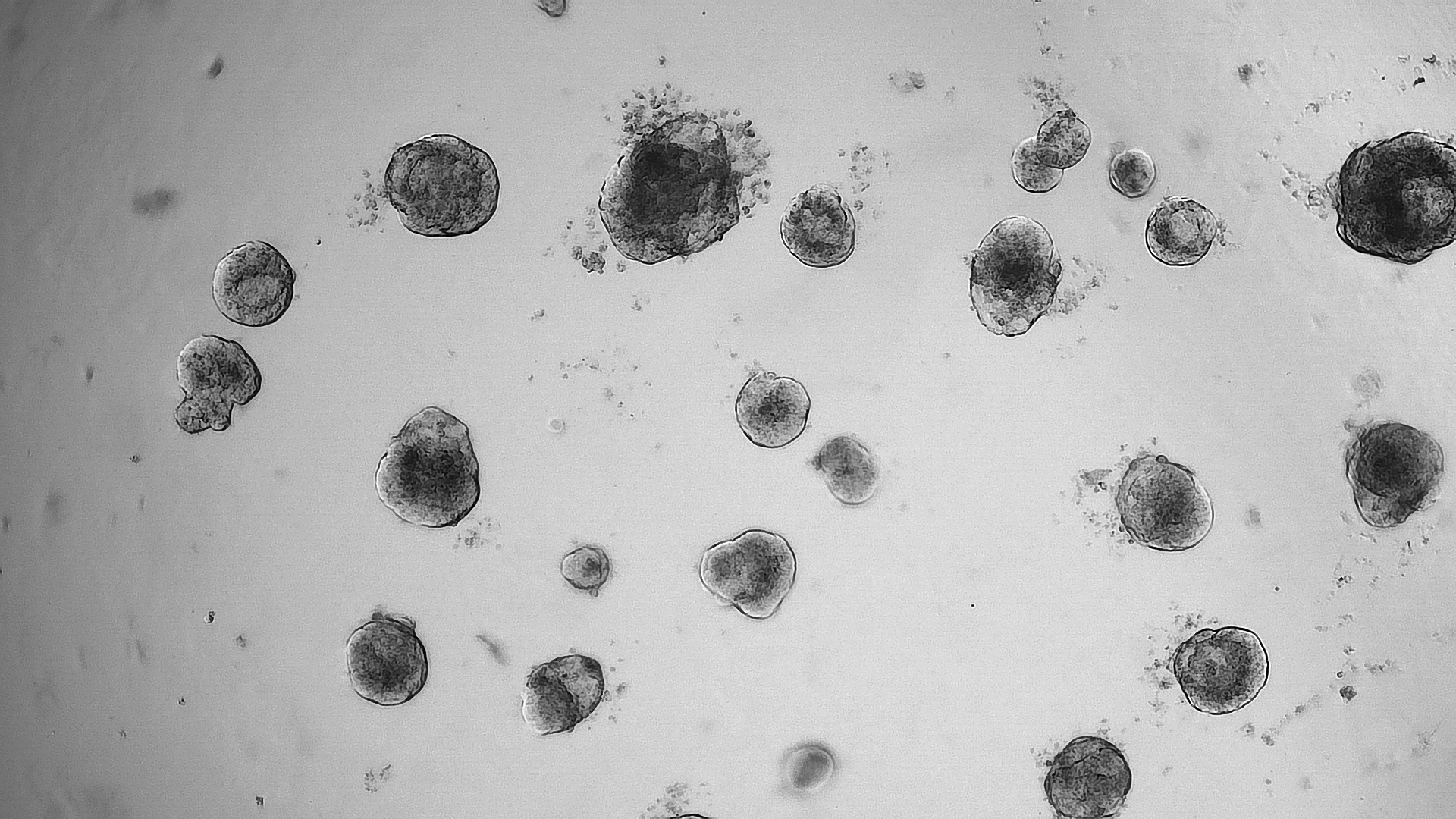

Supplement: Supplementary file 4 — Source Data Fig. 4 [file 44319_2024_104_MOESM4_ESM.zip › Figure 4/4C/shNLGN2.tiff]

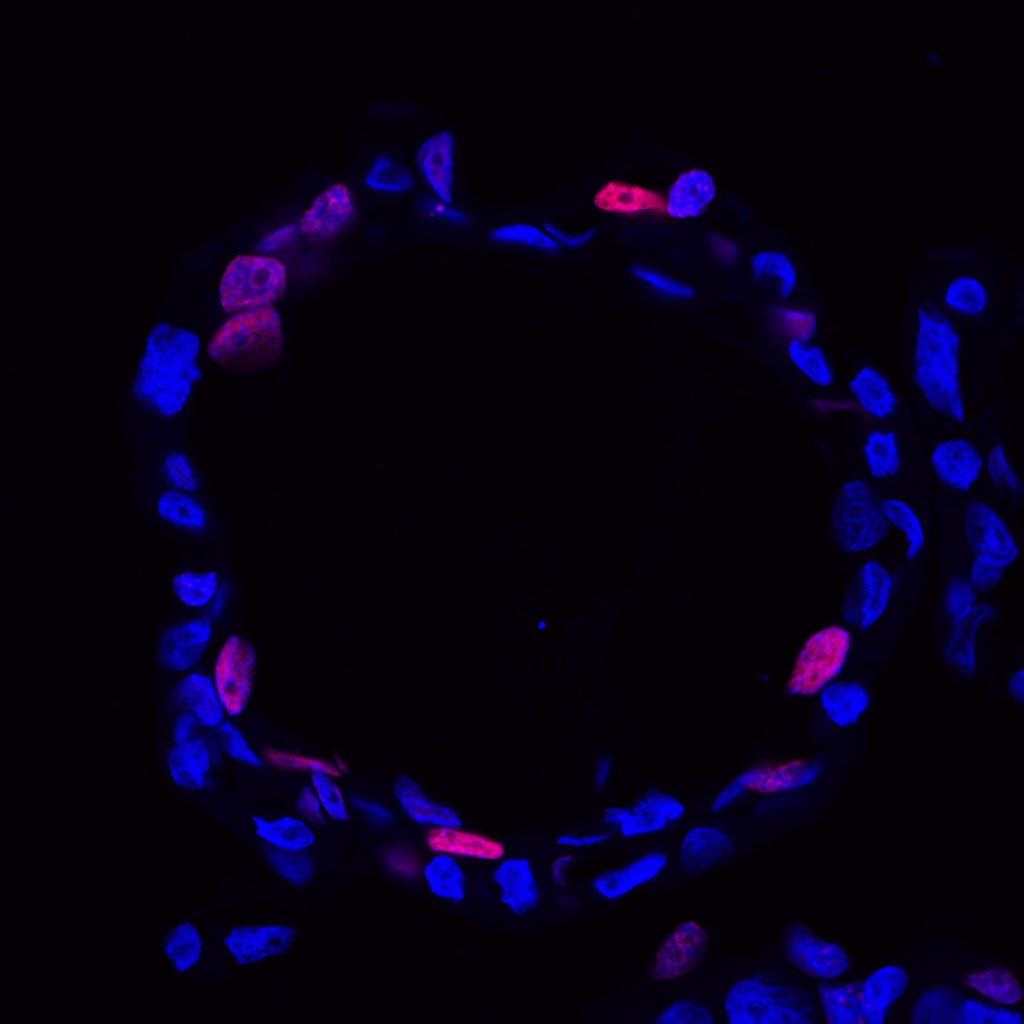

Supplement: Supplementary file 4 — Source Data Fig. 4 [file 44319_2024_104_MOESM4_ESM.zip › Figure 4/4D/Ctrl DAPI EdU.tif]

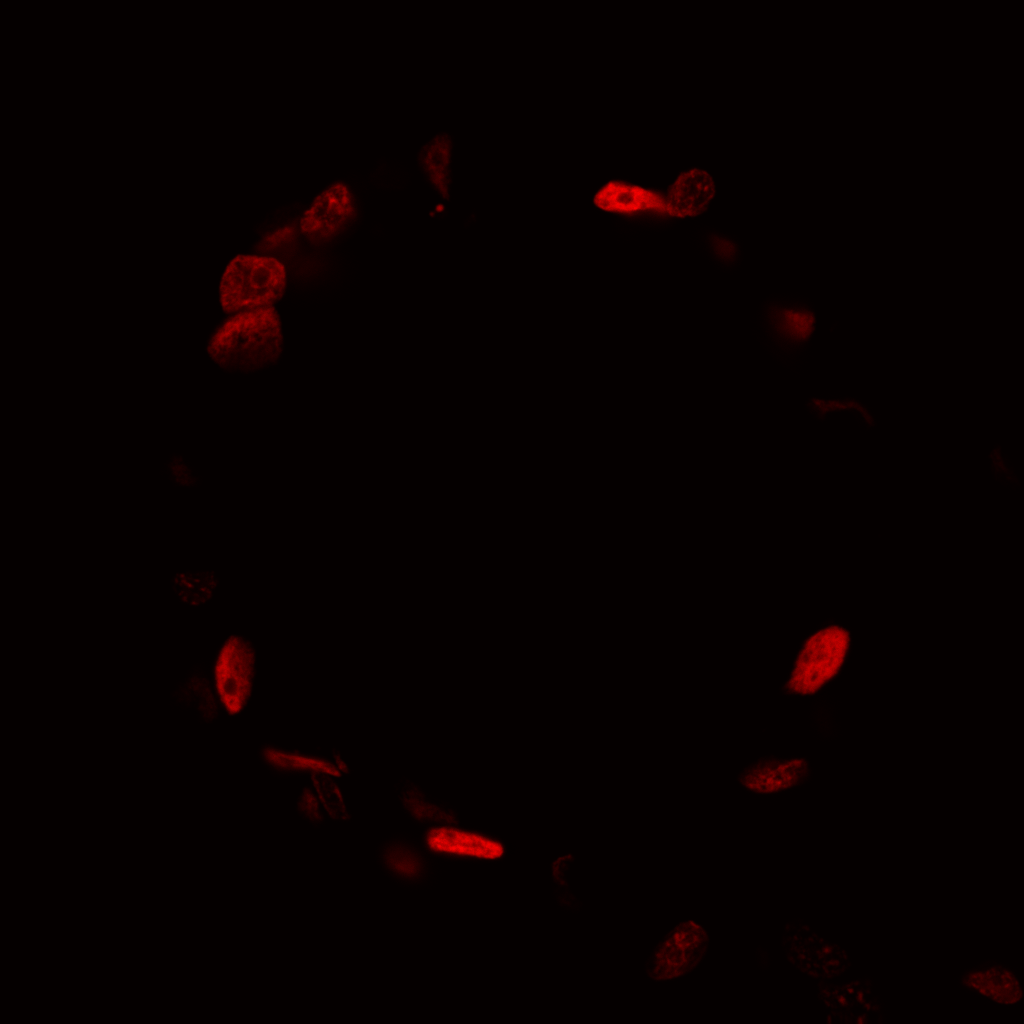

Supplement: Supplementary file 4 — Source Data Fig. 4 [file 44319_2024_104_MOESM4_ESM.zip › Figure 4/4D/Ctrl EdU.tif]

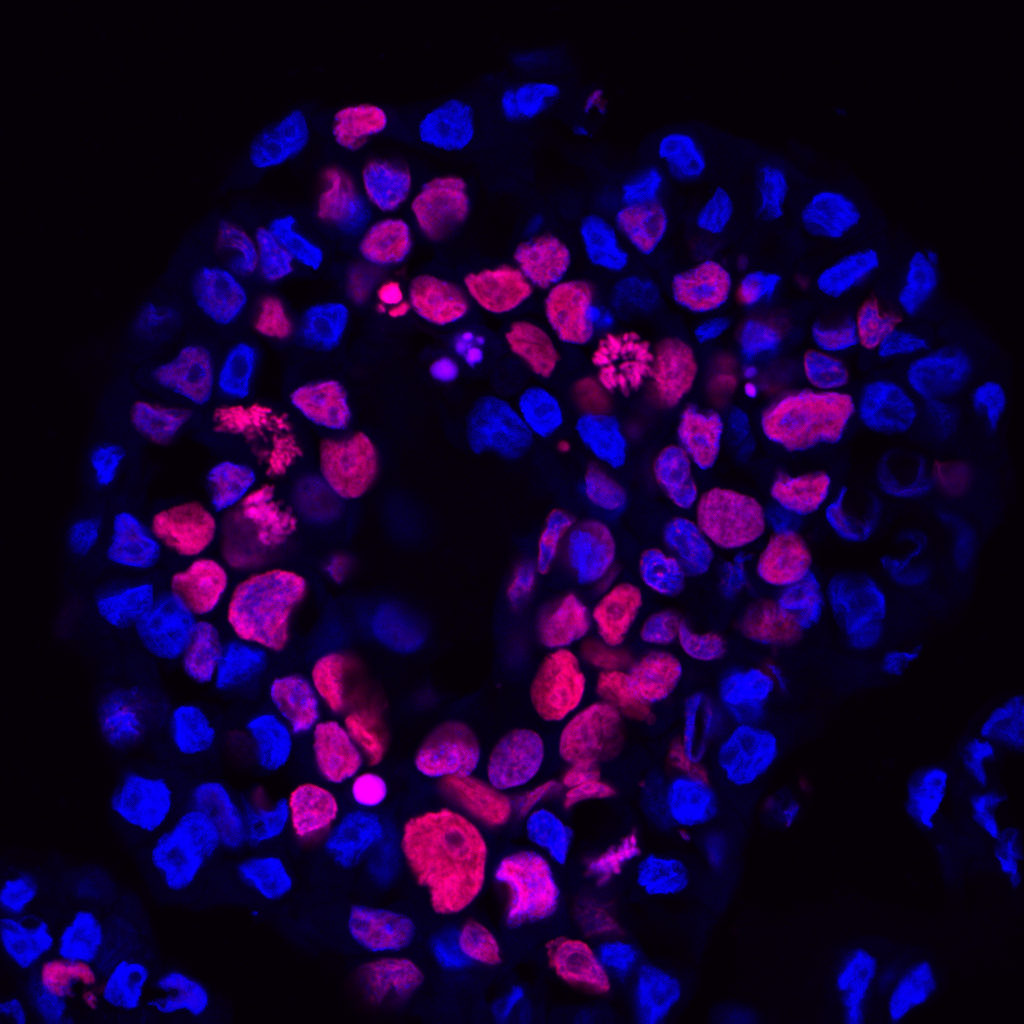

Supplement: Supplementary file 4 — Source Data Fig. 4 [file 44319_2024_104_MOESM4_ESM.zip › Figure 4/4D/shNLGN2 DAPI EdU.tif]

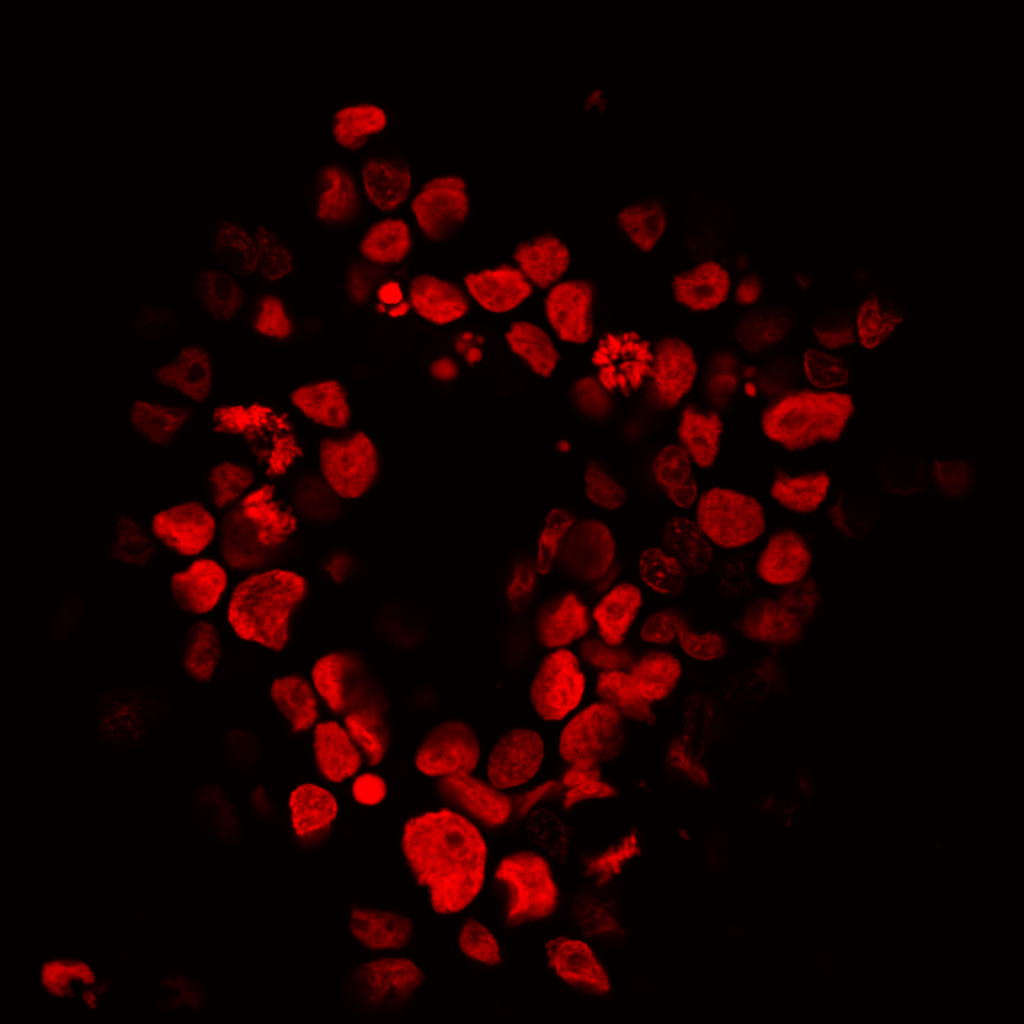

Supplement: Supplementary file 4 — Source Data Fig. 4 [file 44319_2024_104_MOESM4_ESM.zip › Figure 4/4D/shNLGN2 EdU.tif]

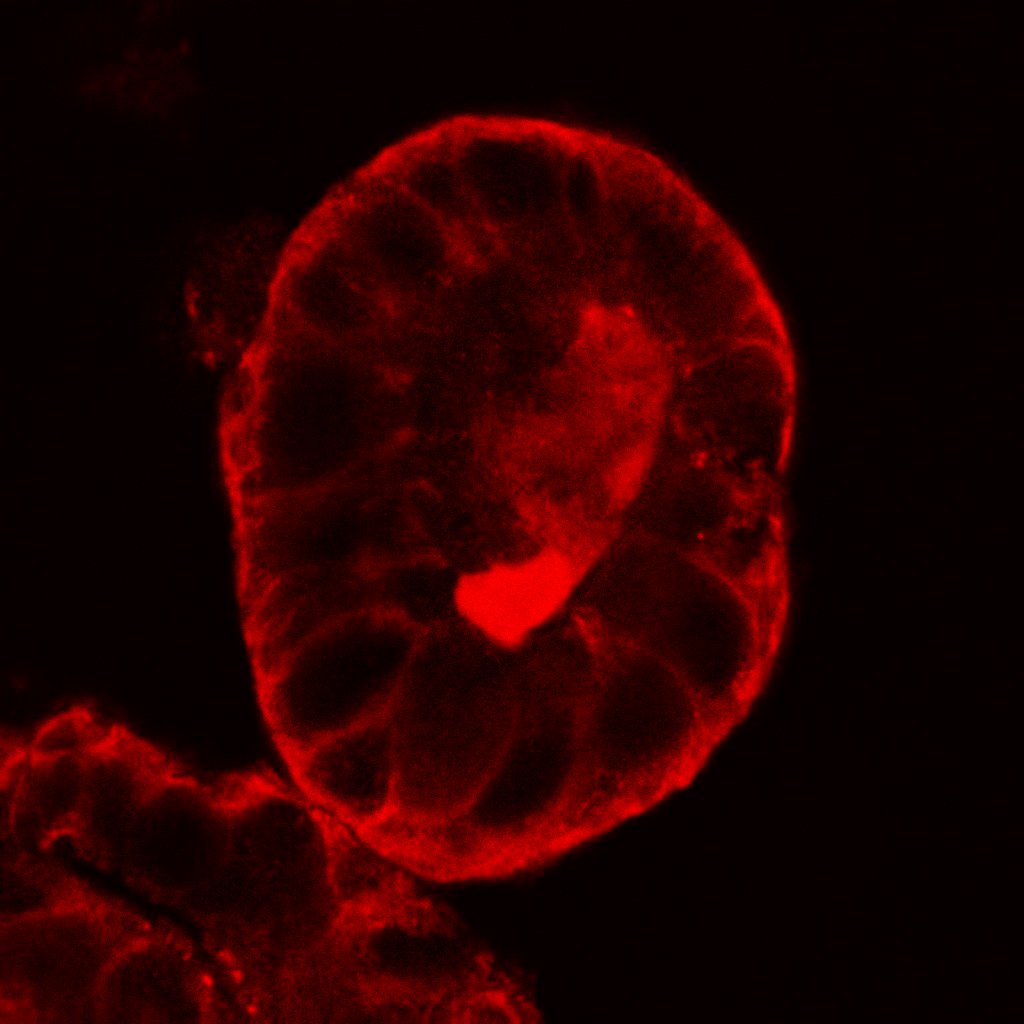

Supplement: Supplementary file 4 — Source Data Fig. 4 [file 44319_2024_104_MOESM4_ESM.zip › Figure 4/4F/Ctrl and rescue ECAD.jpg]

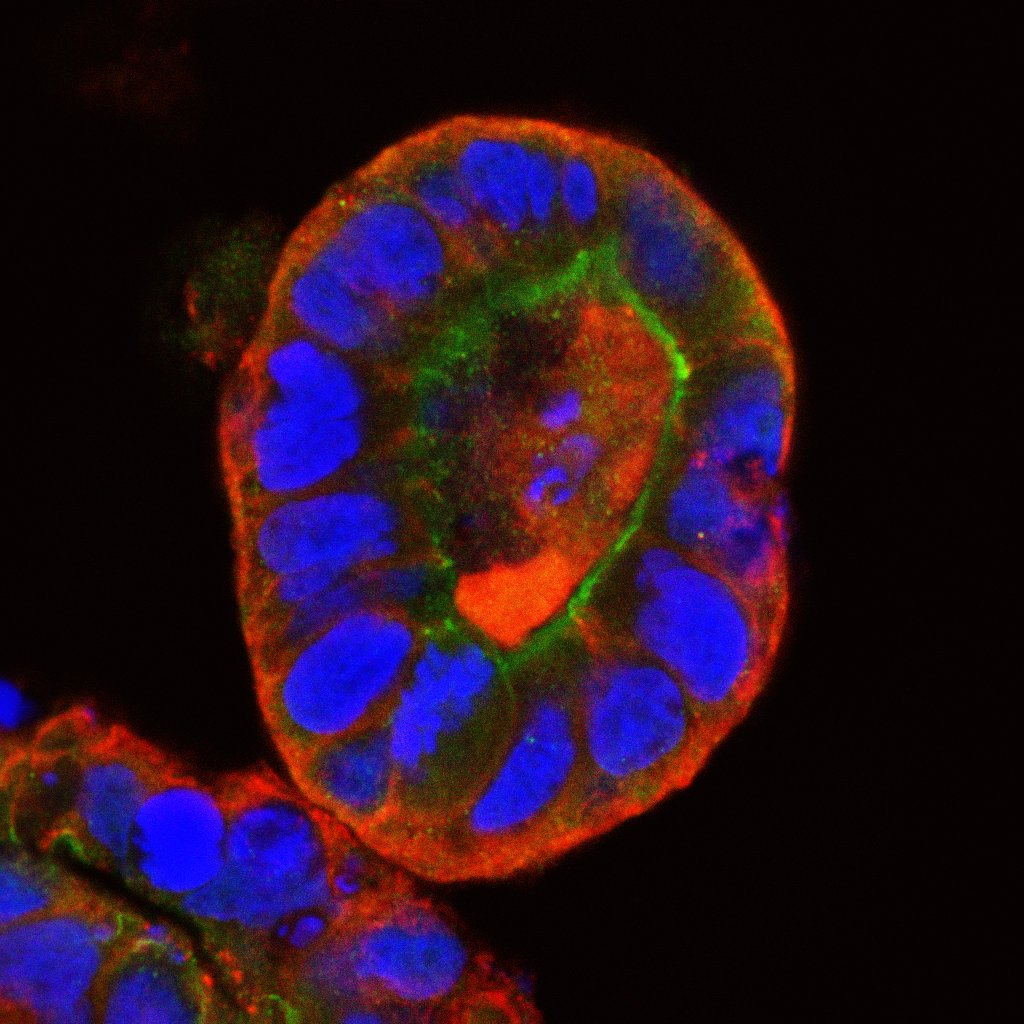

Supplement: Supplementary file 4 — Source Data Fig. 4 [file 44319_2024_104_MOESM4_ESM.zip › Figure 4/4F/Ctrl and rescue Merge.tif]

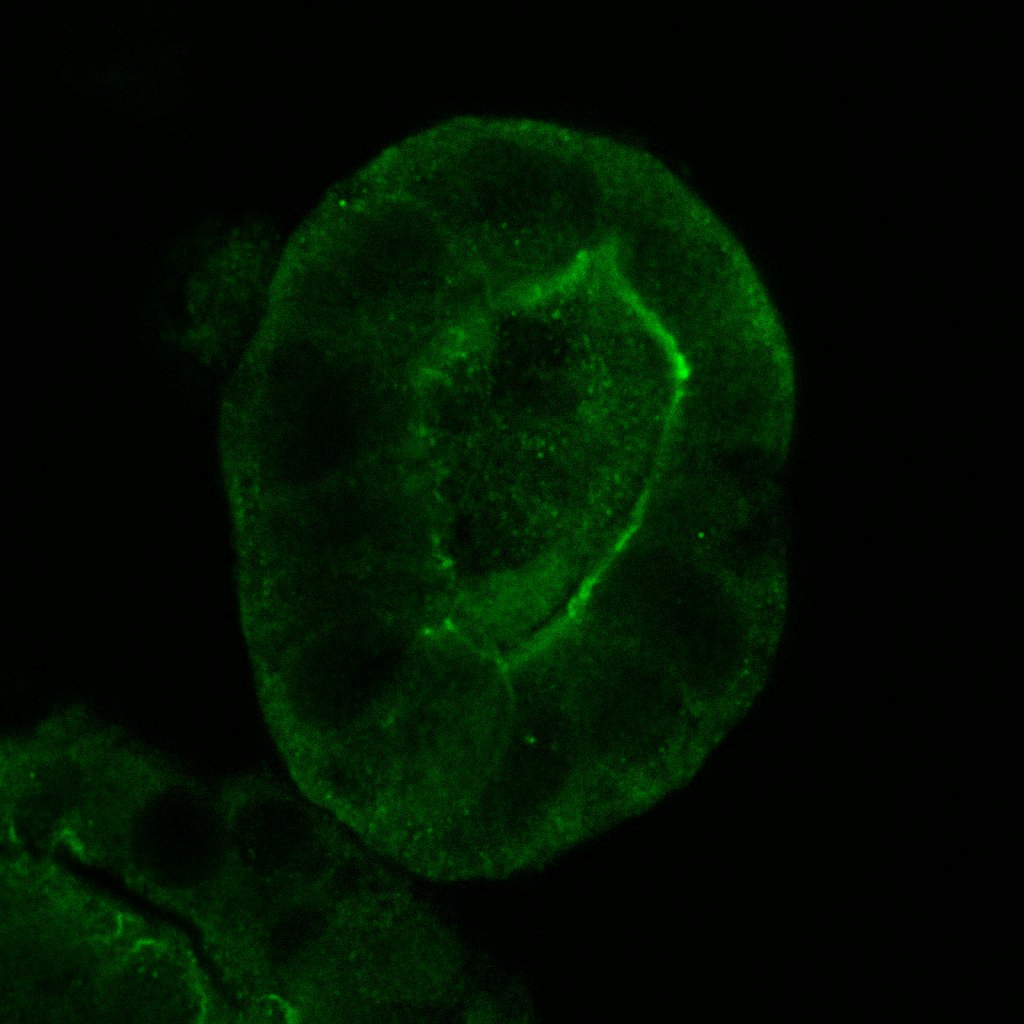

Supplement: Supplementary file 4 — Source Data Fig. 4 [file 44319_2024_104_MOESM4_ESM.zip › Figure 4/4F/Ctrl and rescue ZO1.jpg]

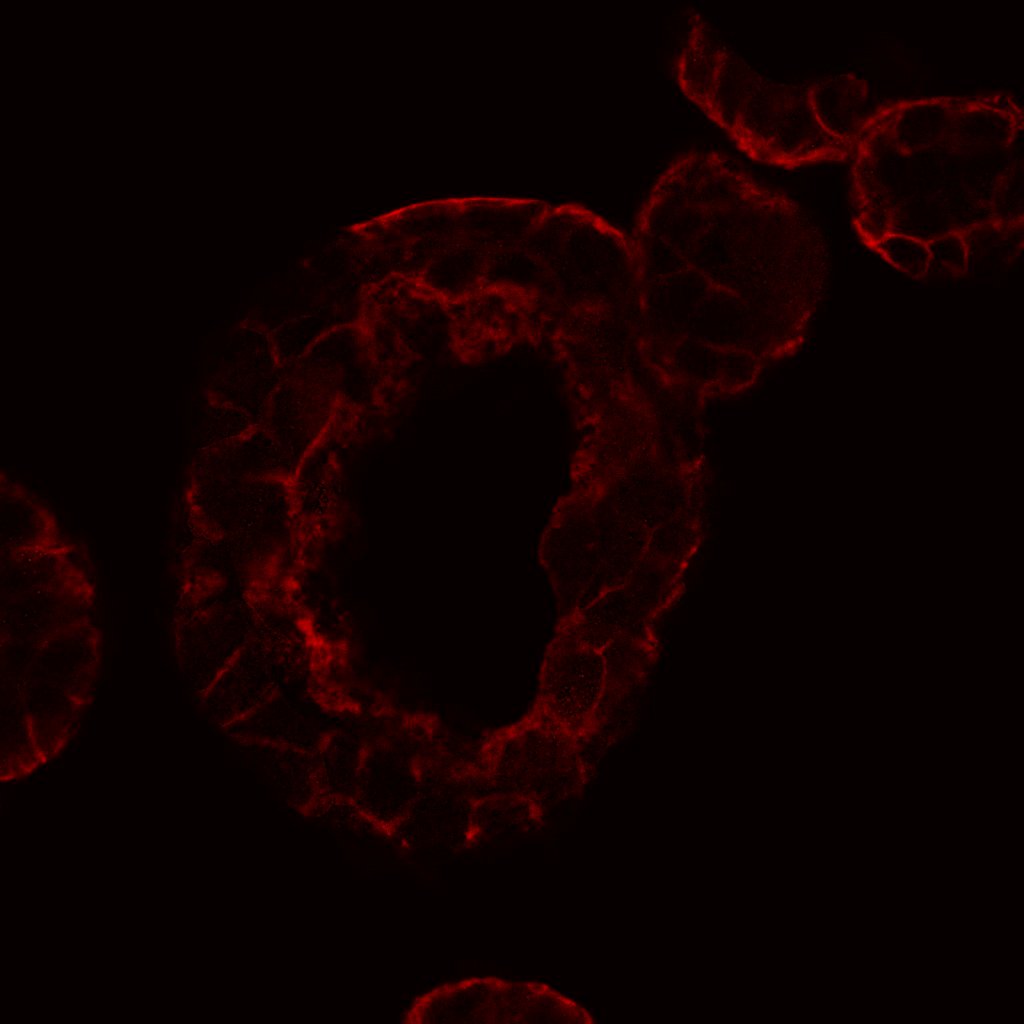

Supplement: Supplementary file 4 — Source Data Fig. 4 [file 44319_2024_104_MOESM4_ESM.zip › Figure 4/4F/shNLGN2 ECAD.jpg]

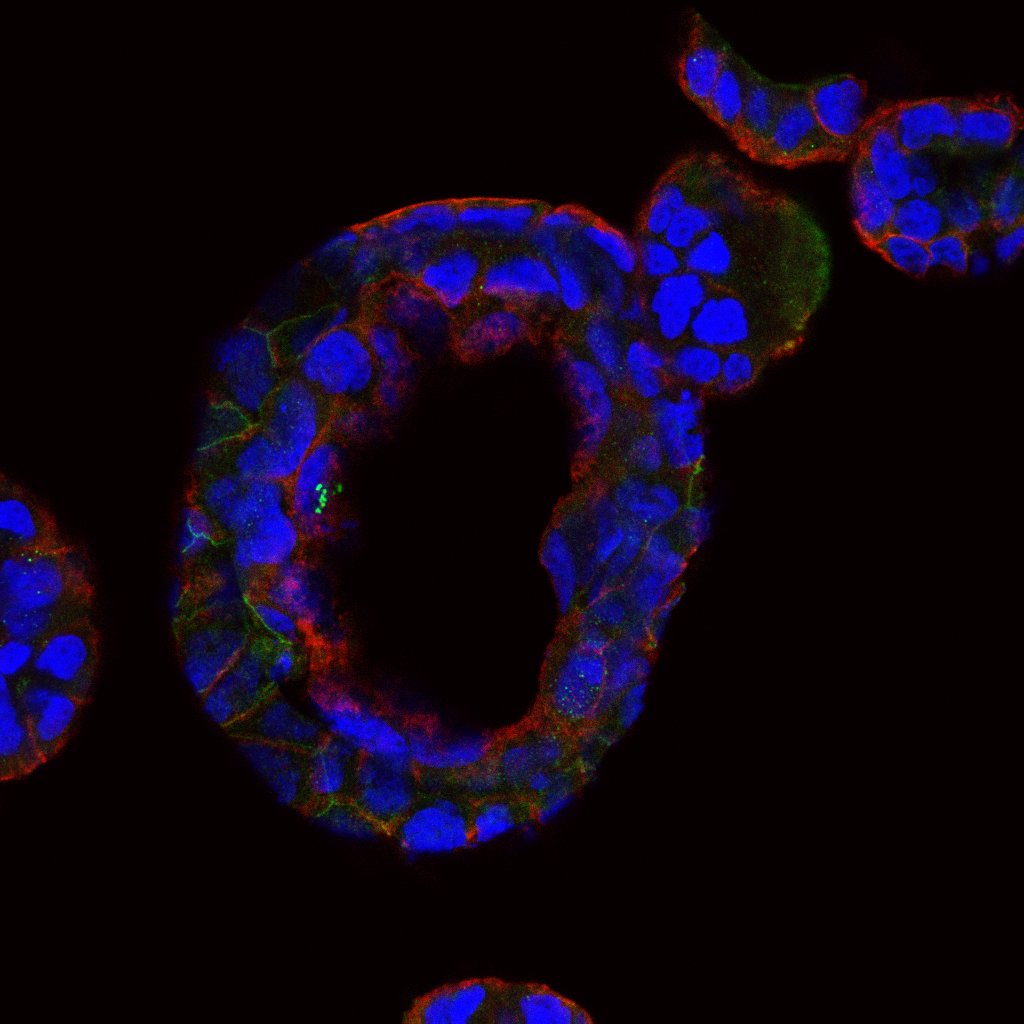

Supplement: Supplementary file 4 — Source Data Fig. 4 [file 44319_2024_104_MOESM4_ESM.zip › Figure 4/4F/shNLGN2 Merge.jpg]

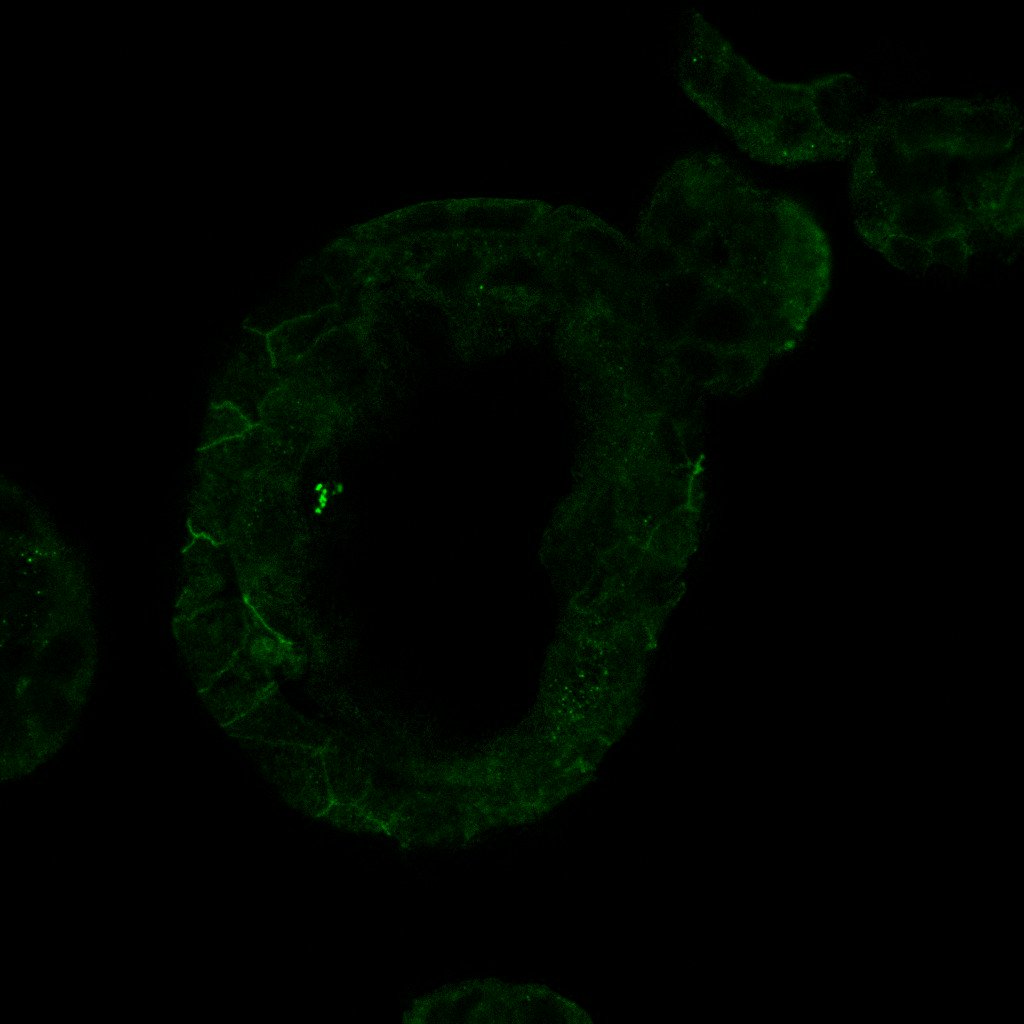

Supplement: Supplementary file 4 — Source Data Fig. 4 [file 44319_2024_104_MOESM4_ESM.zip › Figure 4/4F/shNLGN2 ZO1.jpg]

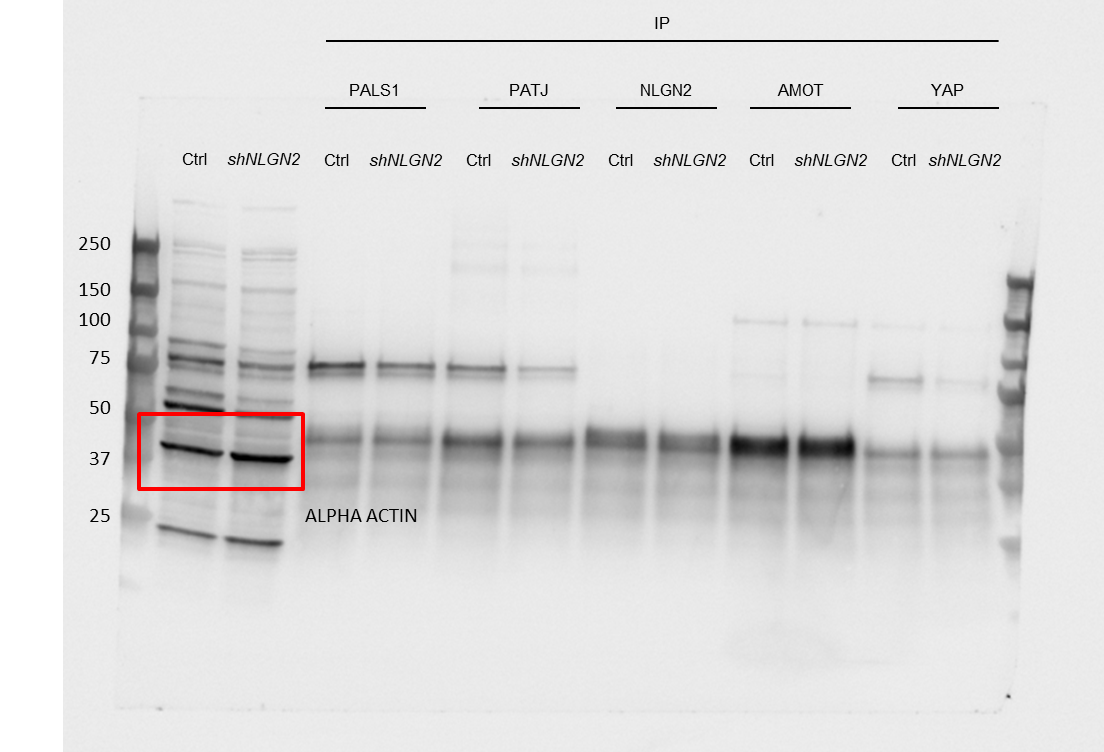

Supplement: Supplementary file 5 — Source Data Fig. 5 [file 44319_2024_104_MOESM5_ESM.zip › Figure 5/5A/WB ACTIN.tif]

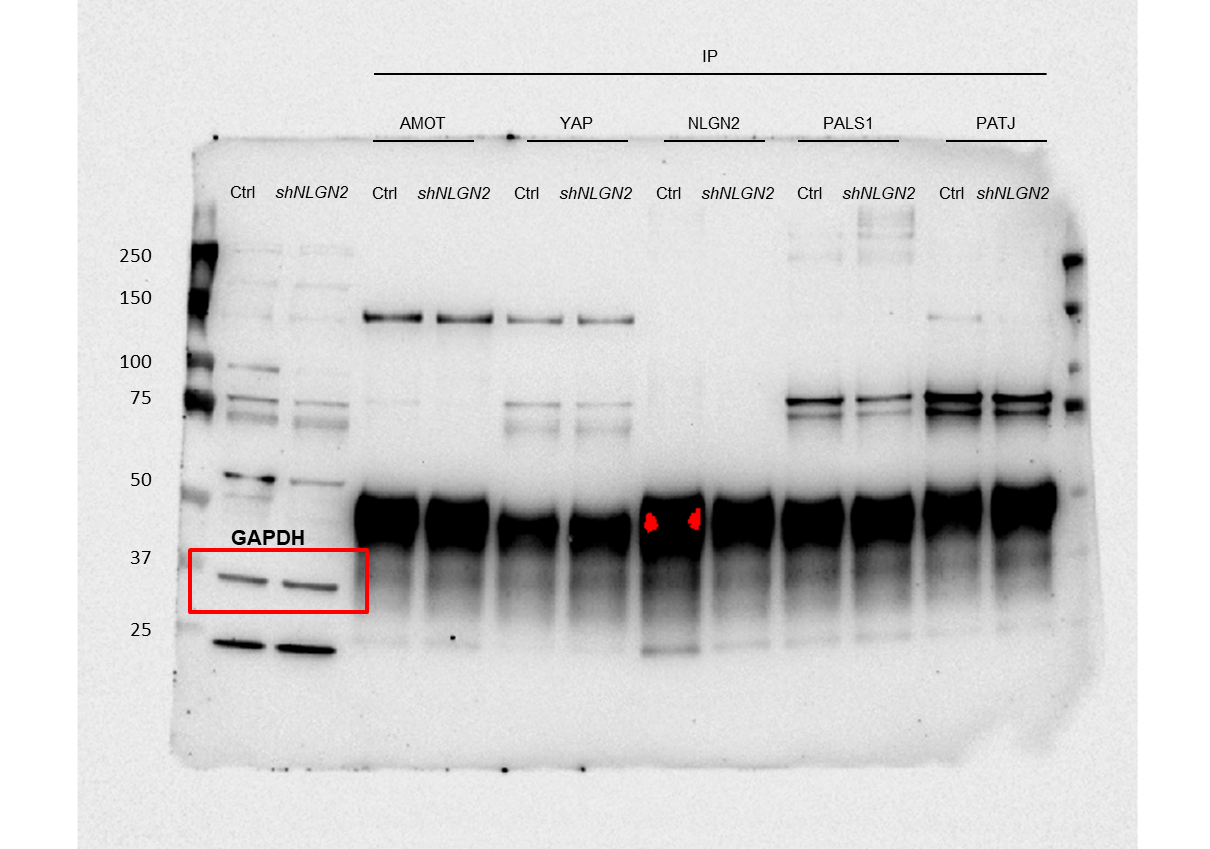

Supplement: Supplementary file 5 — Source Data Fig. 5 [file 44319_2024_104_MOESM5_ESM.zip › Figure 5/5A/WB GAPDH.tif]

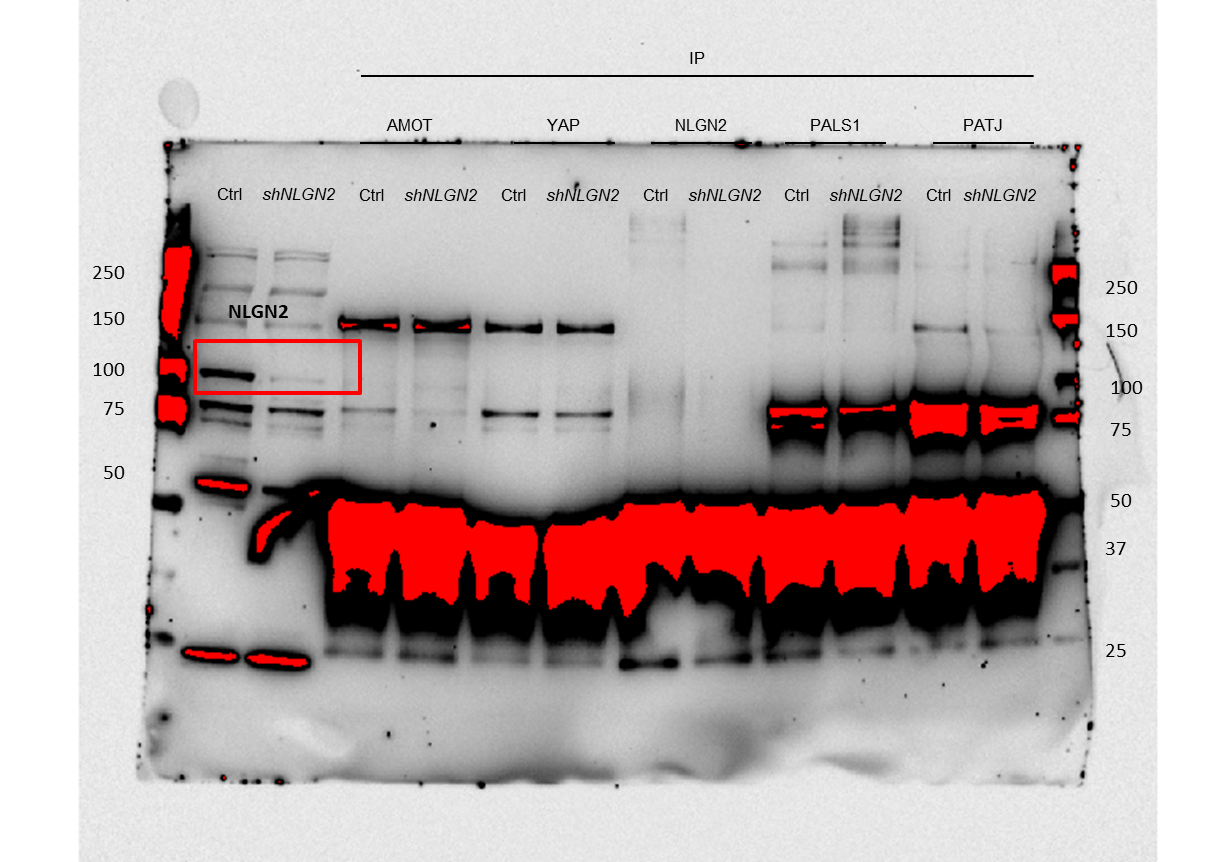

Supplement: Supplementary file 5 — Source Data Fig. 5 [file 44319_2024_104_MOESM5_ESM.zip › Figure 5/5A/WB NLGN2.tif]

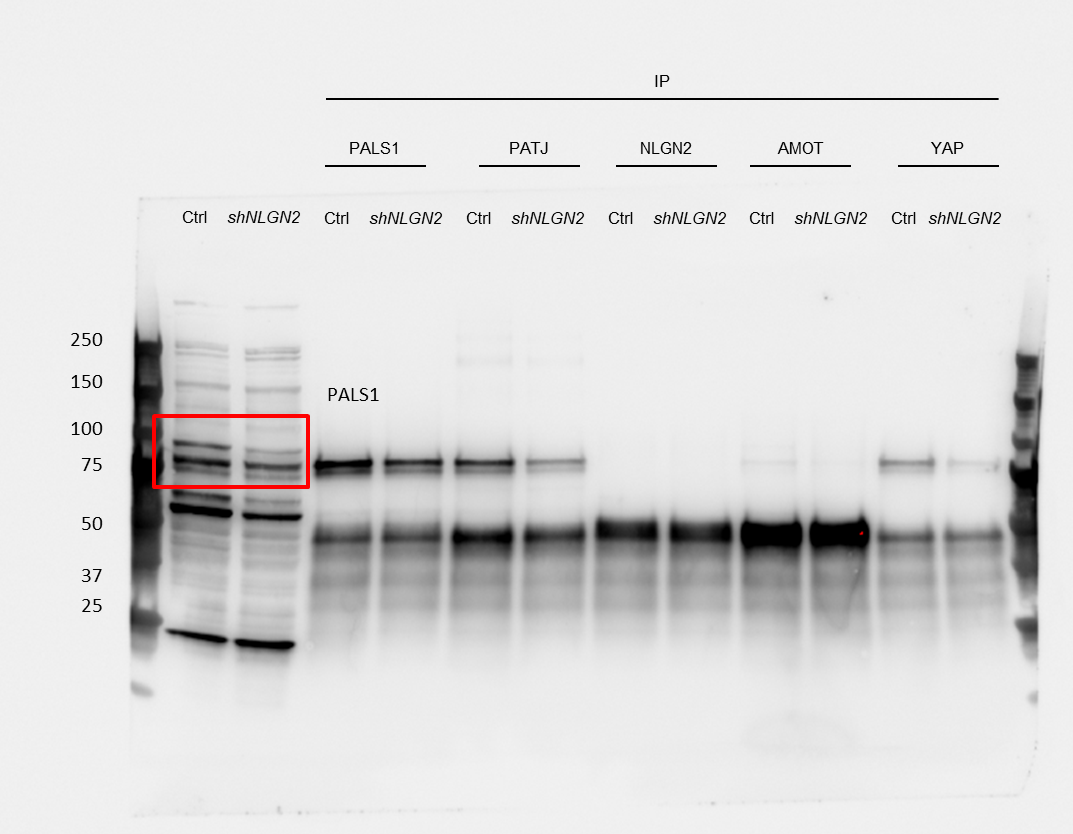

Supplement: Supplementary file 5 — Source Data Fig. 5 [file 44319_2024_104_MOESM5_ESM.zip › Figure 5/5A/WB PALS1.tif]

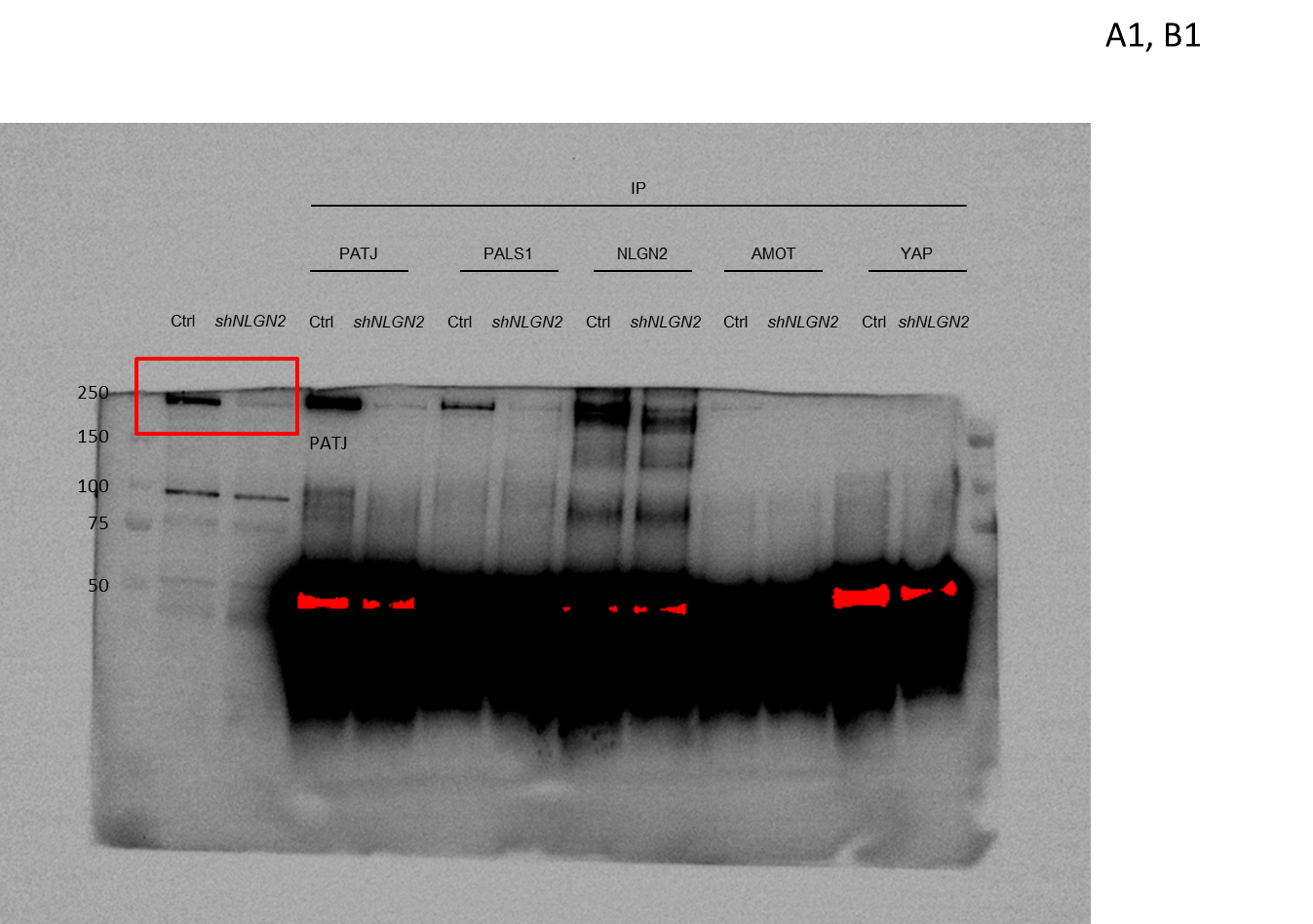

Supplement: Supplementary file 5 — Source Data Fig. 5 [file 44319_2024_104_MOESM5_ESM.zip › Figure 5/5A/WB PATJ.tif]

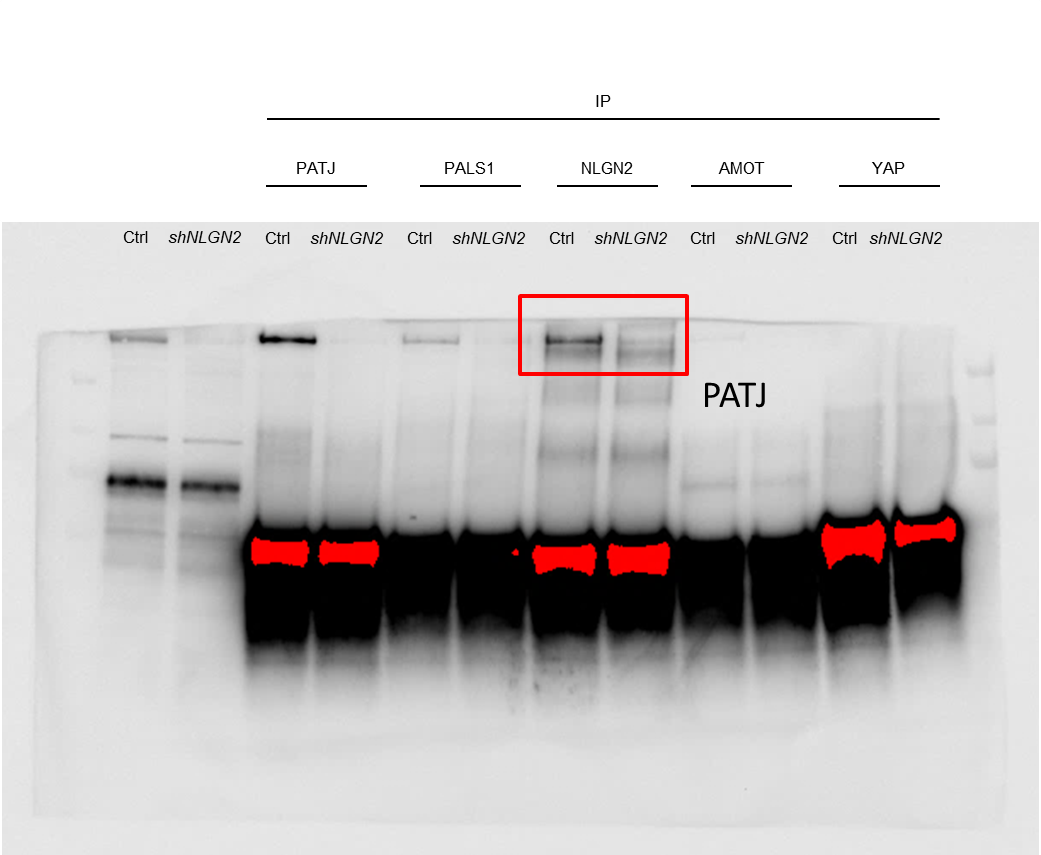

Supplement: Supplementary file 5 — Source Data Fig. 5 [file 44319_2024_104_MOESM5_ESM.zip › Figure 5/5B/WB PATJ.tif]

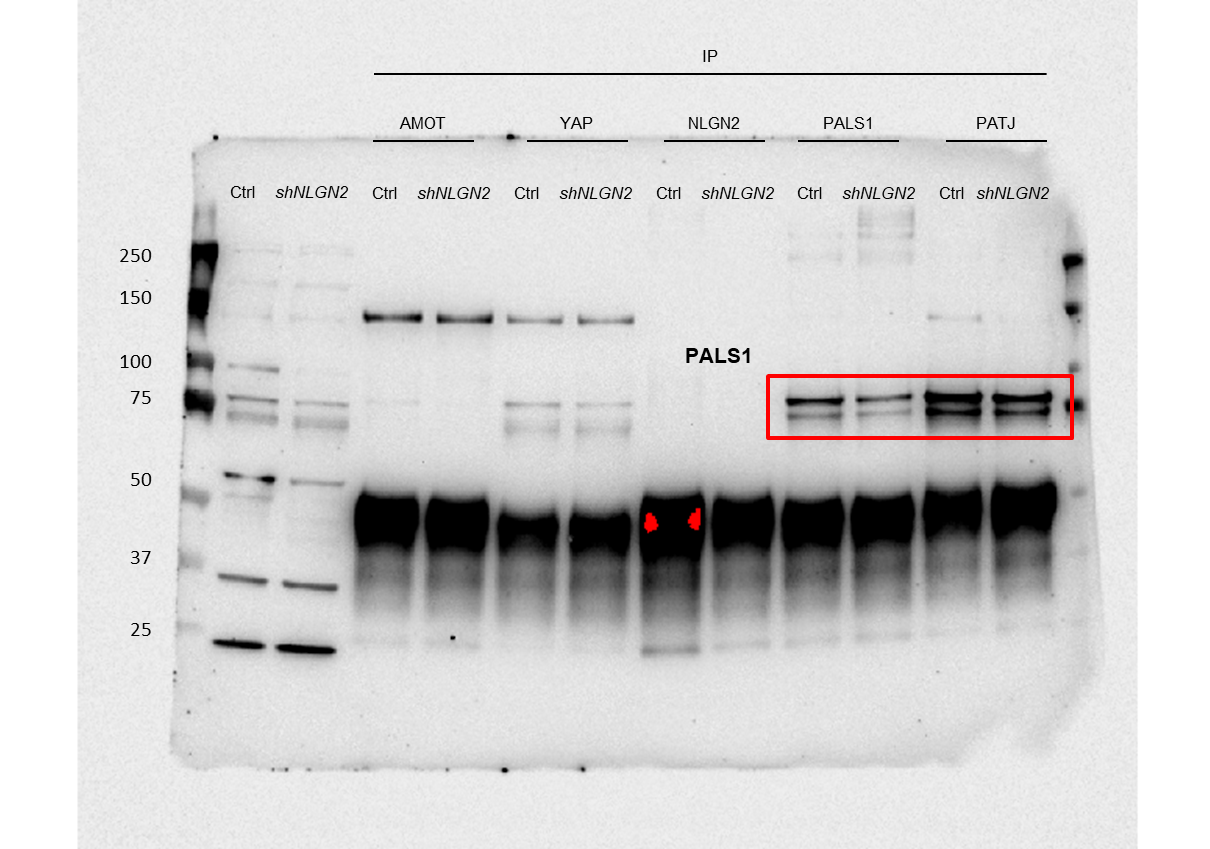

Supplement: Supplementary file 5 — Source Data Fig. 5 [file 44319_2024_104_MOESM5_ESM.zip › Figure 5/5C/Replicate/WB PALS1 REPLICATE.tif]

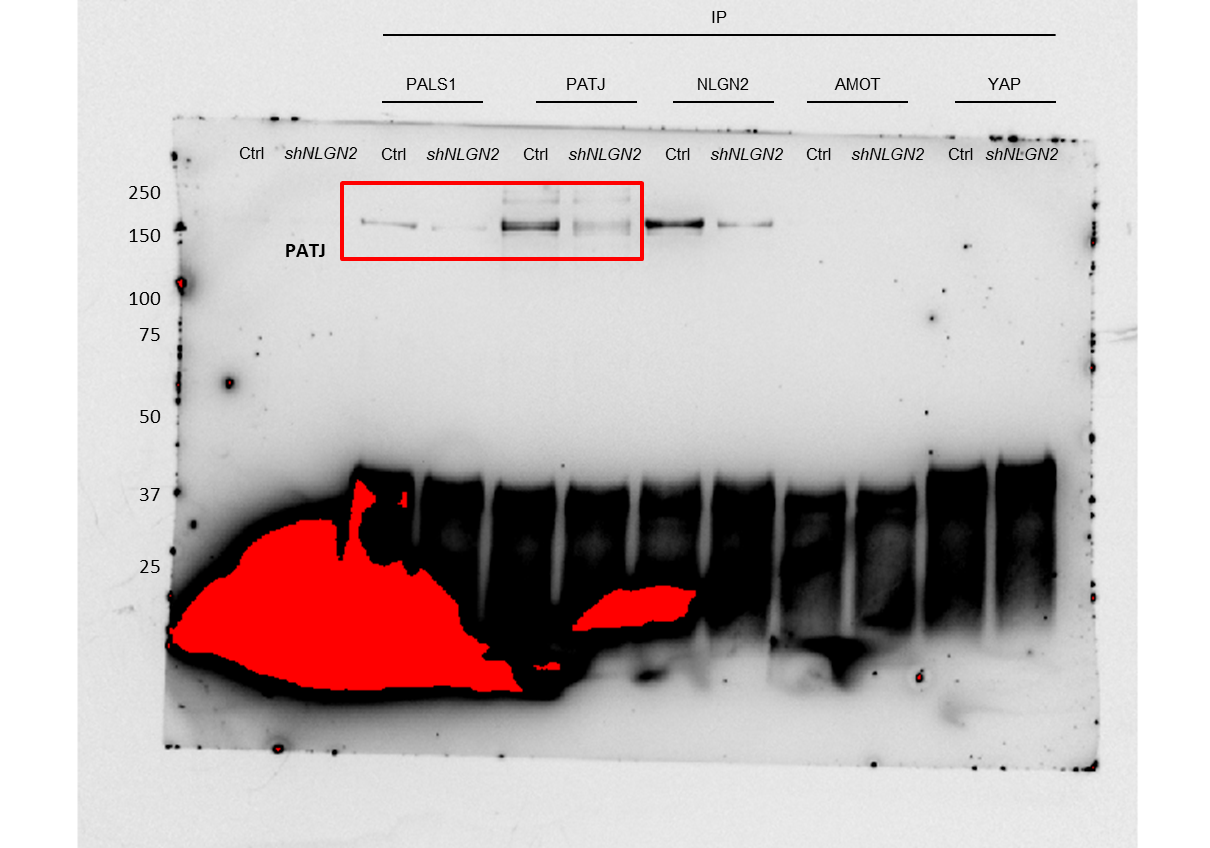

Supplement: Supplementary file 5 — Source Data Fig. 5 [file 44319_2024_104_MOESM5_ESM.zip › Figure 5/5C/Replicate/WB PATJ REPLICATE.tif]

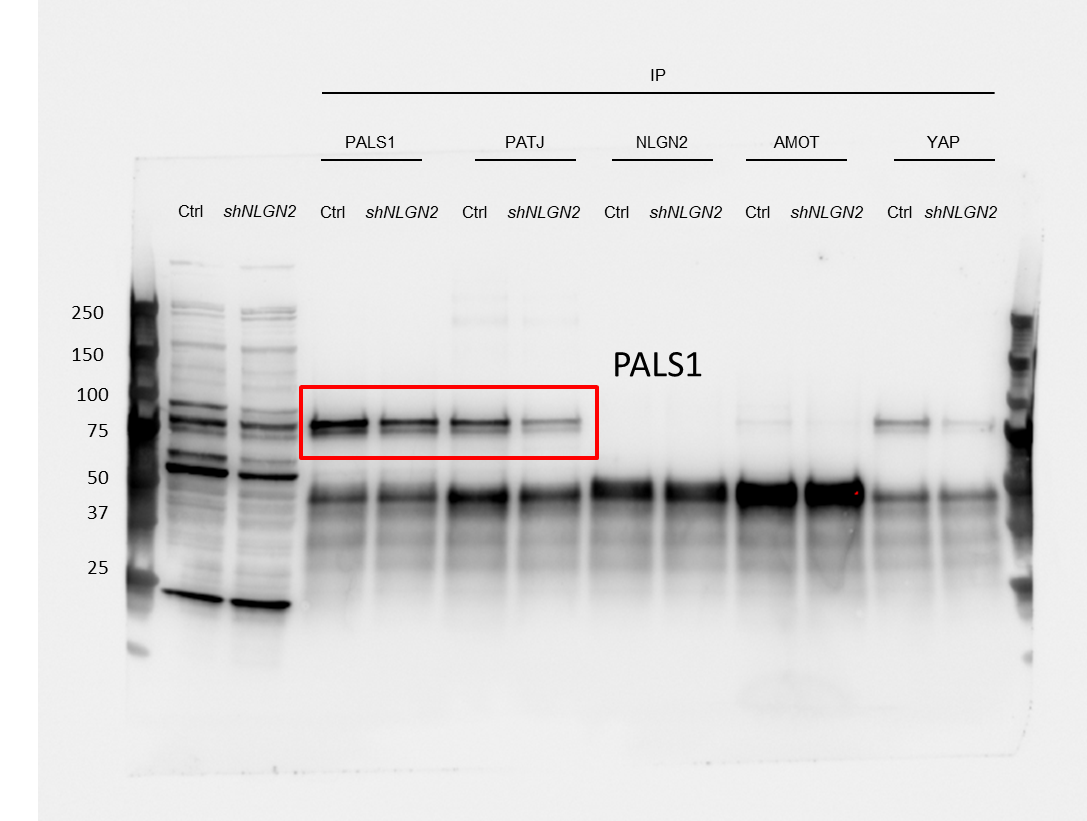

Supplement: Supplementary file 5 — Source Data Fig. 5 [file 44319_2024_104_MOESM5_ESM.zip › Figure 5/5C/WB PALS1.tif]

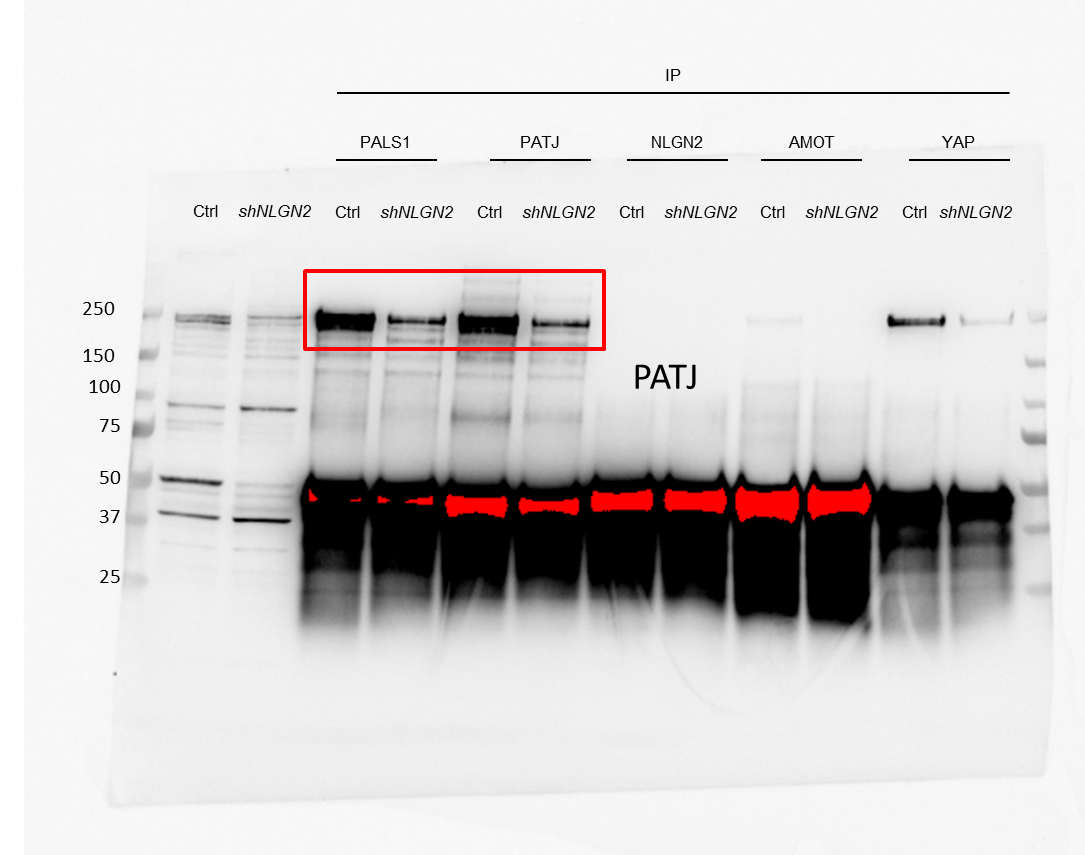

Supplement: Supplementary file 5 — Source Data Fig. 5 [file 44319_2024_104_MOESM5_ESM.zip › Figure 5/5C/WB PATJ.tif]

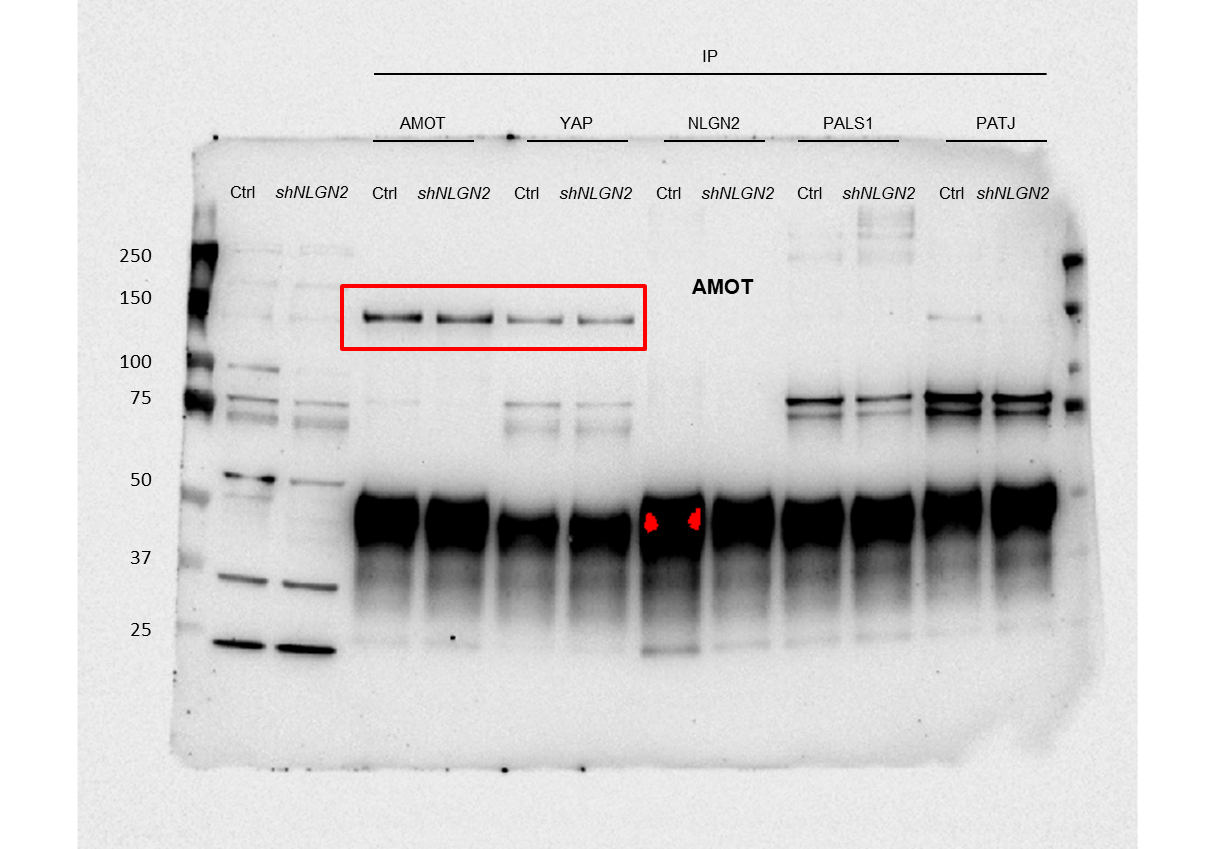

Supplement: Supplementary file 5 — Source Data Fig. 5 [file 44319_2024_104_MOESM5_ESM.zip › Figure 5/5D/Replicate/WB AMOT REPLICATE.tif]

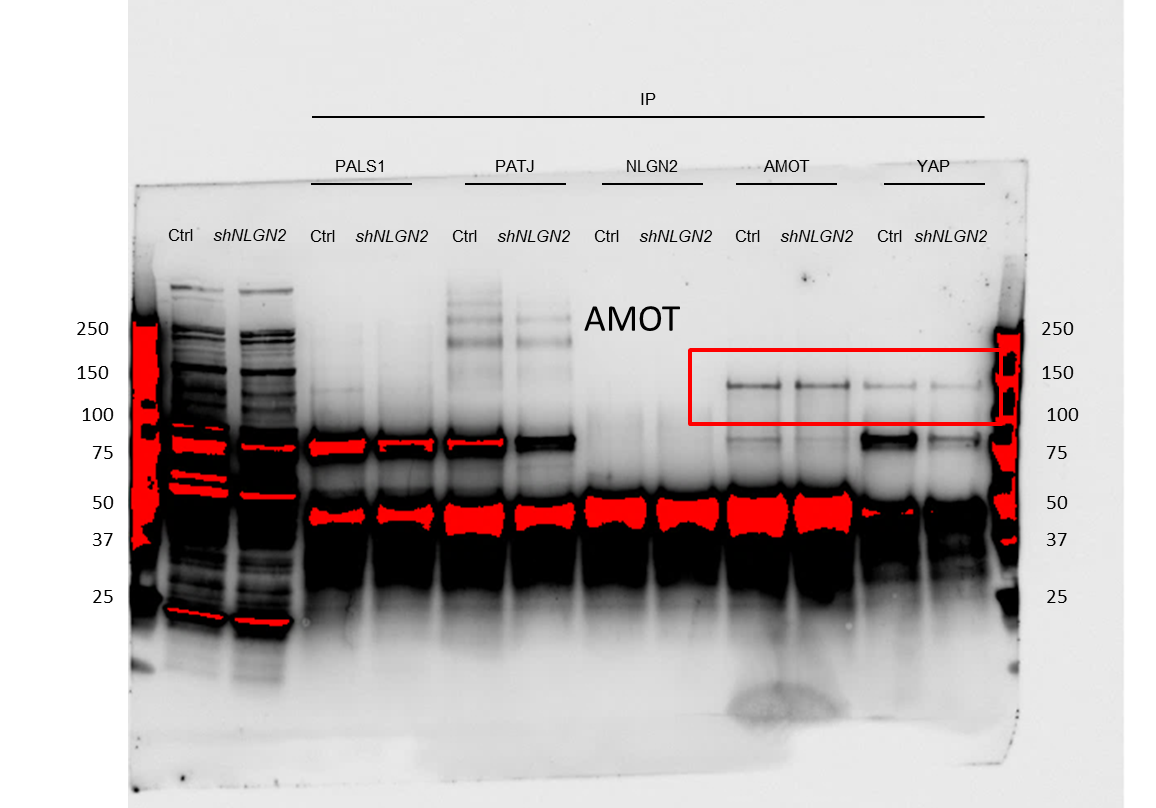

Supplement: Supplementary file 5 — Source Data Fig. 5 [file 44319_2024_104_MOESM5_ESM.zip › Figure 5/5D/WB AMOT.tif]
