# Supplementary figures and images for: A neuroligin-2-YAP axis regulates progression of pancreatic intraepithelial neoplasia (part 2 of 2)
Source: EMBO Rep. 2024 Feb 27;25(4):17. doi: 10.1038/s44319-024-00104-x (PMC11014856; doi:10.1038/s44319-024-00104-x)

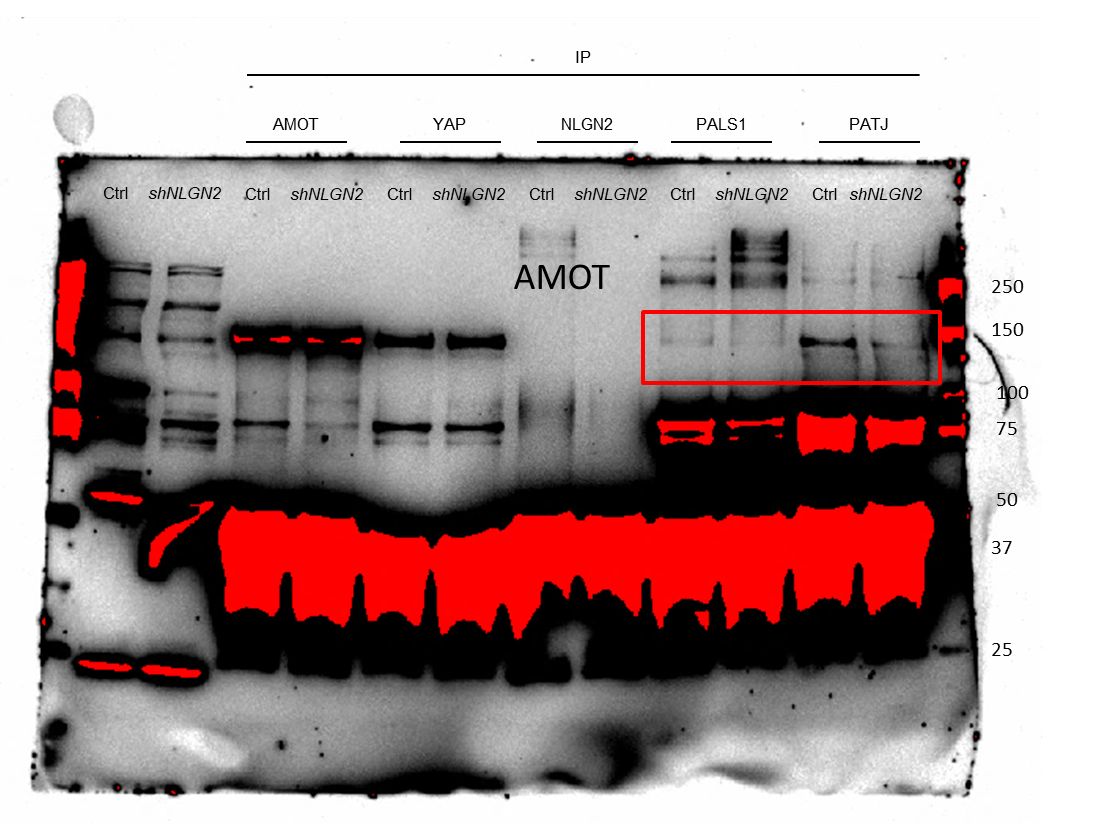

Supplement: Supplementary file 5 — Source Data Fig. 5 [file 44319_2024_104_MOESM5_ESM.zip › Figure 5/5E/WB AMOT.tif]

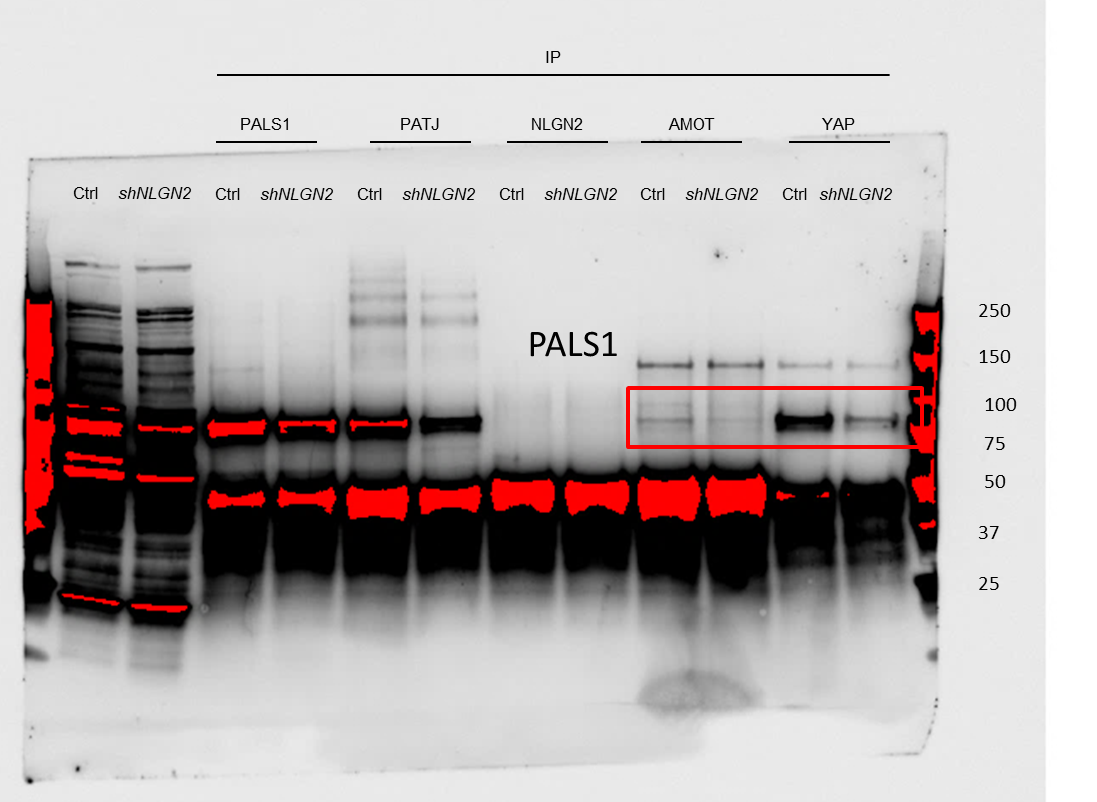

Supplement: Supplementary file 5 — Source Data Fig. 5 [file 44319_2024_104_MOESM5_ESM.zip › Figure 5/5E/WB PALS1.tif]

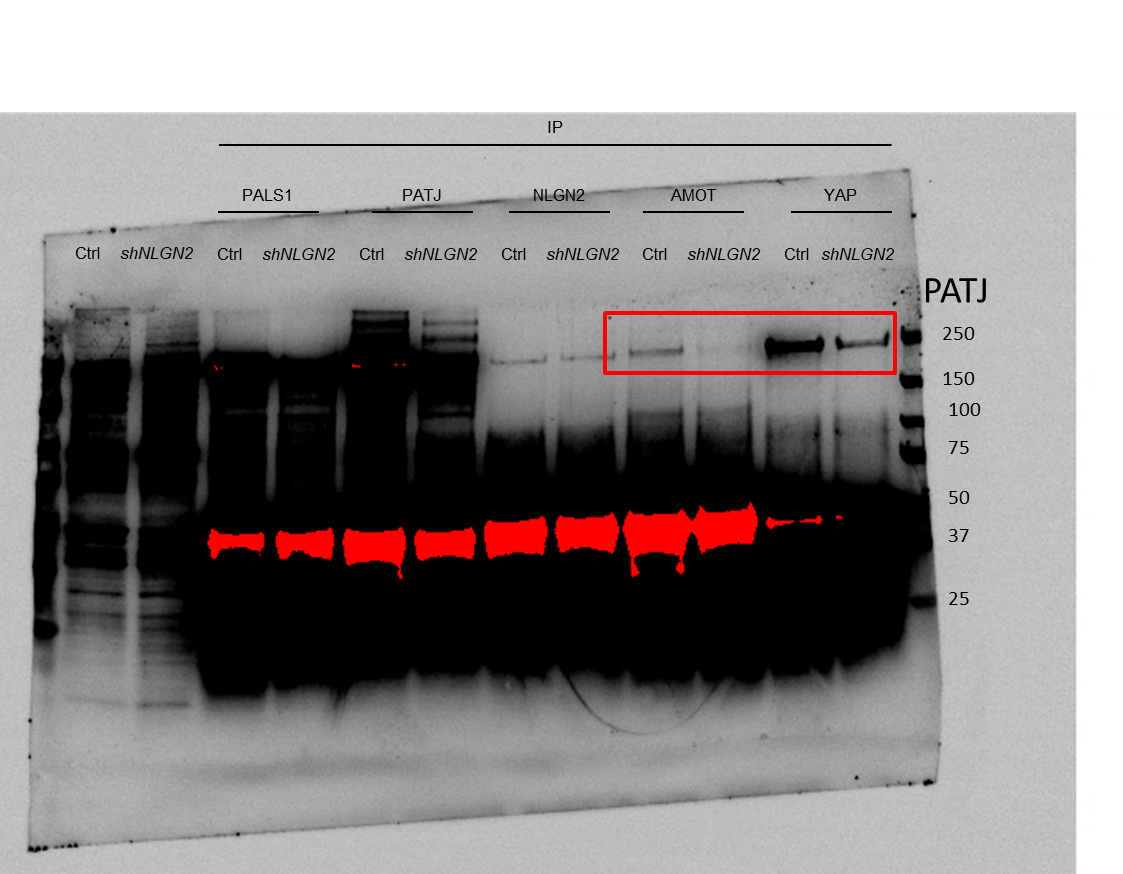

Supplement: Supplementary file 5 — Source Data Fig. 5 [file 44319_2024_104_MOESM5_ESM.zip › Figure 5/5E/WB PATJ.tif]

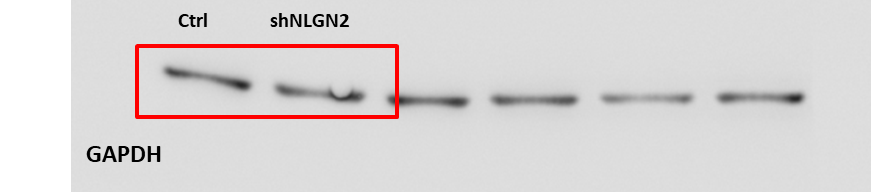

Supplement: Supplementary file 5 — Source Data Fig. 5 [file 44319_2024_104_MOESM5_ESM.zip › Figure 5/5F/WB GAPDH PYAP.tif]

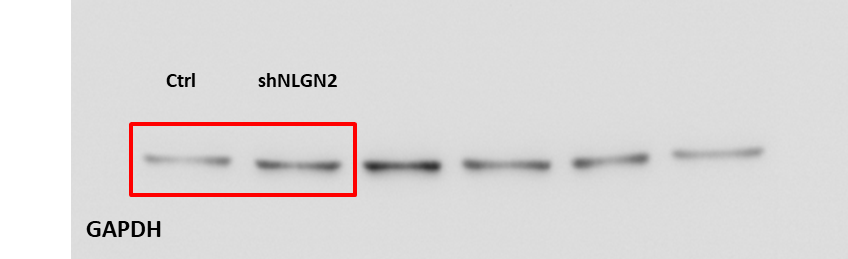

Supplement: Supplementary file 5 — Source Data Fig. 5 [file 44319_2024_104_MOESM5_ESM.zip › Figure 5/5F/WB GAPDH YAP TOT.png]

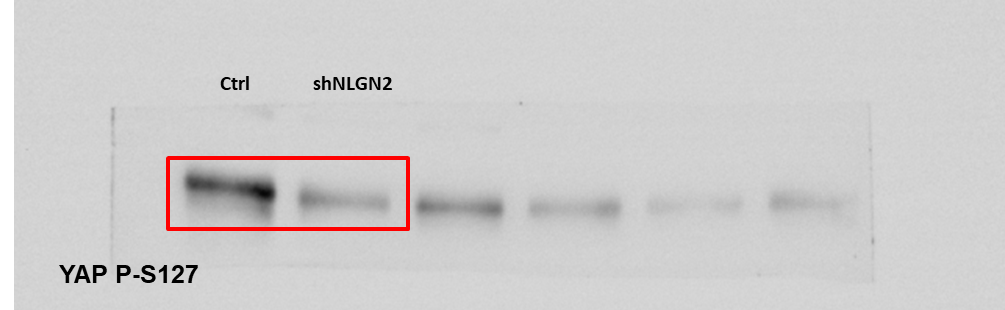

Supplement: Supplementary file 5 — Source Data Fig. 5 [file 44319_2024_104_MOESM5_ESM.zip › Figure 5/5F/WB PYAP.tif]

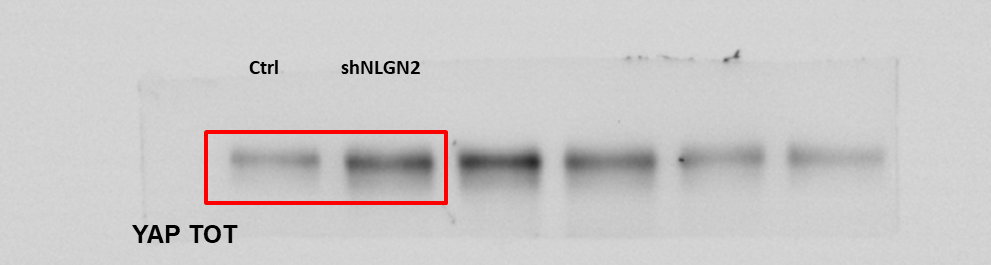

Supplement: Supplementary file 5 — Source Data Fig. 5 [file 44319_2024_104_MOESM5_ESM.zip › Figure 5/5F/WB YAP TOT.tif]

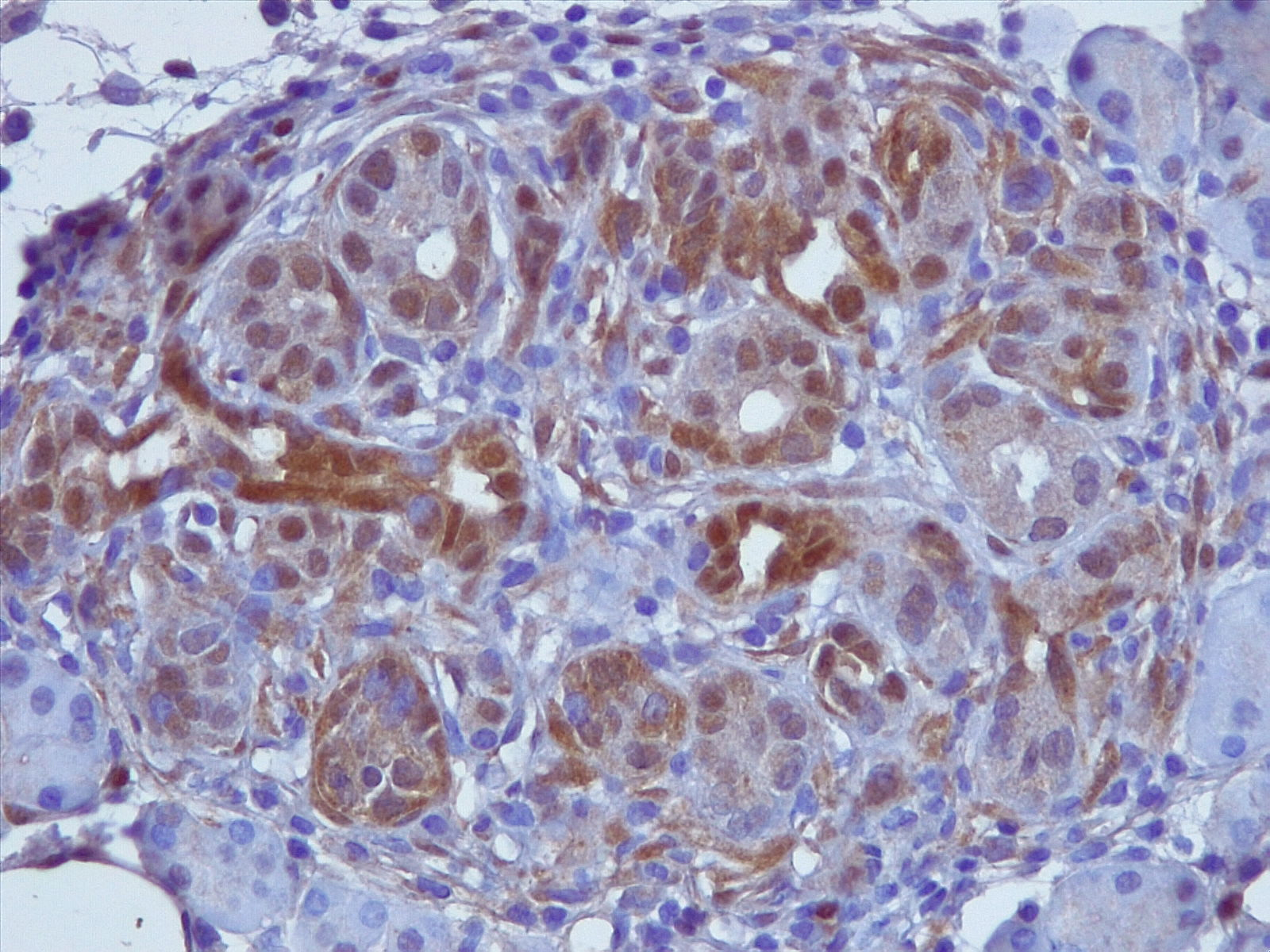

Supplement: Supplementary file 5 — Source Data Fig. 5 [file 44319_2024_104_MOESM5_ESM.zip › Figure 5/5H/ADM.jpg]

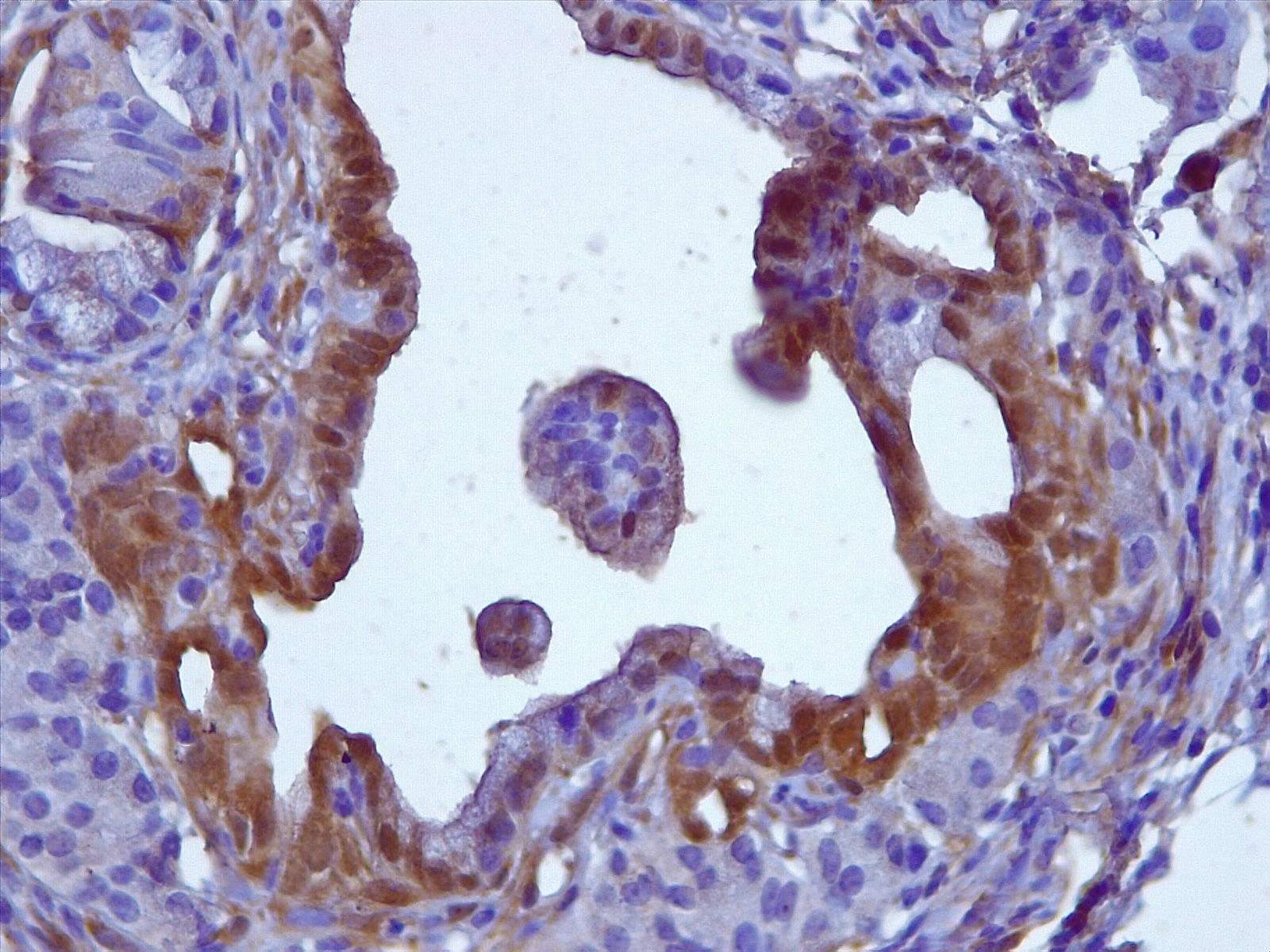

Supplement: Supplementary file 5 — Source Data Fig. 5 [file 44319_2024_104_MOESM5_ESM.zip › Figure 5/5H/High grade.jpg]

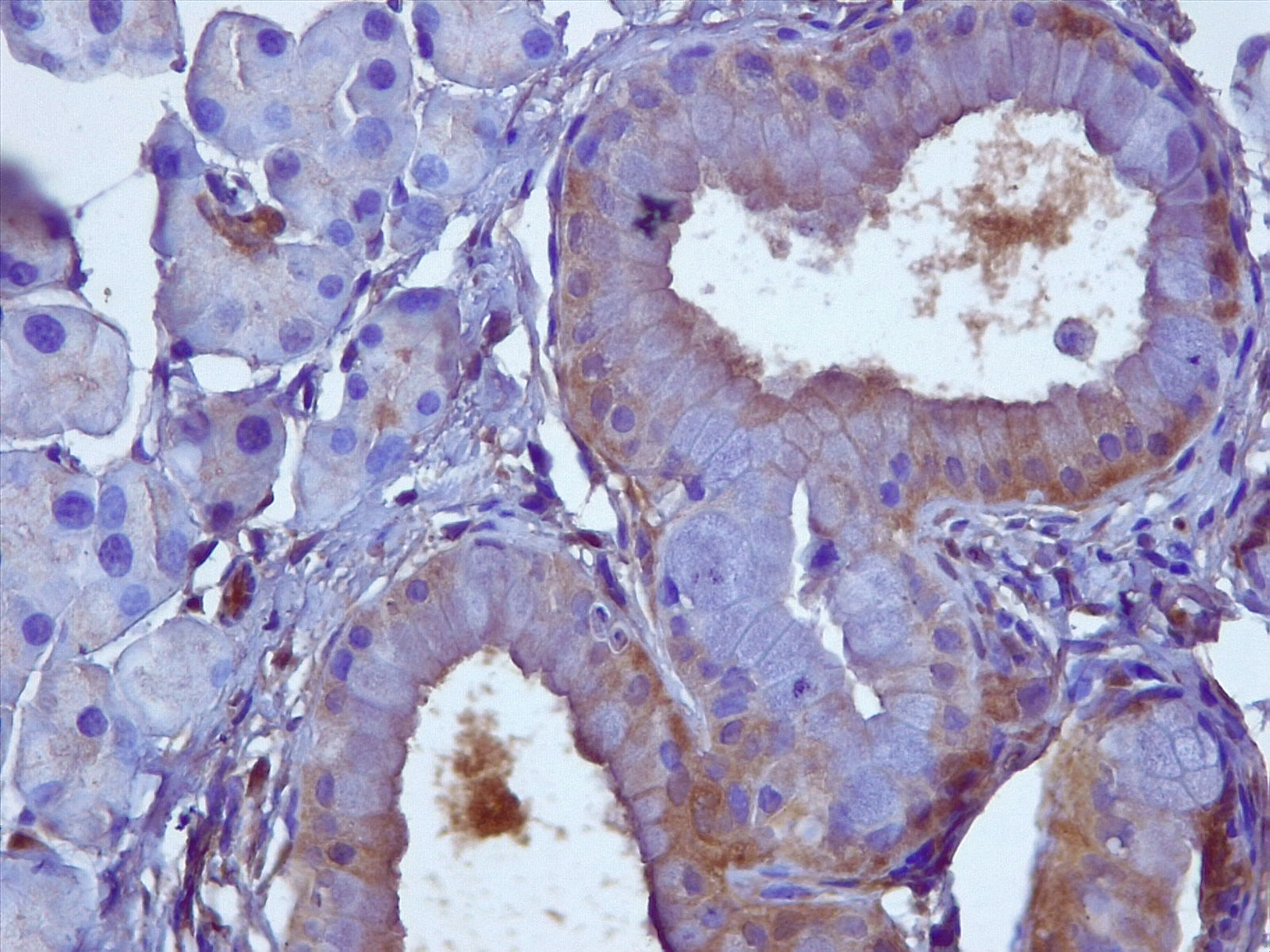

Supplement: Supplementary file 5 — Source Data Fig. 5 [file 44319_2024_104_MOESM5_ESM.zip › Figure 5/5H/Low grade.jpg]

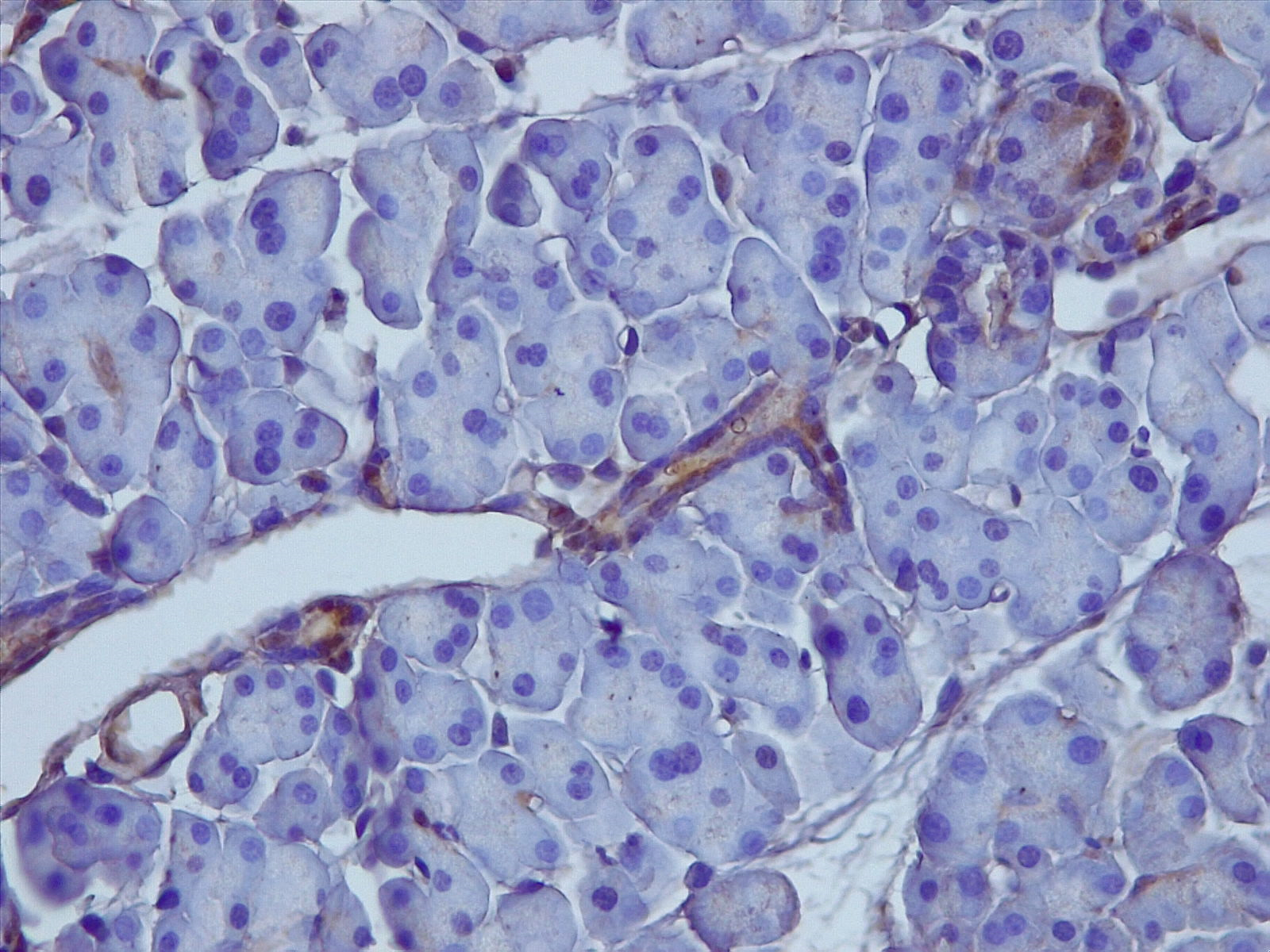

Supplement: Supplementary file 5 — Source Data Fig. 5 [file 44319_2024_104_MOESM5_ESM.zip › Figure 5/5H/Normal.jpg]

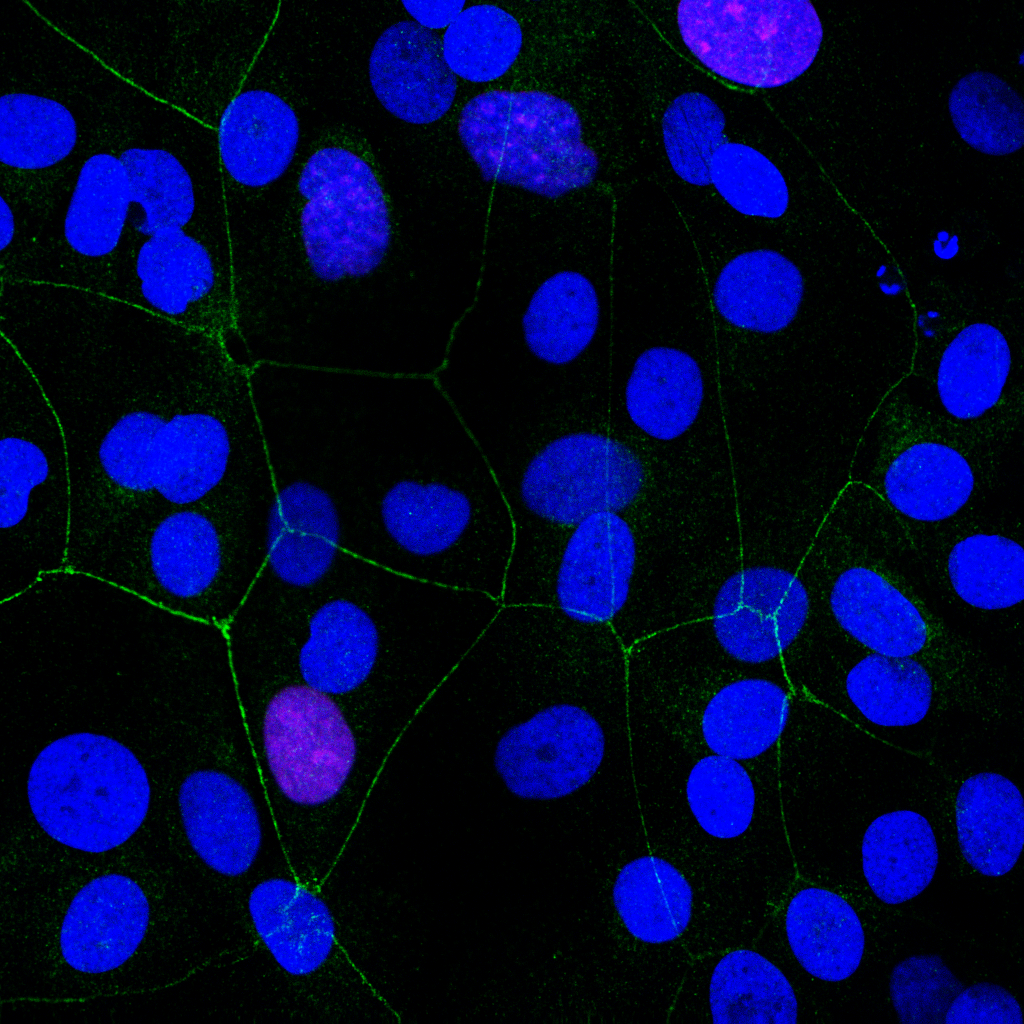

Supplement: Supplementary file 5 — Source Data Fig. 5 [file 44319_2024_104_MOESM5_ESM.zip › Figure 5/5I/shNLGN2 monolayer.tif]

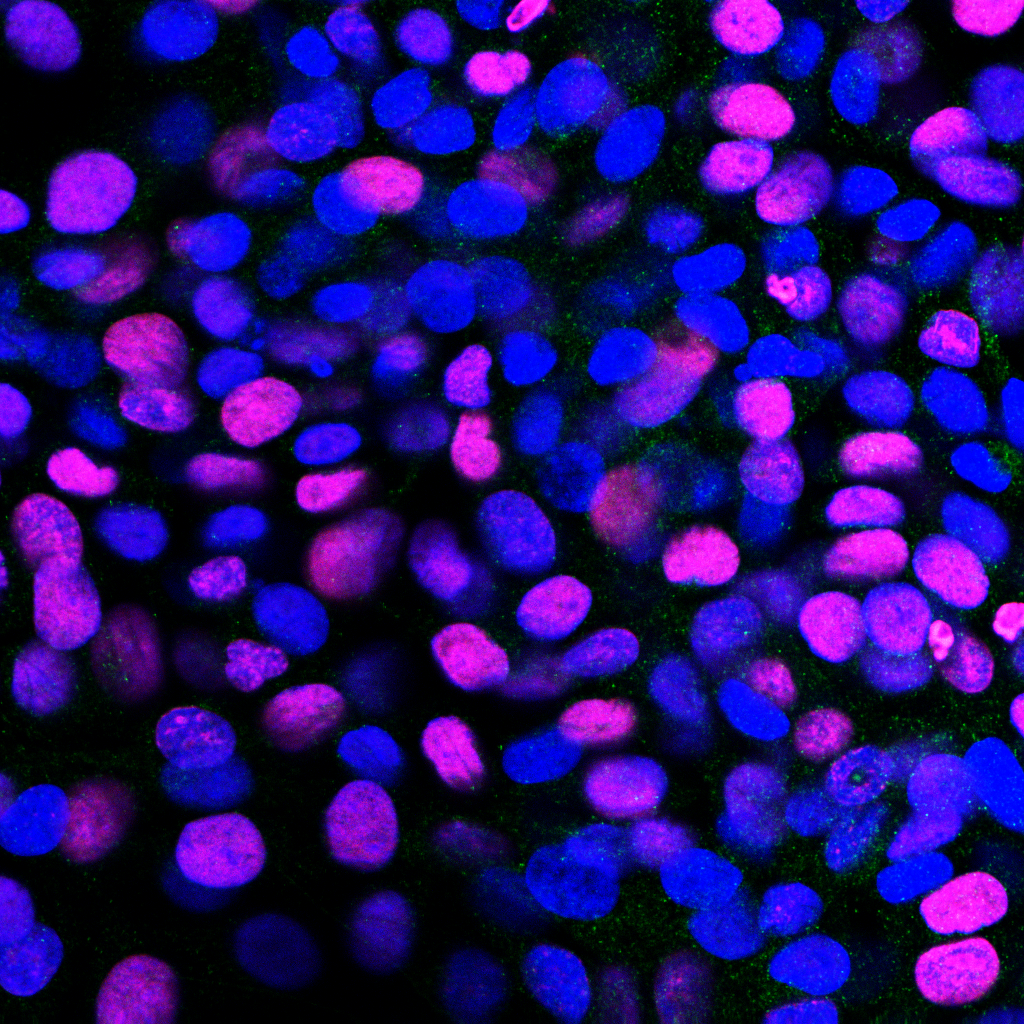

Supplement: Supplementary file 5 — Source Data Fig. 5 [file 44319_2024_104_MOESM5_ESM.zip › Figure 5/5I/shNLGN2 multilayer.tif]

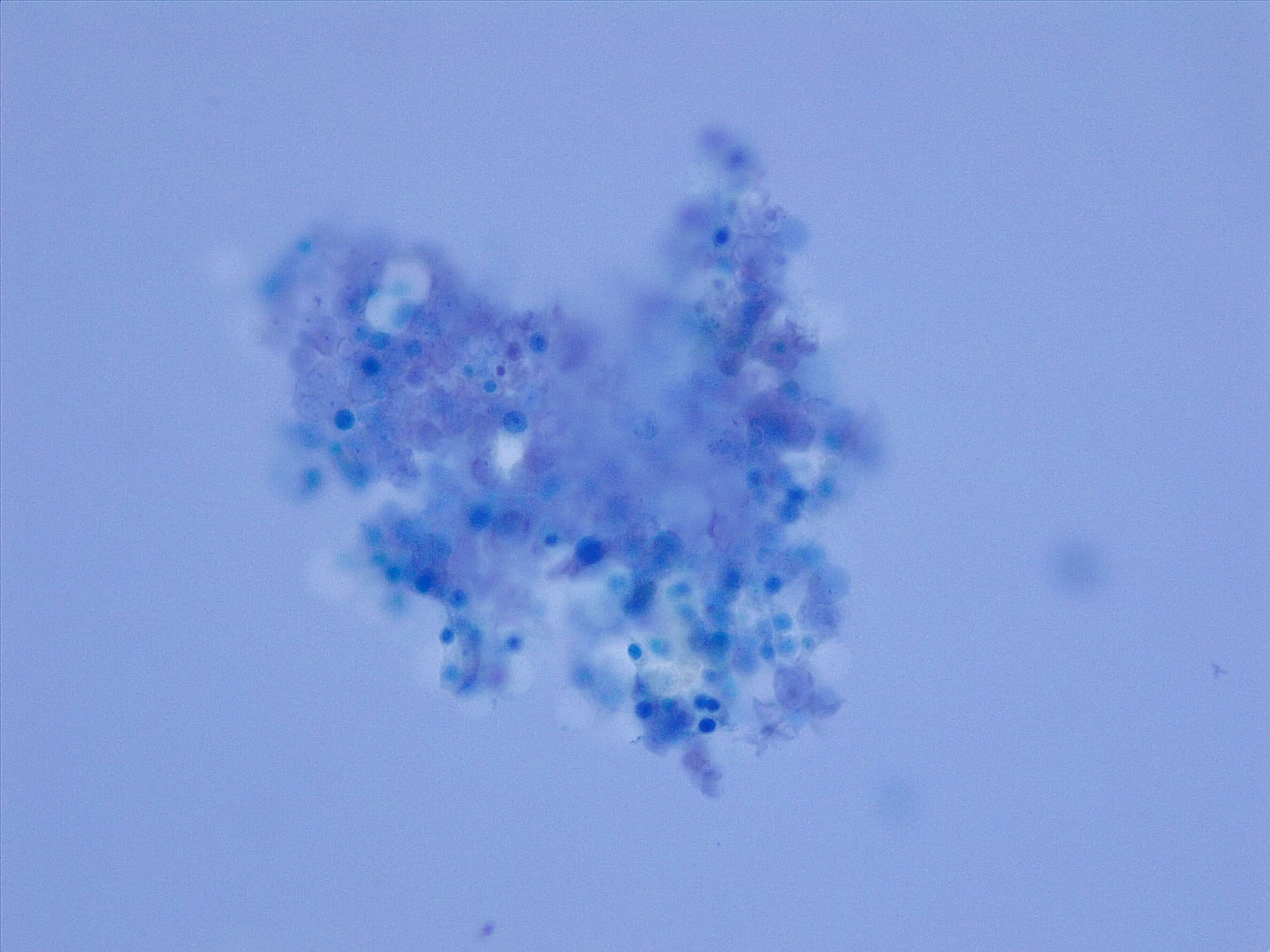

Supplement: Supplementary file 5 — Source Data Fig. 5 [file 44319_2024_104_MOESM5_ESM.zip › Figure 5/5J/shNLGN2 shYAP trypan blue.jpg]

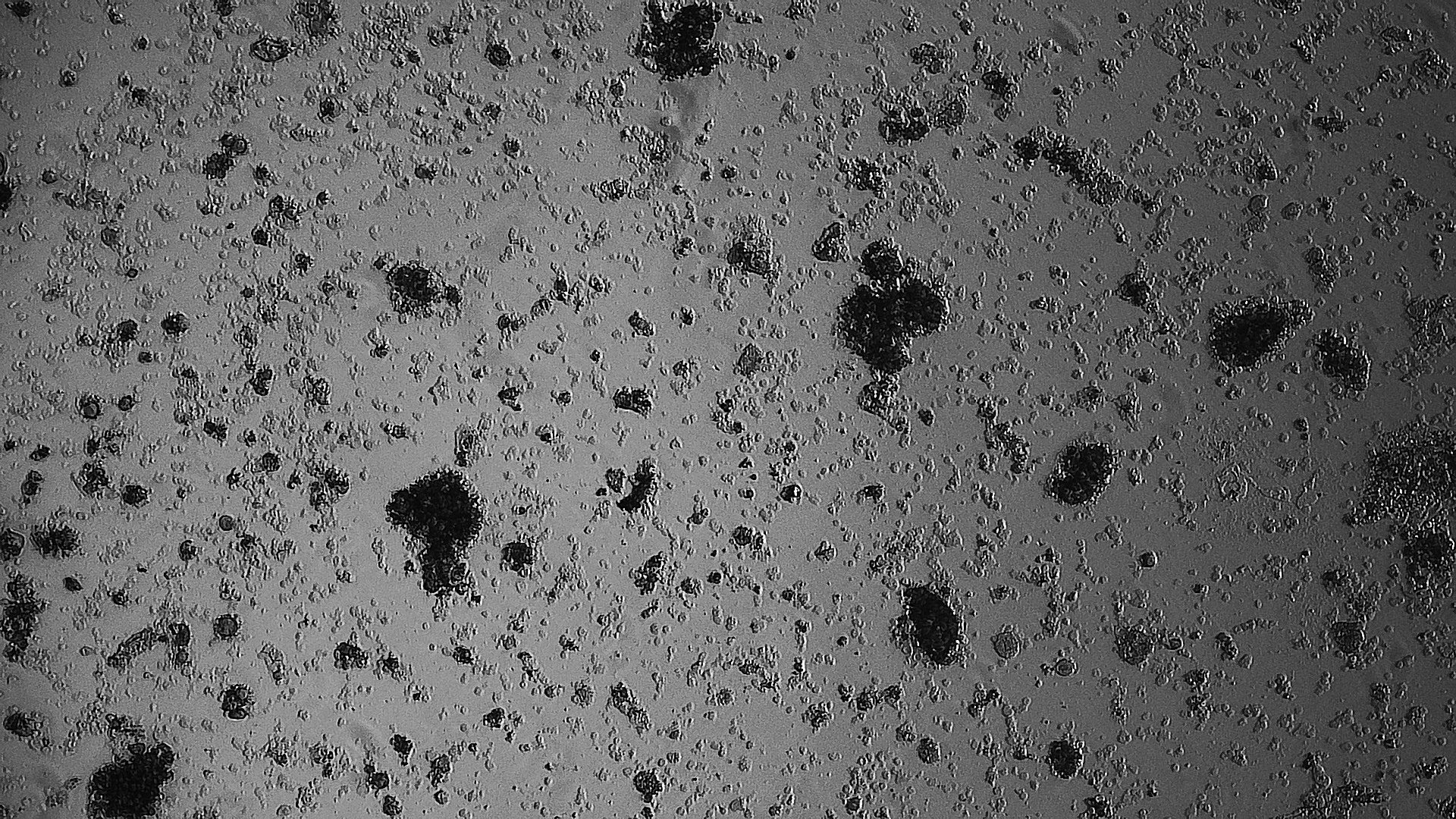

Supplement: Supplementary file 5 — Source Data Fig. 5 [file 44319_2024_104_MOESM5_ESM.zip › Figure 5/5J/shNLGN2 shYAP.tiff]

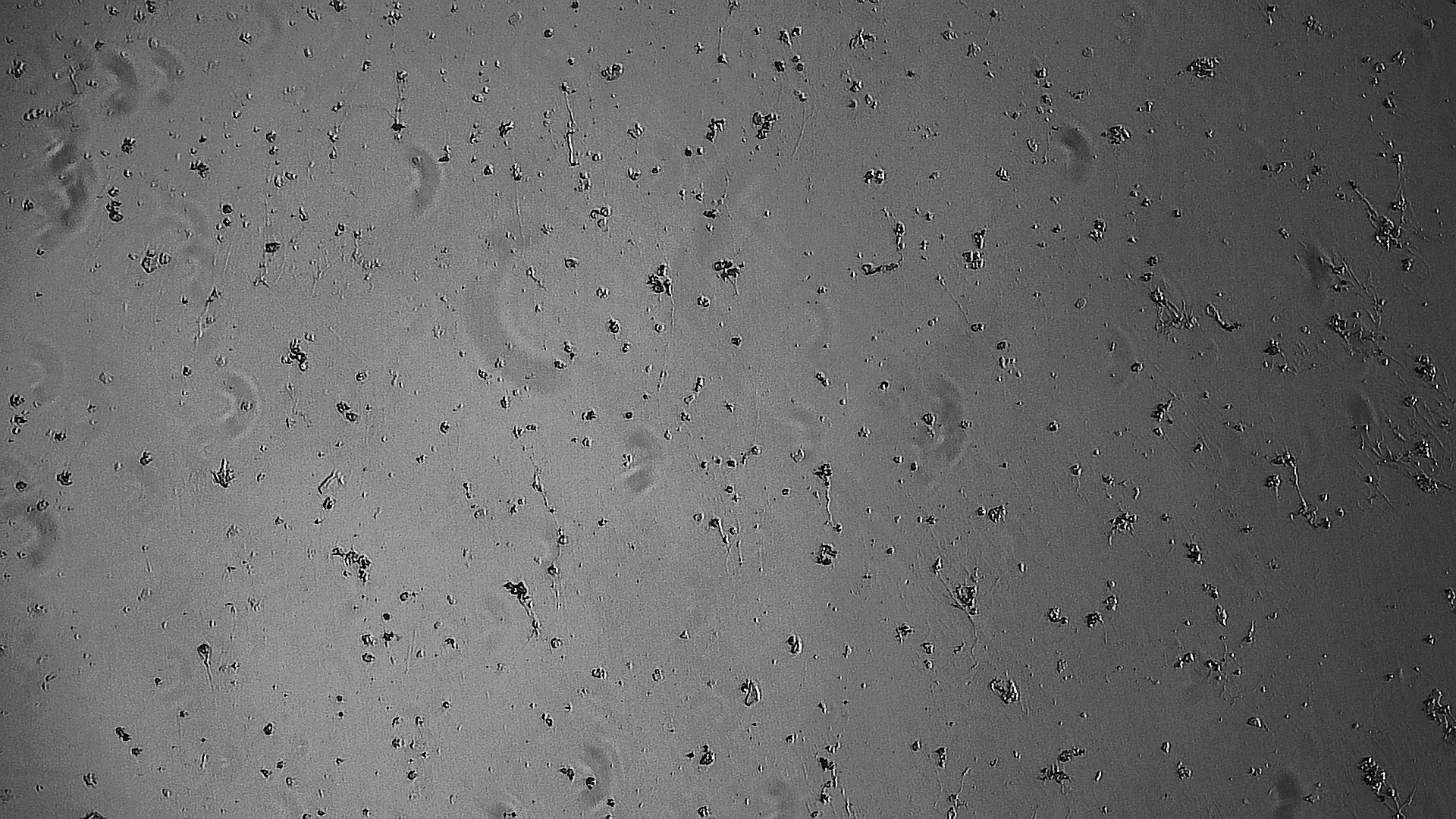

Supplement: Supplementary file 5 — Source Data Fig. 5 [file 44319_2024_104_MOESM5_ESM.zip › Figure 5/5J/shNLGN2.tiff]

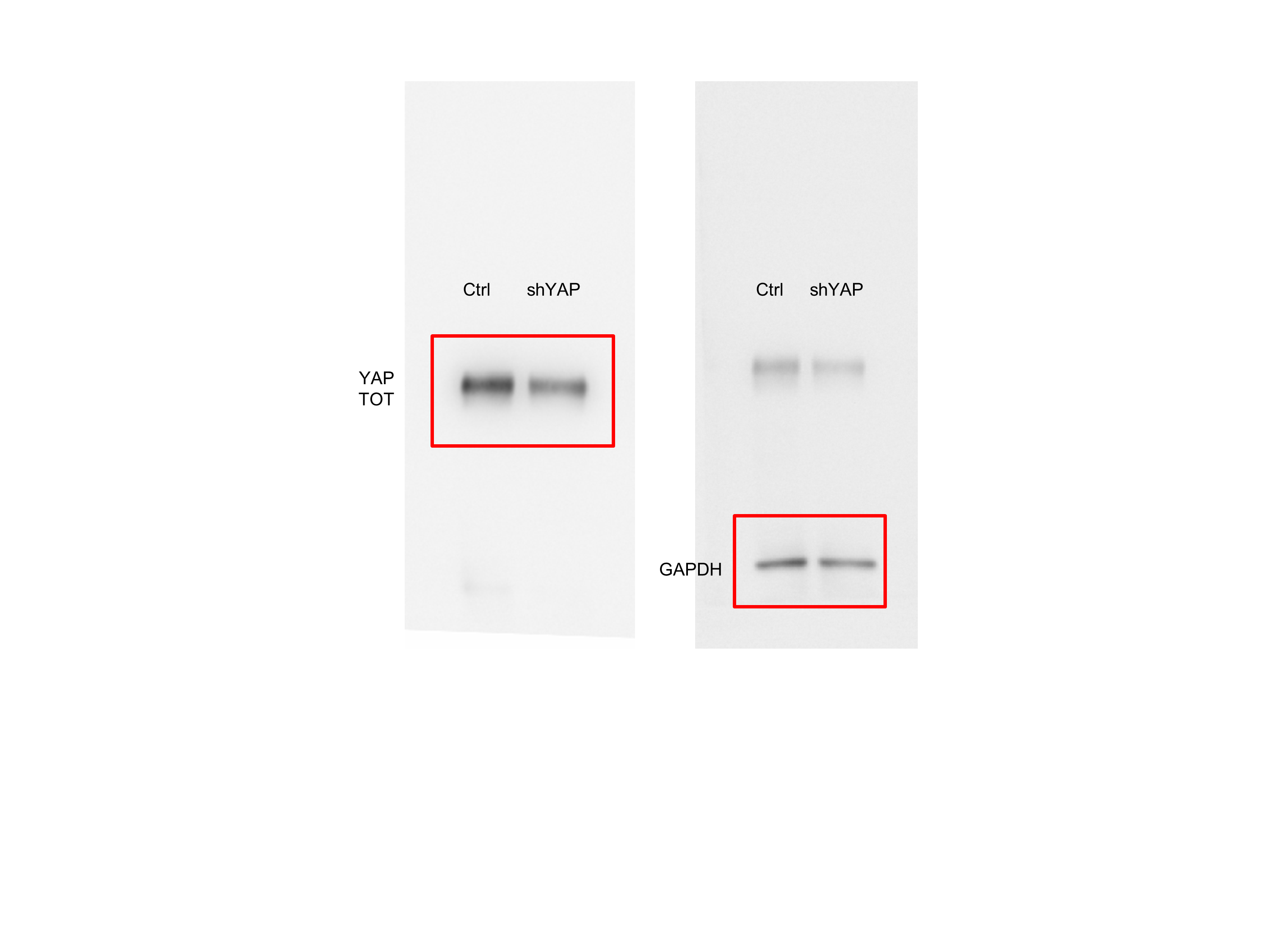

Supplement: Supplementary file 6 — EV Figures [file 44319_2024_104_MOESM6_ESM.zip › EV5.TIF]

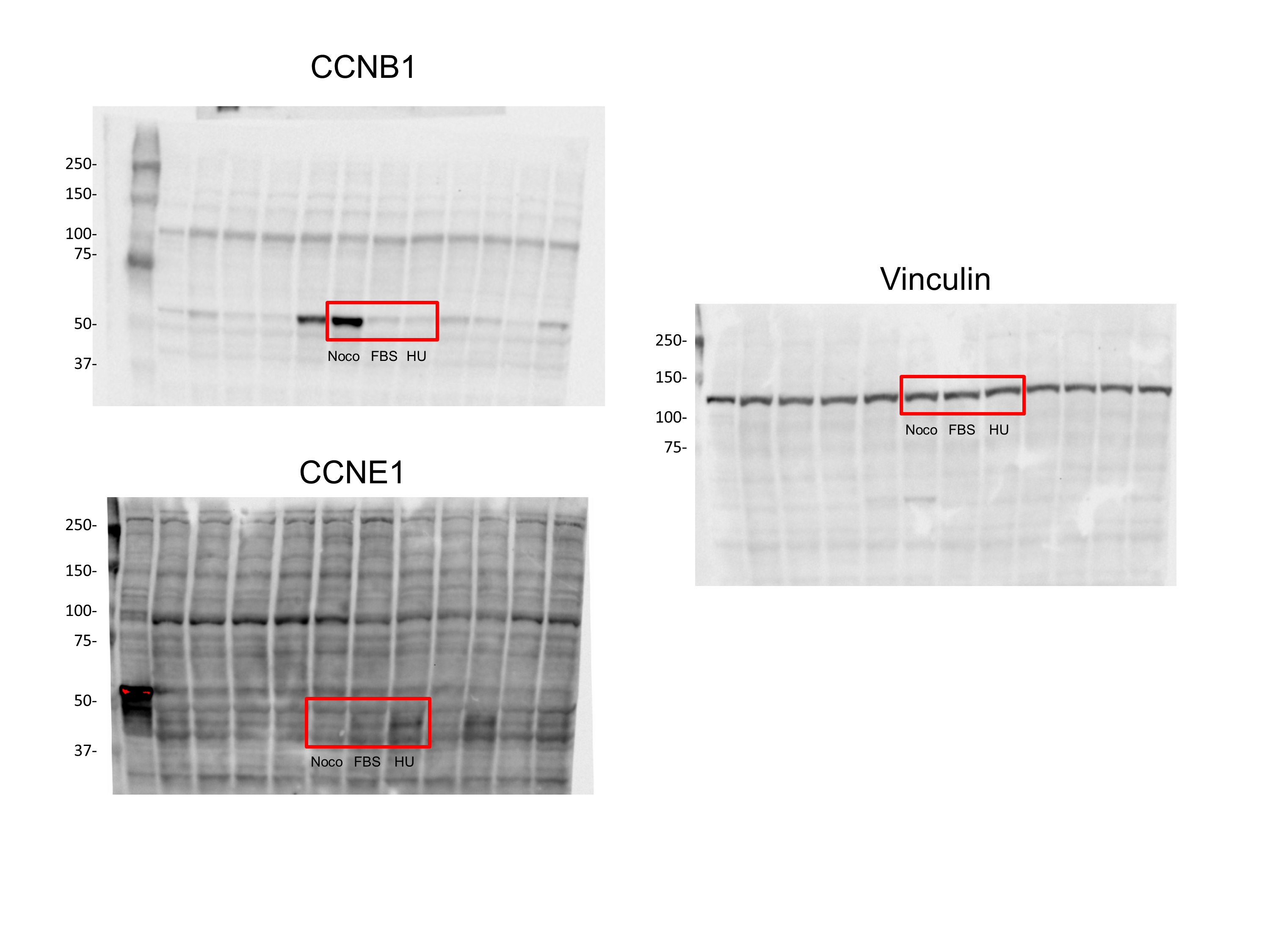

Supplement: Supplementary file 6 — EV Figures [file 44319_2024_104_MOESM6_ESM.zip › EV3.TIF]

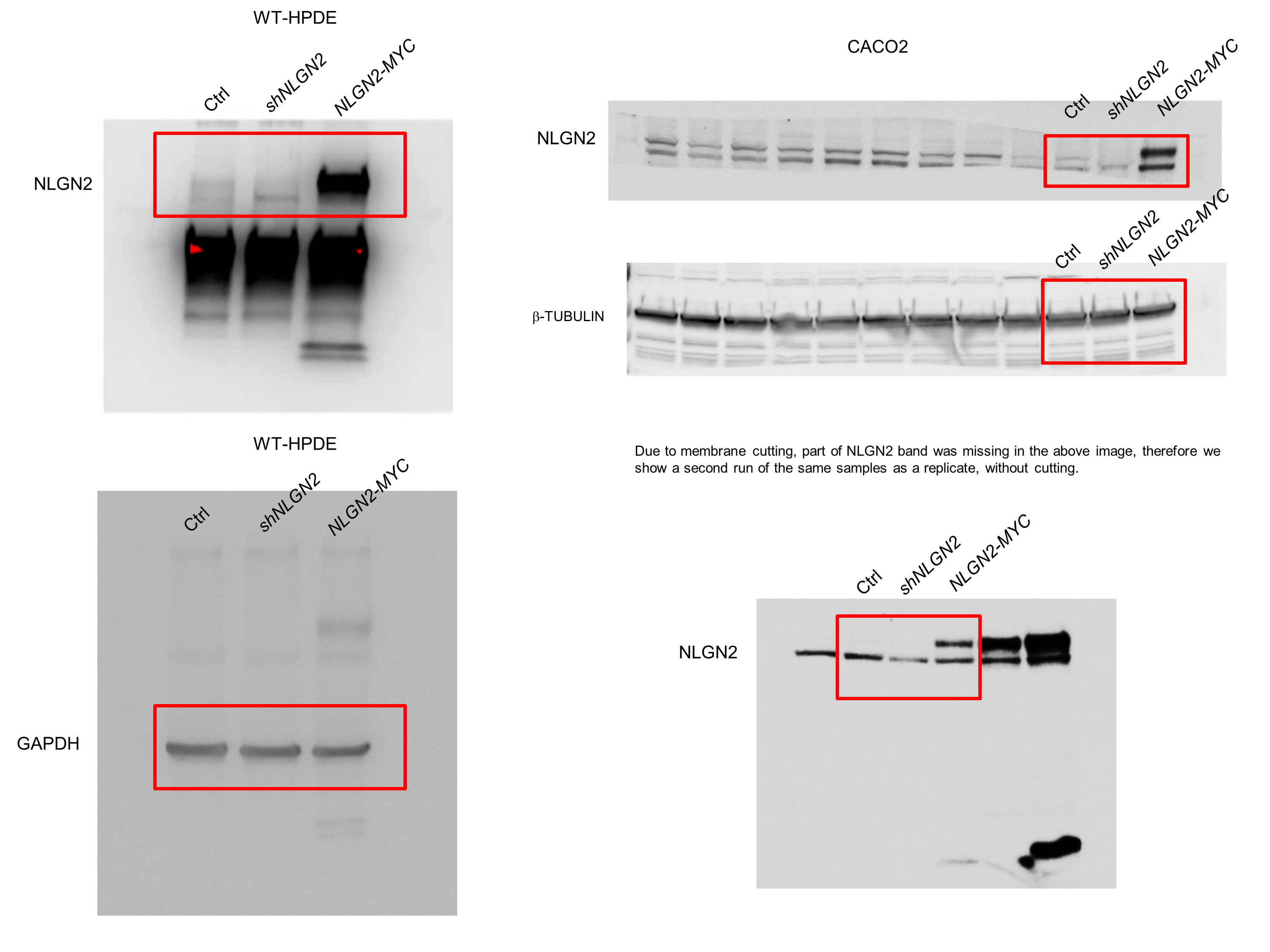

Supplement: Supplementary file 6 — EV Figures [file 44319_2024_104_MOESM6_ESM.zip › EV4.TIF]
