# Supplementary material for: A neuroligin-2-YAP axis regulates progression of pancreatic intraepithelial neoplasia
Source: EMBO Rep. 2024 Feb 27;25(4):17. doi: 10.1038/s44319-024-00104-x (PMC11014856; doi:10.1038/s44319-024-00104-x)
Supplement: Supplementary file 8 — Expanded View Figures [file 44319_2024_104_MOESM8_ESM.pdf]

Expanded View Figures

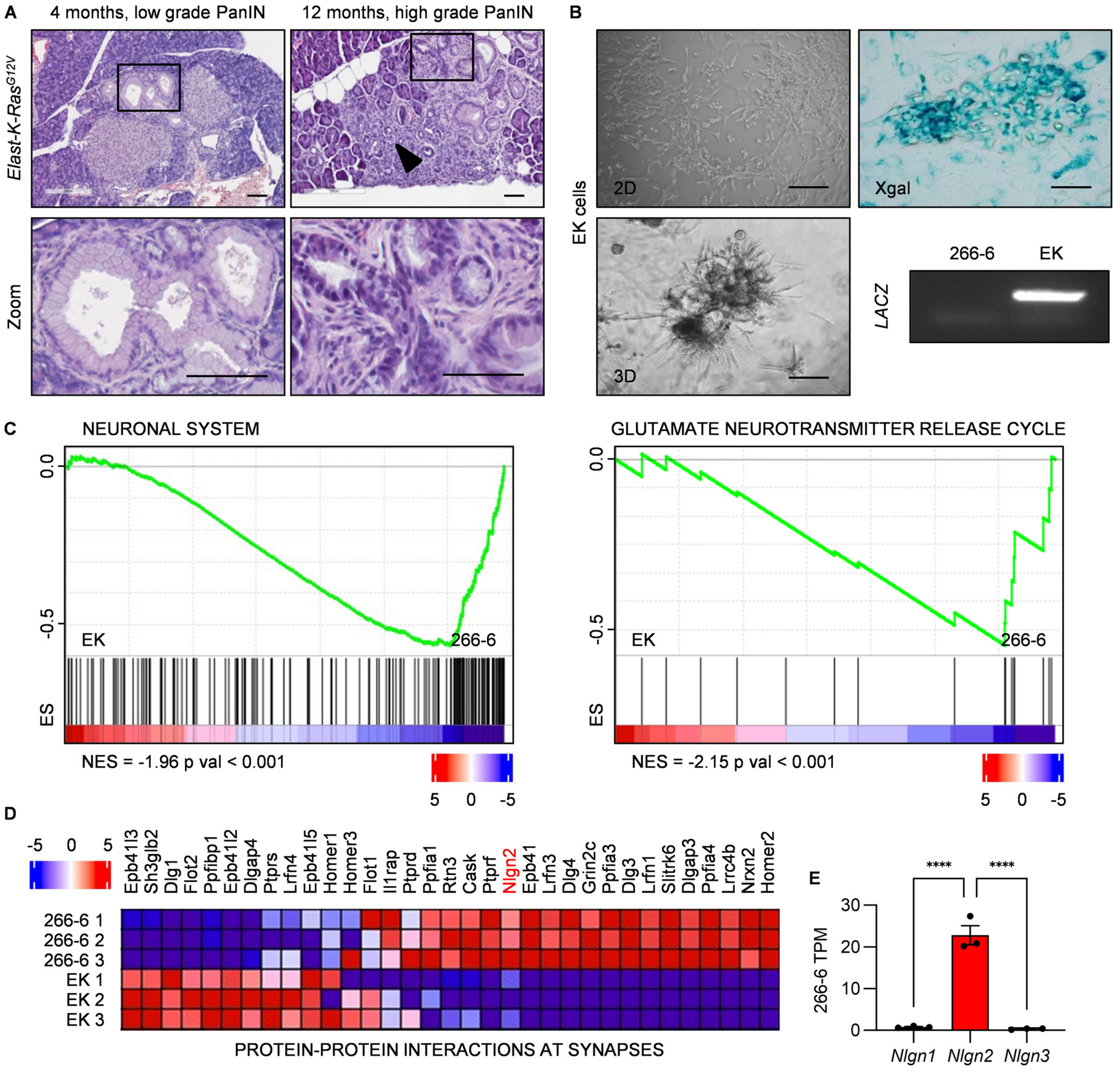

Figure EV1. Characterization of PanIN lesions in *Elast-K-Ras^{G12V}* mice and characterization of EK cells.

(**A**) *Elast-K-Ras^{G12V}* mice spontaneously develop PanIN lesions with progressive features. The upper panel indicates PanIN grade progression with mouse age. At month 12, histological signs of pancreatitis were present (arrowhead). The square boxes indicate the areas shown at higher magnification in the bottom panels. In low-grade PanIN, mucin accumulation was still evident, and in high-grade PanIN, progressive loss of polarity was observed. (**B**) Left panels: representative images of EK cells growing with a mesenchymal phenotype on a 2D plastic surface (top) and with disordered and unpolarized architecture in 3D Matrigel culture (bottom). Right panels: EK cells carry *K-Ras^{G12V}*, as demonstrated by the expression of LACZ demonstrated by X-Gal staining (top) and by PCR (bottom). (**C**) "Neuronal system" and "Glutamate neurotransmitter release cycle" gene sets enriched in 266-6 compared to EK cells. Red and blue color scale indicate $\log_2$ mRNA fold change ES: enrichment Score. NES: normalized enrichment score. (**D**) Heatmap showing the $\log_2$ mRNA fold change (red and blue scale) between EK and 266-6 cells of genes in "protein–protein interactions at synapses" gene set enriched in 266-6 according to GSEA. *Nlgn2* is highlighted in red. (**E**) TPM count of *Nlgn1*, *Nlgn2*, and *Nlgn3* transcripts from RNA seq data of 266-6 cells, showing that *Nlgn2* is the most expressed. Data information: (**A**) representative images of 12 mice, scale bar: 0.1 mm. (**B**) Scale bars: 0.05 mm. (**C**) Gene sets are identified by GSEA (Kolmogorov–Smirnov test). (**E**) The graph shows mean TPM count ± SEM (n of biological replicates = 3; unpaired t-test: ****$p < 0.0001$).

 © The Author(s)

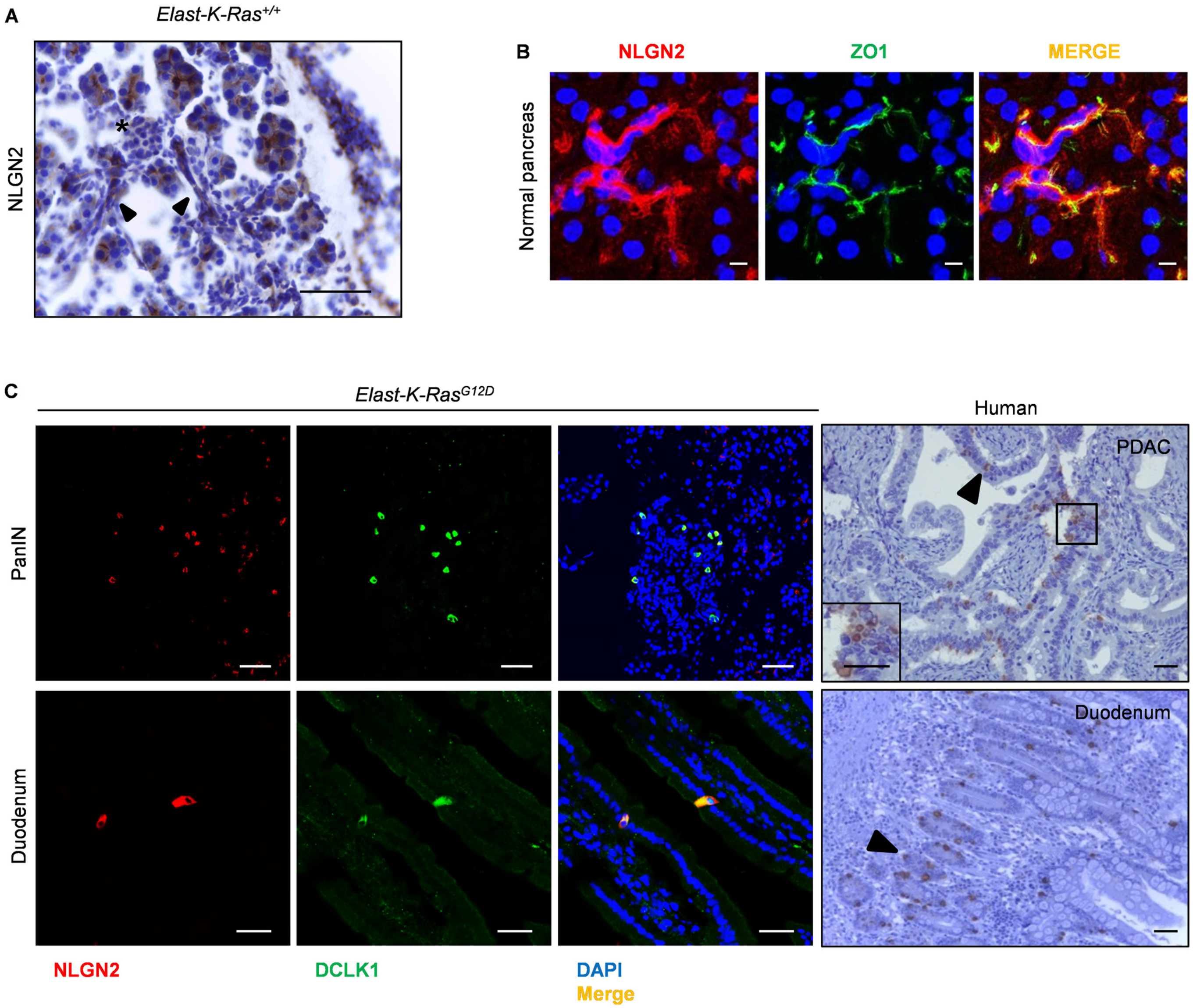

Figure EV2. NLGN2 expression pattern.

(**A**) Immunohistochemical analysis of a pancreas from a 6-month-old *Elast-K-Ras*$^{+/+}$ mouse showing NLGN2 localization in the pancreatic duct (full arrowhead) and pancreatic insulae (asterisk). (**B**) Immunofluorescence analysis of NLGN2 localization in the pancreas of 6-month-old mice. The images are representative of ductal cells of *Elast-K-Ras*$^{+/+}$ mice and untransformed ductal cells of *Elast-K-Ras*^{G12V} mice, which have superimposable aspects. NLGN2 and ZO1 are colocalized in the apical portion of pancreatic ductal cells. (**C**) Presence of a subpopulation expressing high amounts of NLGN2 in *Elast-K-Ras*^{G12V} mice (left, immunofluorescence) and in human PDAC specimens (right, immunohistochemistry). Left panels: immunofluorescence staining of PanIN (top) and duodenum tuft cells (bottom) in *Elast-K-Ras*^{G12V} mice showing a cell subpopulation expressing high levels of NLGN2, which costained with DCLK1. Right panels: immunohistochemistry revealed an NLGN2-overexpressing cell subpopulation (arrowheads) in human PDAC tissues (top, inset shows higher magnification of NLGN2^{+} cells) and in the normal duodenum (bottom). Data information: (**A**) scale bar: 0.1 mm; (**B**) scale bar: 10 μm; (**C**) scale bars: 50 μm in mouse specimens; 0.1 mm in human specimens.

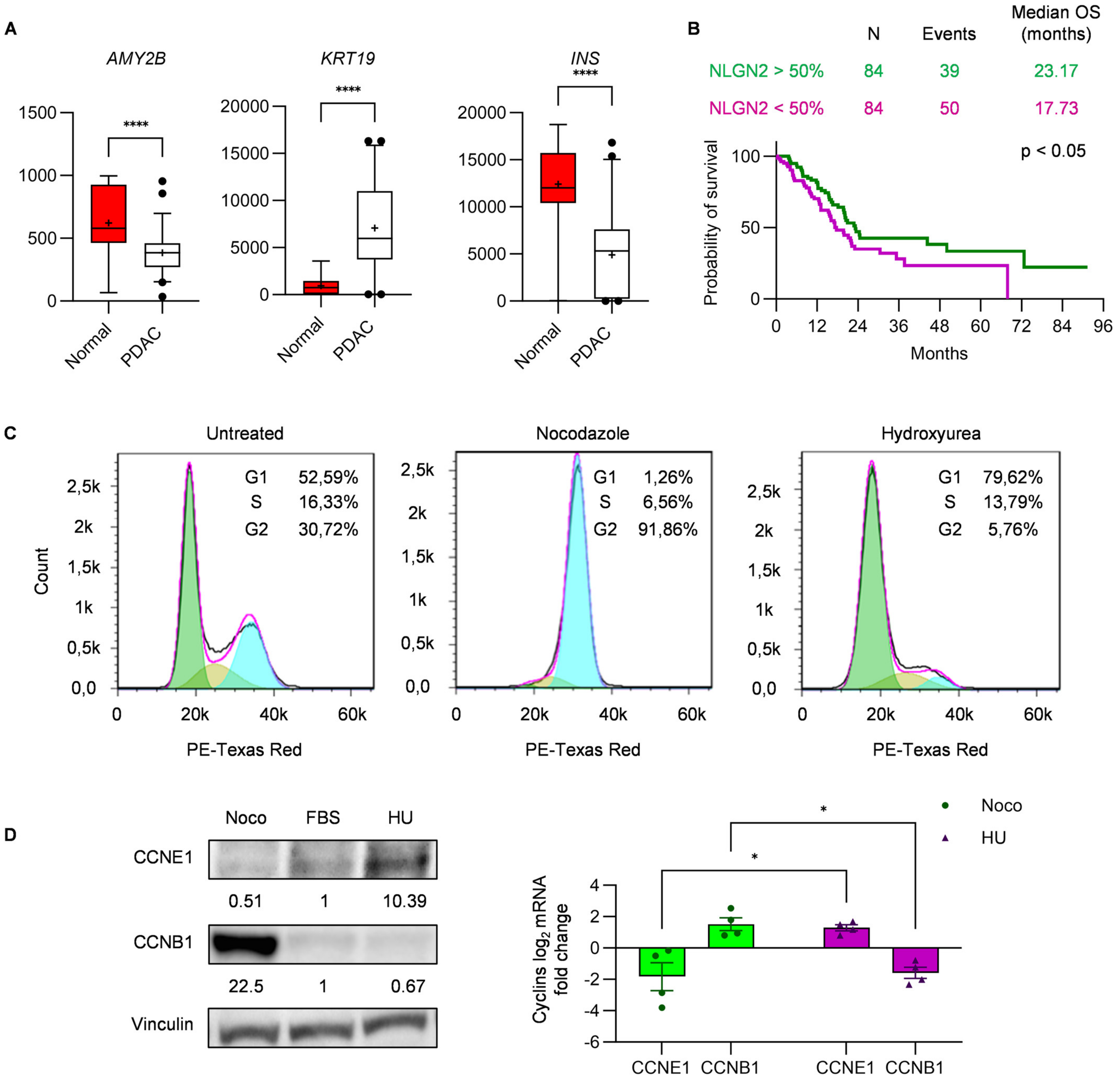

Figure EV3. Analysis of NLGN2 expression in public datasets.

(**A**) Relative differential expression of *AMY2B*, *KRT19*, and *INS* between normal pancreas and PDAC tissues as determined in the MERAV dataset. (**B**) Kaplan–Meier survival curves for *NLGN2* mRNA expression in the TCGA cohort. The panel indicates the number of events (deaths) and the median survival time, the graph show the survival probability of patients divided in half by *NLGN2* expression. (**C**) WT-HPDE cell synchronization was validated through FACS analysis, showing the DNA content (n = green peak; $2n$ = blue peak) in untreated, Noco-treated, and HU-treated cells. The percentage of cells synchronized in the G1, S, or G2 phase is embedded in each graph. (**D**) Validation of cells synchronized in G1, S, or G2 phase through measurement of cyclin E1 and B1 expression by Western blotting, reporting relative densitometry normalized on Vinculin (left) and real-time PCR (right). Data information: in (**A**) the center lines show the medians and the crosses indicate sample means; box limits indicate the 25th and 75th percentiles; the whiskers extend from the 5th and 95th percentile values and outliers are represented by dots; $n = 57$ PDAC; 16 Normal pancreas. (unpaired t-test: ****$p < 0.0001$). (**B**) OS was analyzed by the Kaplan–Meier method and compared using the log-rank test ($p < 0.05$). In (**D**) the graph shows mean mRNA $\log_2$ fold change ± SEM compared to untreated cells. Each gene was analyzed in triplicate, four biological replicates (multiple unpaired t-test: *$p < 0.05$). Source data are available online for this figure.

 © The Author(s)

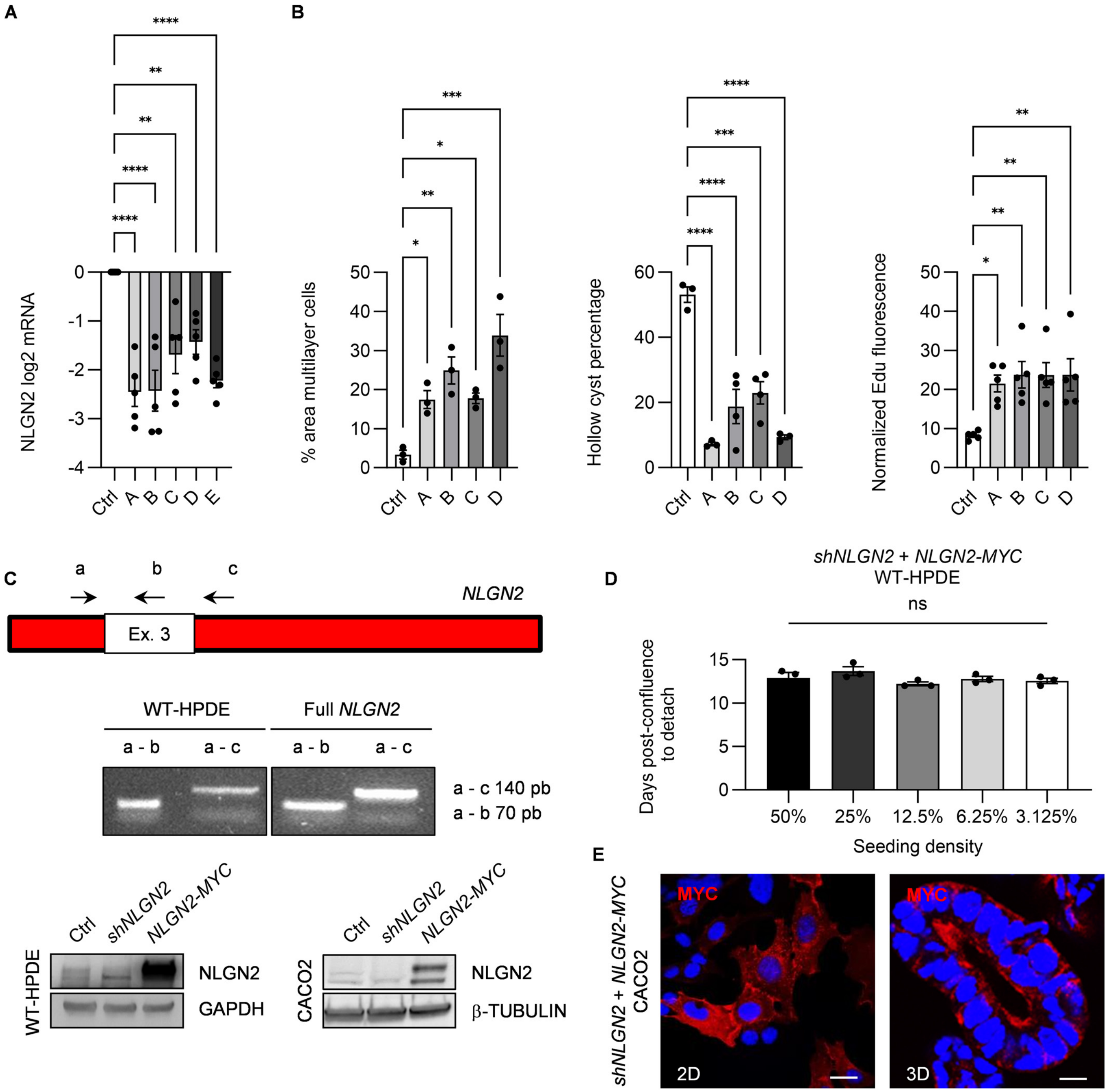
A
NLGN2 log2 mRNA
Ctrl A B C D E
B
% area multilayer cells
Hollow cyst percentage
Normalized Edu fluorescence
Ctrl A B C D
C
a b c
NLGN2
Ex. 3
WT-HPDE
Full NLGN2
a - b a - c a - b a - c
a - c 140 pb
a - b 70 pb
Ctrl shNLGN2 NLGN2-MYC
WT-HPDE
NLGN2
GAPDH
CACO2
β-TUBULIN
D
shNLGN2 + NLGN2-MYC
WT-HPDE
ns
Days post-confluence to detach
50% 25% 12.5% 6.25% 3.125%
Seeding density
E
shNLGN2 + NLGN2-MYC
CACO2
MYC
2D
3D

Figure EV4. Rescue expression of NLGN2-MYC in *shNLGN2* cells.

(**A**) Silencing efficacy of five different short hairpin RNA for *NLGN2* (**A–E**) as shown by reduced *NLGN2* mRNA expression. (**B**) Reproducibility validation of four different short hairpin RNA for *NLGN2* (**A–D**) on cell phenotype compared to Ctrl WT-HPDE. Left: the area percentage of overgrowing cells in mature confluent *shNLGN2* WT-HPDE was quantified. Mid: Cyst morphology quantification indicating the percentage of hollow cysts. Right: the graph shows EdU fluorescence normalized to the number of nuclei. (**C**) Top: Schematic representation of the PCR strategy used to identify the *NLGN2* isoform in WT-HPDE cells. The white inset represents the alternatively spliced third exon in the *NLGN2* sequence (Ex. 3). Primer pairing sites and directions are depicted with directional arrows and designated "a", "b", and "c". Mid: PCR products were obtained using primer pairs "a" and "b" or "a" and "c" on WT-HPDE cell cDNA or the recombinant full *NLGN2* control sequence, respectively. Bottom: Western blot in WT-HPDE and CACO2, showing reduced NLGN2 expression due to transduction of the *shNLGN2* carrying vector and NLGN2 re-expression in *shNLGN2* cells after transduction with the NLGN2-MYC lentiviral vector. (**D**) *shNLGN2 WT-HPDE* + NLGN2-MYC cells were seeded at different density in linear dilution, to exclude the consequences of the time needed to reach confluence. The graph shows the number of days required for detachment after confluence. (**E**) Expression of MYC-tagged NLGN2 in Ctrl and *shNLGN2* CACO2 cells is shown. The membrane and apical localization of exogenous NLGN2 was revealed using an anti-MYC antibody in 2D and 3D CACO2 cells. Data information: in (**A**) the graph reports $\log_2$ mRNA fold change ± SEM compared to Ctrl WT-HPDE of triplicate samples in five experiments (one-way ANOVA: $^{**}p < 0.01$; $^{****}p < 0.0001$). In (**B**) left: mean ± SEM of 3 biological replicates (one-way ANOVA: $^{*}p < 0.05$; $^{**}p < 0.01$; $^{***}p < 0.001$). Mid: mean ± SEM of 3 or 5 biological replicates (one-way ANOVA: $^{***}p < 0.001$; $^{****}p < 0.0001$). Right: mean ± SEM of 5 biological replicates (one-way ANOVA: $^{*}p < 0.05$; $^{**}p < 0.01$). In (**D**) the graph reports mean ± SEM, biological replicates = 3 (one-way ANOVA: non significant: ns). (**E**) Scale bar: 20 µm. Source data are available online for this figure.

 © The Author(s)

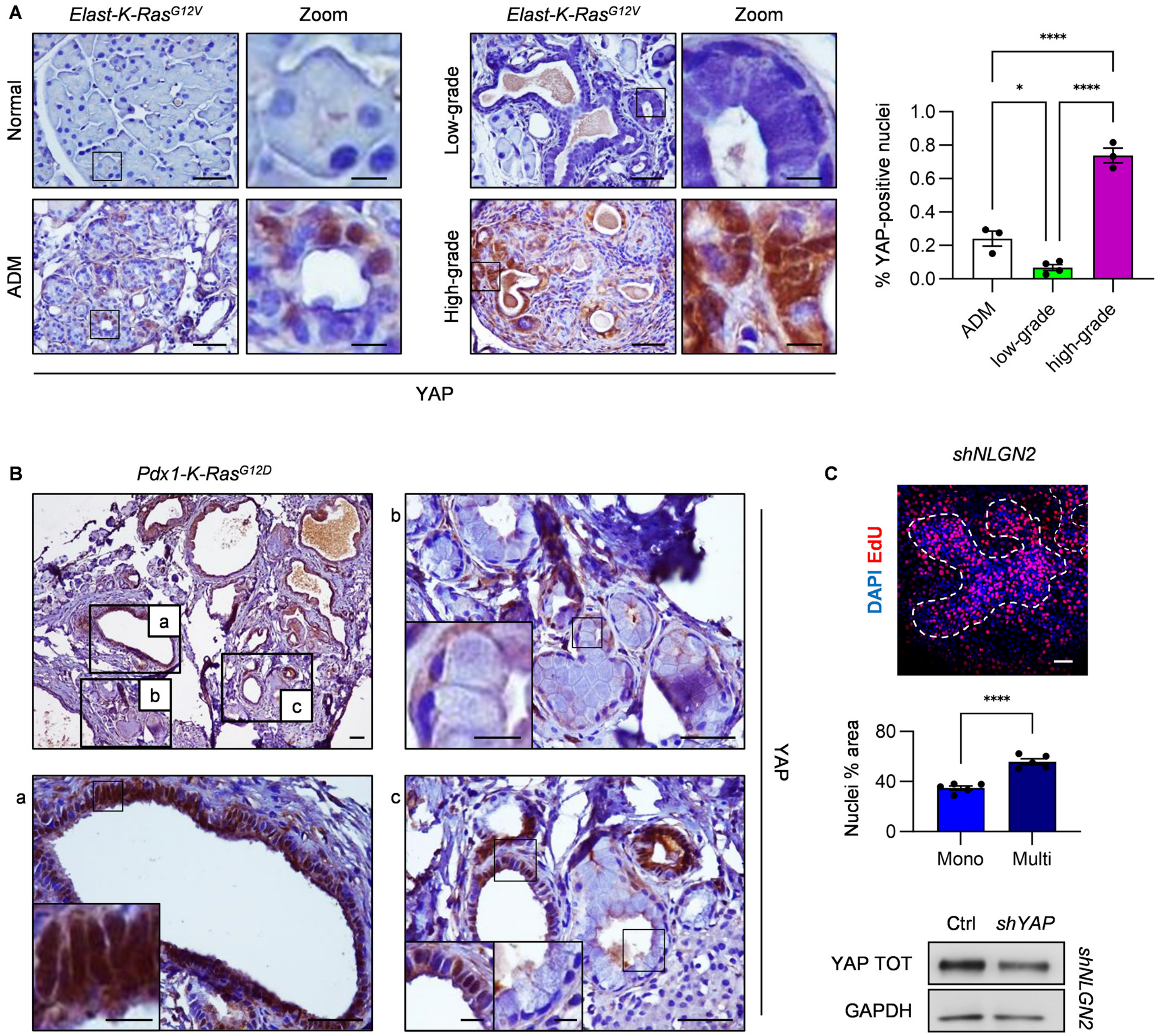

Figure EV5. YAP expression in murine ADM and PDAC.

(**A**) Immunohistochemical analysis of total YAP in normal pancreas, ADM, low-grade PanIN, and high-grade PanIN in 12-month-old *Elast-K-Ras*^{G12V} mice. The right panels show higher-magnification versions of the corresponding images (Zoom). The graph shows the percentage of YAP-positive nuclei. (**B**) Immunohistochemical analysis of total YAP in PDAC tissues of *Pdx1-K-Ras* mice. Top-left panel: the gross appearance of PDAC tissues. a: high-grade PanIN; b: low-grade PanIN; c: glands with mixed features of polarized and crowded cells exhibiting different degrees of YAP nuclear localization. (**C**) Top: Immunofluorescence of nuclei (DAPI) and EdU in *shNLGN2* mature confluent WT-HPDE. White lines distinguish monolayer and multilayer areas and highlight heterogeneity in *shNLGN2* culture. Mid graph: DAPI area in monolayer and multilayer area; Bottom: total YAP reduction in *shNLGN2* + *shYAP* mature confluent WT-HPDE. Data information: in (**A**) scale bar: 50 μm, Zoom scale bar: 10 μm. The graph shows mean percentage ± SEM; *n* mice with ADM and high-grade PanIN = 3, *n* mice with low-grade PanIN = 4; *n* of YAP-positive nuclei (ADM: *n* = 424; low-grade PanIN: *n* = 783; high-grade PanIN: *n* = 286); One-way ANOVA: $^*p < 0.05$; $^{****}p < 0.0001$. (**B**) Scale bar: 0.2 mm, (a–c) scale bar: 50 μm, inset scale bar: 10 μm. (**C**) Scale bar: 50 μm; the graph reports mean ± SEM, biological replicates = 5, unpaired *t*-test: $^{****}p < 0.0001$. Source data are available online for this figure.

© The Author(s)
